# Testing maternal effects of vitamin-D and omega-3 levels on offspring neurodevelopmental traits in the Norwegian Mother, Father and Child Cohort Study

**DOI:** 10.1017/S0033291724001466

**Published:** 2024-09

**Authors:** Robyn E. Wootton, Kyle Dack, Hannah J. Jones, Lucy Riglin, Paul Madley-Dowd, Carolina Borges, Panagiota Pagoni, Christine Roth, Anne Lise Brantsæter, Elizabeth C. Corfield, Camilla Stoltenberg, Anne-Siri Øyen, George Davey Smith, Helga Ask, Anita Thapar, Evie Stergiakouli, Alexandra Havdahl

**Affiliations:** 1 Nic Waals Institute, Lovisenberg Diaconal Hospital, Oslo, Norway; 2 MRC (Medical Research Council) Integrative Epidemiology Unit, University of Bristol, Bristol, UK; 3 Population Health Sciences, Bristol Medical School, University of Bristol, Bristol, UK; 4 School of Psychological Science, University of Bristol, Bristol, UK; 5 PsychGen Centre for Genetic Epidemiology and Mental Health, Norwegian Institute of Public Health, Oslo, Norway; 6 NIHR Bristol Biomedical Research Centre, University Hospitals Bristol and Weston NHS Foundation Trust and University of Bristol, Bristol, UK; 7 Centre for Academic Mental Health, Population Health Sciences, Bristol Medical School, University of Bristol, Bristol, UK; 8 Wolfson Centre for Young People's Mental Health and Child and Adolescent Psychiatry Section, Division of Psychological Medicine and Clinical Neurosciences, School of Medicine, Cardiff University, Cardiff, UK; 9 Department of Food Safety, Norwegian Institute of Public Health, Oslo, Norway; 10 Department of Global Public Health and Primary Care, University of Bergen, Bergen, Norway; 11 PROMENTA Research Center, Department of Psychology, University of Oslo, Oslo, Norway

**Keywords:** ADHD, autism, DHA, father and child cohort study, intrauterine, mendelian randomization, MoBa, neurodevelopment, omega-3, polygenic, the Norwegian mother, vitamin-D

## Abstract

**Background:**

Maternal vitamin-D and omega-3 fatty acid (DHA) deficiencies during pregnancy have previously been associated with offspring neurodevelopmental traits. However, observational study designs cannot distinguish causal effects from confounding.

**Methods:**

First, we conducted Mendelian randomisation (MR) using genetic instruments for vitamin-D and DHA identified in independent genome-wide association studies (GWAS). Outcomes were (1) GWAS for traits related to autism and ADHD, generated in the Norwegian mother, father, and child cohort study (MoBa) from 3 to 8 years, (2) autism and ADHD diagnoses. Second, we used mother–father–child trio-MR in MoBa (1) to test causal effects through maternal nutrient levels, (2) to test effects of child nutrient levels, and (3) as a paternal negative control.

**Results:**

Associations between higher maternal vitamin-D levels on lower ADHD related traits at age 5 did not remain after controlling for familial genetic predisposition using trio-MR. Furthermore, we did not find evidence for causal maternal effects of vitamin-D/DHA levels on other offspring traits or diagnoses. In the reverse direction, there was evidence for a causal effect of autism genetic predisposition on lower vitamin-D levels and of ADHD genetic predisposition on lower DHA levels.

**Conclusions:**

Triangulating across study designs, we did not find evidence for maternal effects. We add to a growing body of evidence that suggests that previous observational associations are likely biased by genetic confounding. Consequently, maternal supplementation is unlikely to influence these offspring neurodevelopmental traits. Notably, genetic predisposition to ADHD and autism was associated with lower DHA and vitamin-D levels respectively, suggesting previous associations might have been due to reverse causation.

## Introduction

Neurodevelopmental conditions include attention-deficit hyperactivity disorder (ADHD) and autism. They are characterized by early-onset delays and differences in developmental domains such as language and motor skills, social communication, flexibility, attention, and activity regulation (Thapar, Cooper, & Rutter, 2017). Neurodevelopmental traits are normally distributed throughout the population, and are etiologically complex, involving a large number of genetic and environmental predisposing factors (Thapar et al., 2017). One proposed modifiable environmental predisposing factor is maternal nutritional deficiencies during pregnancy (Georgieff, Ramel, & Cusick, 2018). Many prenatal micronutrient deficiencies have been associated, including vitamin-D levels and omega-3 fatty acids (specifically Docosahexaenoic acid, DHA) (García-Serna & Morales, 2019; Nevins et al., 2021). Nutrient availability during the prenatal period plays an important role in brain development. For example, vitamin-D receptors are present across several regions of the neonatal brain (Eyles, Smith, Kinobe, Hewison, & McGrath, 2005) and play a role in crucial processes of brain development including neuronal differentiation and axonal connectivity (Eyles, Burne, & McGrath, 2013). DHA (along with other long-chained polyunsaturated fatty acids) is necessary for processes of brain development including neurogenesis, synaptogenesis, and neuronal migration (Innis, 2008). Therefore, maternal deficiencies for both vitamin-D and DHA have been proposed to increase likelihood of neurodevelopmental conditions through changes to the structure or functions of the developing fetal brain.

However, results from observational studies are inconsistent and can be biased by residual environmental or genetic confounding and reverse causation (García-Serna & Morales, 2019; Nevins et al., 2021). Thus, the causal role of these exposures on neurodevelopmental traits is unclear. Furthermore, it is difficult to use RCTs to investigate the effects of intrauterine exposures as it is often unethical or impractical to modify levels experimentally, and long-term follow-up is expensive and time-consuming. A systematic-review of RCTs for DHA supplementation during pregnancy showed very little evidence for an effect on offspring neurodevelopment (Nevins et al., 2021). For vitamin-D, only one such RCT exists, which reported that third-trimester supplementation did not associate with neurodevelopmental outcomes up to 6 years (Sass et al., 2020). Overall, the current RCT evidence is low quality (consisting of under representative and small samples) (Nevins et al., 2021).

Mendelian randomization (MR) is a complementary approach to assess causality in observational studies (Carnegie et al., 2020). MR examines whether exposure–outcome relationships are consistent with causality using genetic instruments as proxies for modifiable exposures (Sanderson et al., 2022). Instead of statistically randomizing individuals to receive the exposure (e.g. supplementation) or control, MR makes use of naturally occurring random variation in genetic variants, which influence levels of the exposure (e.g. higher vitamin-D or DHA levels) (Carnegie et al., 2020). MR allows for unbiased estimation of causal effects provided that the following assumptions are satisfied: (1) the genetic variants are robustly associated with the exposure; (2) there is no confounding of the genetic instrument and the outcome; and (3) the genetic variants only influence the outcome through the exposure (Sanderson et al., 2022). Assumptions can be violated by horizontal pleiotropy, where genetic instruments for the exposure are associated with other traits that are not on the causal pathway from exposure to outcome. Primers on the MR method in general and in application to nutritional psychiatry are available elsewhere (Carnegie et al., 2020; Davies, Holmes, & Smith, 2018; Sanderson et al., 2022). Two previous MR studies have explored the impact of vitamin-D levels on neurodevelopment, finding no strong evidence for a causal effect on autism (Revez et al., 2020) nor ADHD (Libuda et al., 2021).

In the current study, we aim to test the possible causal effect from mothers on their offspring. Such a causal question requires a further extension to the MR design to account for shared genetic predisposition. Without such adjustment, MR estimates could reflect a direct intrauterine effect, but the association could alternatively be due to genetic variants directly transmitted from the mother to offspring that influence the child's own nutrient levels (Lawlor et al., 2017). Here, we extend the standard MR approach using mother–father–child family data in a trio-MR design. We control for mediation via offspring genotype to isolate the effect through the maternal genotype (as applied elsewhere: [Moen et al., 2020; Taylor et al., 2023]). This approach is not common, given the need for large samples of at least mother-offspring pairs (Evans, Moen, Hwang, Lawlor, & Warrington, 2019). Here it was possible thanks to the recent release of large-scale trio data from the Norwegian Mother, Father and Child Cohort Study (MoBa). Having access to father's genotype data provides two additional benefits: (1) adjusting for father's genotype prevents collider bias (Lawlor et al., 2017) and (2) fathers provide a negative control to explore whether the maternal effects are likely pregnancy-related or explained by early life environments provided by the parents. One MR study to date has explored maternal effects of vitamin-D on offspring autism accounting for child genetic liability which did not support the presence of a causal effect (Madley-Dowd et al., 2022). To the best of our knowledge, there are no other MR studies of vitamin-D nor DHA on neurodevelopmental outcomes using designs able to account for shared genetic liability.

If associations between maternal nutrient levels and offspring neurodevelopmental traits are not due to causal effects, an alternative explanation could be reverse causation. Evidence suggests that individuals with a genetic predisposition to neurodevelopmental conditions eat more restricted diets (Smith, Rogers, Blissett, & Ludlow, 2020), which could in turn lead to lower nutrient levels. Mothers with a genetic predisposition to neurodevelopmental conditions might therefore have lower nutrient levels, and (independently of nutrient levels) pass on a genetic predisposition to neurodevelopmental conditions to their offspring. We estimate possible reverse causation using an MR design, this time using genetic predisposition to ADHD and autism diagnoses as the exposure, and vitamin-D and DHA levels as the outcome.

In this study we used maternal genetically predicted vitamin-D and DHA levels to estimate the causal effects of maternal vitamin-D and DHA on offspring neurodevelopment, while accounting for both offspring and paternal genetics. Second, we used paternal genetically predicted vitamin-D and DHA levels as a negative control to explore whether maternal effects are likely pregnancy-related. Third, we used MR to test for possible reverse causation, estimating causal effects of genetic predisposition to ADHD and autism on vitamin-D and DHA levels.

## Methods

### Sample

MoBa is a population-based pregnancy cohort study conducted by the Norwegian Institute of Public Health (Magnus et al., 2016). Participants were recruited from across Norway from 1999 to 2008. The women consented to participate in 41% of pregnancies. The cohort includes 114 500 children, 95 200 mothers, and 75 200 fathers. The current study is based on version 12 of the quality-assured data released for research in January 2019. Biological material, including DNA samples were collected from both parents during pregnancy and from children (umbilical cord) at birth (Paltiel et al., 2014).

Details of the genotyping and QC procedures in MoBa are available elsewhere (Corfield et al., 2022). After restricting to unrelated individuals between trios (proportion of the genome shared identity-by-descent < 0.15) of European ancestry with genotype data passing QC and phenotype data available, we had 23 713 mothers, 17 990 fathers, 26 646 children, and 16 298 trios (N genotyped individuals/trios for each outcome in online Supplementary Table S1).

### Exposures

#### Vitamin-D

A GWAS of 401 460 individuals (Manousaki et al., 2020) identified 38 genome-wide significant single nucleotide polymorphisms (SNPs) that explained 3.1% variance in an independent sample. These variants have been validated for use in pregnancy, in the UK Avon Longitudinal Study of Parents and Children (ALSPAC) where they explained 1.05% of the variance (Madley-Dowd et al., 2022). Another GWAS of Vitamin-D (Revez et al., 2020) published at the same time was used as a sensitivity analysis (online Supplementary Note S1). Given possible pleiotropy (Fang, Zhao, Yang, Zhang, & Giovannucci, 2024) we also conducted a sensitivity analysis using SNPs with known functional effects on vitamin-D levels (online Supplementary Note S4).

#### DHA

We used the largest available GWAS of DHA which identified 61 genome-wide significant SNPs in a sample of 114 999 individuals (Borges et al., 2022). We estimated that these SNPs explain 6.6% of the variance in the discovery sample. To validate these SNPs for use during pregnancy, we tested their prediction in the ALSPAC cohort (online Supplementary Note S2), where they explained 0.85% of variance in DHA levels during pregnancy. We also conducted a sensitivity analysis using SNPs with known functional effects on DHA levels (online Supplementary Note S4).

### Outcomes in MoBa

We focused on clinically relevant neurodevelopmental traits, reported by the mothers at child ages 3, 5, and 8 years (further details in online Supplementary Note S3):

*ADHD-related traits* of inattention and hyperactivity/impulsiveness were assessed using two scales: a reduced Child-Behavior Checklist at 3 and 5 years (Achenbach, 1992), and the Disruptive Behaviour Disorder Rating Scale (RS-DBD) (Silva et al., 2005) at 8 years.

*Autism-related traits* of social communication differences and restricted and repetitive behaviors and interests (RRB) were assessed at 3 and 8 years using the Social Communication Questionnaire (Rutter, Bailey, & Lord, 2003).

*Language delay* was measured using the Ages and Stages Questionnaire (ASQ) (Squires, Potter, & Bricker, 1999) language items at ages 3 (6-items) and 5 years (7-items) years.

*Motor delay* was measured by the ASQ at age 3 (4-items) and by the Child Development Inventory at age 5 years (12-items).

### Statistical analysis

We conducted two different methods to strengthen causal inference: two-sample MR and a trio-MR analysis both using genome-wide significant SNPs (*p* < 5 × 10^−8^). Online Supplementary Fig. S11 provides an overview of the analyses conducted. We performed a Bonferroni adjustment for the number of tests conducted in the primary analyses (*p* = 0.05/(19 × 2) = 0.001).

### Analysis 1 – Two-sample MR estimates of nutrient levels on offspring neurodevelopment

In MR analysis, we use genetic variants as instruments to estimate a causal effect of an exposure on an outcome. In two-sample MR, estimates of the SNP-exposure and SNP-outcome effects come from summary statistics of two independent GWAS (Hartwig, Davey Smith, & Bowden, 2017). As outlined in the introduction, three core assumptions must hold for valid causal inference (Davies et al., 2018). To satisfy assumption 1, we selected only independent genome-wide significant SNPs, where predictive validity during pregnancy had been established (online Supplementary Note S2). SNPs passing QC were clumped for independence (*r*^2^ < 0.001, 10 000 kilobases). Measures of nutrient levels were not available for pregnant mothers in the MoBa cohort, so genetic variants were validated as a robust predictor of nutrient levels during pregnancy in the ALSPAC cohort (see online Supplementary Note S2 and [Madley-Dowd et al., 2022]). Furthermore, we conducted a sensitivity analysis using only SNPs for vitamin-D and DHA where biological function is understood (online Supplementary Note S4).

SNP-outcome effects using maternal genotype data were estimated in MoBa with linear regression (adjusted for 10 principal components (PCs) to account for population structure and genotype batch). Our primary method for estimating causal effects was the inverse–variance weighted (IVW) estimator. To explore the validity of assumptions 2 and 3, we conducted three additional sensitivity methods with different assumptions about the nature of possible pleiotropy: weighted median (Bowden, Davey Smith, Haycock, & Burgess, 2016a), MR Egger (Bowden, Davey Smith, & Burgess, 2015), and weighted mode (Hartwig et al., 2017) (see online Supplementary Note S5 for further details). The MR Egger intercept provides an estimate of bias from directional horizontal pleiotropy (Bowden et al., 2015). Heterogeneity of genetic instruments was assessed using Cochrans *Q*, and instrument strength was checked using the *F* statistic (*F* > 10 indicates low risk of weak instrument bias). Simulation-extrapolation MR was performed if violations of the no measurement error assumption were detected from *I*^2^_GX_ (Bowden et al., 2016b)_._ Where there was evidence for a causal effect, we conducted leave-one-out-analysis to identify potential outliers and Steiger filtering to test for possible reverse causation (Hemani, Tilling, & Davey Smith, 2017) (online Supplementary Fig. S11, Analysis 1.1).

We conducted a replication of our MoBa MR analysis using GWAS summary statistics from the Psychiatric Genomics Consortium (PGC) for autism (Grove et al., 2019) and ADHD (Demontis et al., 2019) diagnoses. The GWAS of autism comprised 18 381 cases and 27 969 controls (Grove et al., 2019). The GWAS of ADHD comprised 20 183 cases and 35 191 controls (Demontis et al., 2019) (online Supplementary Fig. S11, Analysis 1.2).

We conducted a sensitivity analysis using child genotype for SNP-outcome effects to test for effects of child's own nutrient levels on their neurodevelopmental traits (online Supplementary Fig. S11, Analysis 1.3). Our power calculation demonstrated that the genetic instruments provided sufficient power for MR analyses (online Supplementary Note S6).

### Analysis 2 – Trio-MR analysis in MoBa

We used independent (*r*^2^ = 0.25, 500 kb) genome-wide significant SNPs from the respective GWAS summary statistics to construct PGS. PGS were computed as the weighted sum of effect alleles for the respective exposure for MoBa mothers, fathers and children using PRSice (Euesden, Lewis, & O'Reilly, 2015).

First, we looked at the effect of maternal PGS on neurodevelopmental outcomes, with adjustment for offspring and paternal genotype to determine if the observed association is due to maternal causal effects, genetic confounding or bias. These analyses were adjusted for child sex, population structure (using the first 10 PCs), and genotype batch. Adjusting for the child PGS alone risks opening up a back-door path through paternal genotype, creating an additional source of bias. Therefore, we also adjusted for paternal genotype (online Supplementary Fig. S11, Analysis 2.1). Due to different rates of diagnoses in males and females, we also conducted analyses stratified by child sex.

Additionally, we explored possible effects of child vitamin-D and DHA levels on child neurodevelopmental traits. Here, our model first looked at the association of child PGS for vitamin-D and DHA only, then additionally adjusted for maternal and paternal PGS (online Supplementary Fig. S11, Analysis 2.2).

Finally, we conducted a paternal negative control analysis. If we hypothesize that causal effects are maternal specific (e.g. through the intrauterine environment), rather than through other environmental effects (such as nutrition provided to the child in early life), then we would expect to see an effect of maternal PGS, but not paternal PGS. We therefore repeated analyses using the paternal PGS only and subsequently controlled for the maternal and child PGS (online Supplementary Fig. S11, Analysis 2.3). We performed a sensitivity analysis to check for assortative mating on vitamin-D and DHA levels in the MoBa cohort by looking at the correlation between maternal and paternal PGS.

### Analysis 3 – Two-sample MR testing for possible reverse causation of neurodevelopment on nutrient levels

SNP-exposure estimates were obtained from the PGC GWAS of autism (Grove et al., 2019) and ADHD diagnoses (Demontis et al., 2019). We used the same GWAS of vitamin-D and DHA, this time as the outcome (see online Supplementary Fig. S11, 3.1). Given the small number of genetic instruments available, we used a relaxed *p* value threshold of *p* < 5 × 10^−6^, resulting in 34 SNPs for autism and 63 SNPs for ADHD. In this direction, we report effect sizes as the average change in the outcome (nutrient levels) per doubling (2-fold increase) in the liability to the exposure (ADHD or autism) (Burgess & Labrecque, 2018; Howe, Tudball, Davey Smith, & Davies, 2022).

## Results

### Testing for causal effects of DHA and vitamin-D on neurodevelopmental traits in MoBa

**Figure 1.**
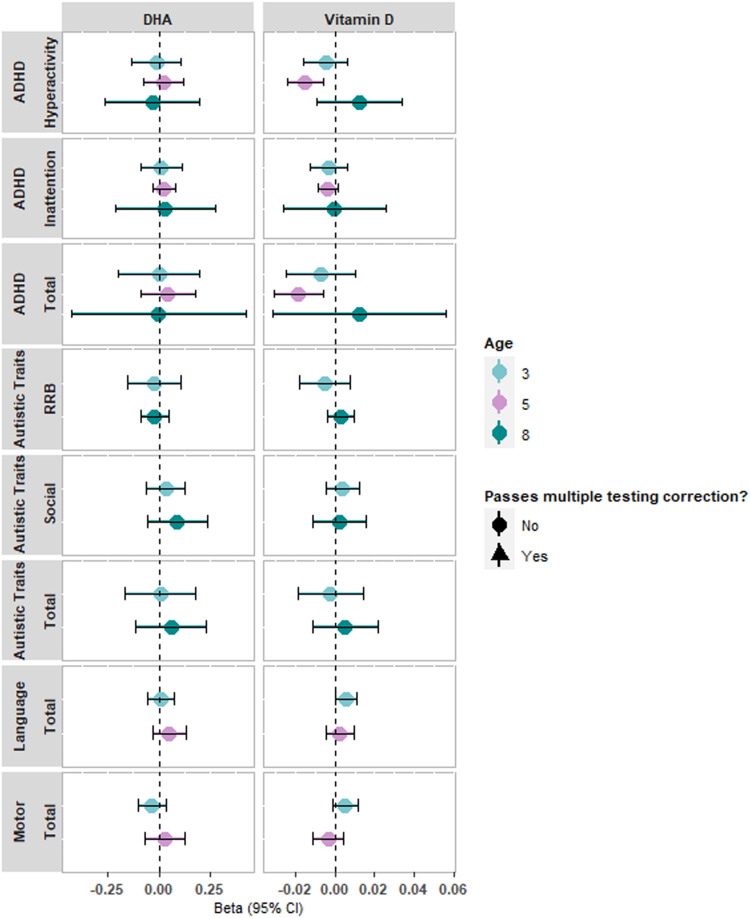
Two-sample MR results for vitamin-D and DHA on neurodevelopmental outcomes in the MoBa sample. All estimates are from the inverse-variance weighted method. Units can be interpreted as per s.d. increase in exposure on the scale of the outcome traits.

Two-sample MR analysis in the MoBa cohort provided little evidence for a causal effect of vitamin-D or DHA on any neurodevelopmental traits after adjustment for multiple testing (Fig. 1, online Supplementary Table S2). There was weak evidence for an effect of higher maternal vitamin-D levels on decreased ADHD hyperactivity traits at age 5 years (−0.015, 95% CI −0.024 to −0.006) and total ADHD-related traits at age 5 years (−0.018, 95% CI −0.030 to −0.006). These effects were consistent in direction, with similar effect sizes across MR sensitivity methods but did not survive correction for multiple testing (online Supplementary Table S2). SNPs had sufficient instrument strength (online Supplementary Table S3) and there was minimal evidence of regression dilution bias, suggesting suitability of the MR Egger method (online Supplementary Table S3). There was little evidence of heterogeneity (online Supplementary Table S4), and the MR Egger intercept did not suggest bias from horizontal directional pleiotropy (online Supplementary Table S5). There was similarly weak evidence for causal effects using canonical SNPs for vitamin-D and DHA (see online Supplementary Note S4 for results and discussion).

### Two-sample Mendelian randomization analyses using child genotypes

There was very limited evidence for causal effects of genetic predisposition to DHA or vitamin-D on any of the neurodevelopmental traits (online Supplementary Table S6). There was some weak evidence for an effect of child vitamin-D levels on decreased ADHD hyperactivity traits at age 5 years (−0.014, 95% CI −0.022 to −0.005), total ADHD traits at age 5 years (−0.017, 95% CI −0.029 to −0.005), and restrictive-repetitive behaviors at age 3 years (−0.017, 95% CI −0.028 to −0.006). There was weak evidence for an effect of DHA levels on decreased ADHD hyperactivity traits at age 8 years (−0.310, 95% CI −0.553 to −0.066) and decreased restrictive and repetitive behaviors at age 8 years (−0.078, 95% CI −0.153 to −0.003). These effects had consistent direction and relatively similar effect sizes across MR sensitivity methods (online Supplementary Table S2) but did not survive correction for multiple testing.

### Testing for causal effects of DHA and vitamin-D on autism and ADHD diagnoses

**Figure 2.**
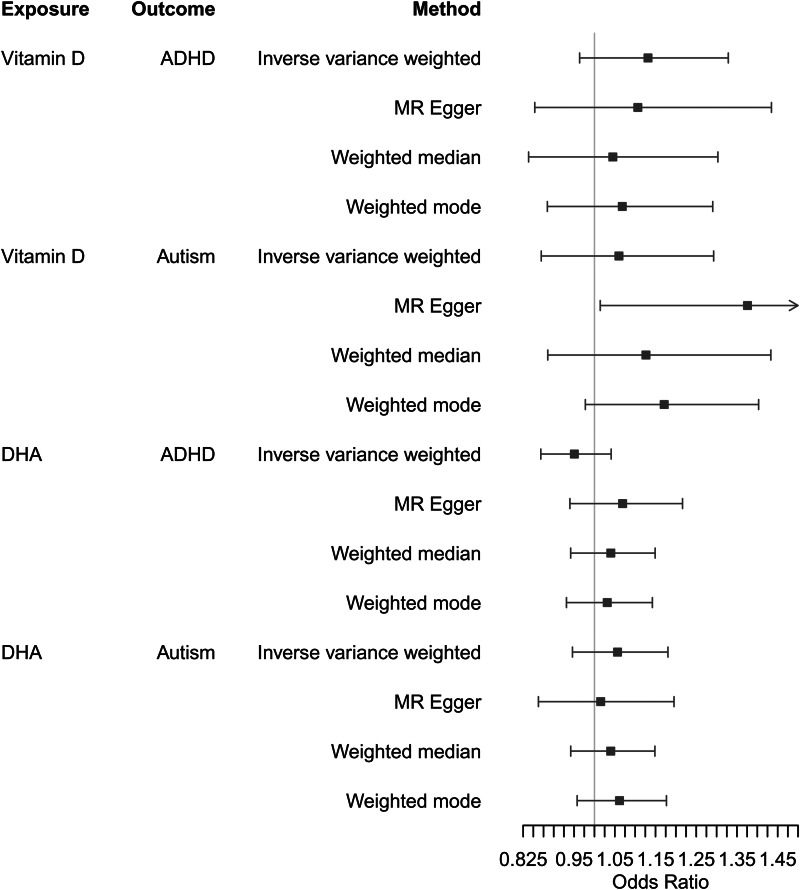
Two-sample MR results for vitamin-D and DHA on diagnoses of ADHD and autism. All units can be interpreted as per s.d. increase in exposure on the odds of outcome diagnosis.

We observed little evidence for causal effects of either vitamin-D or DHA on autism or ADHD diagnoses (Fig. 2, online Supplementary Table S7). *F* statistics were greater than 10 suggesting no weak instrument bias (online Supplementary Table S8). There was evidence of heterogeneity (online Supplementary Table S9), and the MR Egger intercepts suggested possible bias from horizontal pleiotropy for DHA on ADHD and vitamin-D on autism (online Supplementary Table S10).

### Testing maternal effects using trio-MR

**Figure 3.**
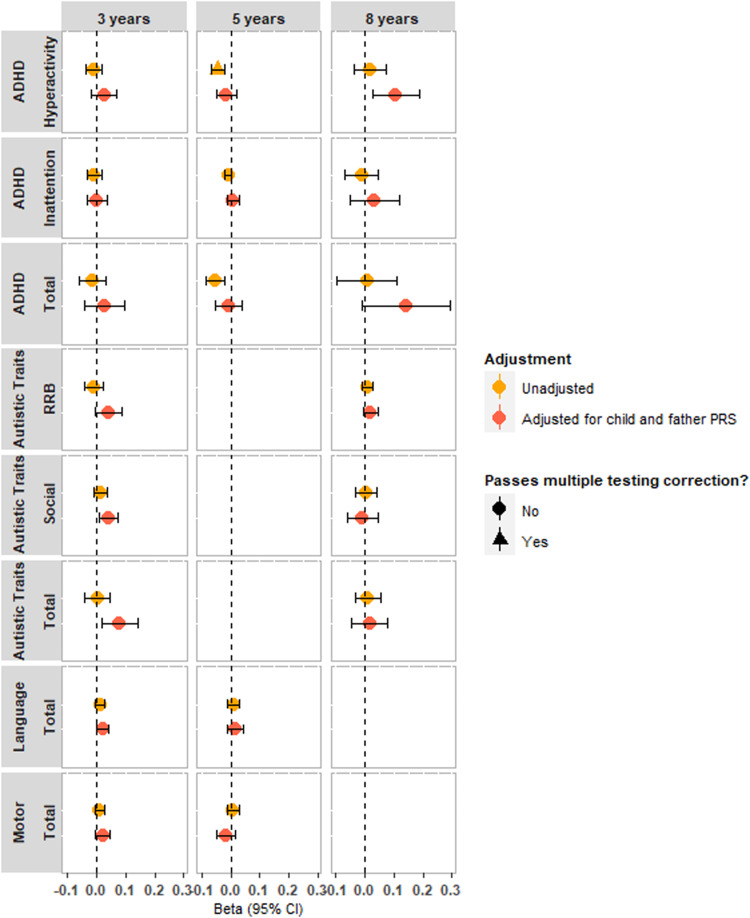
Trio-Mendelian randomization analysis of maternal polygenic score for vitamin-D levels on neurodevelopmental outcomes in the MoBa sample, with and without adjustment for child and paternal polygenic scores. All units can be interpreted as per s.d. increase in polygenic score on the scale of the outcome traits.

**Figure 4.**
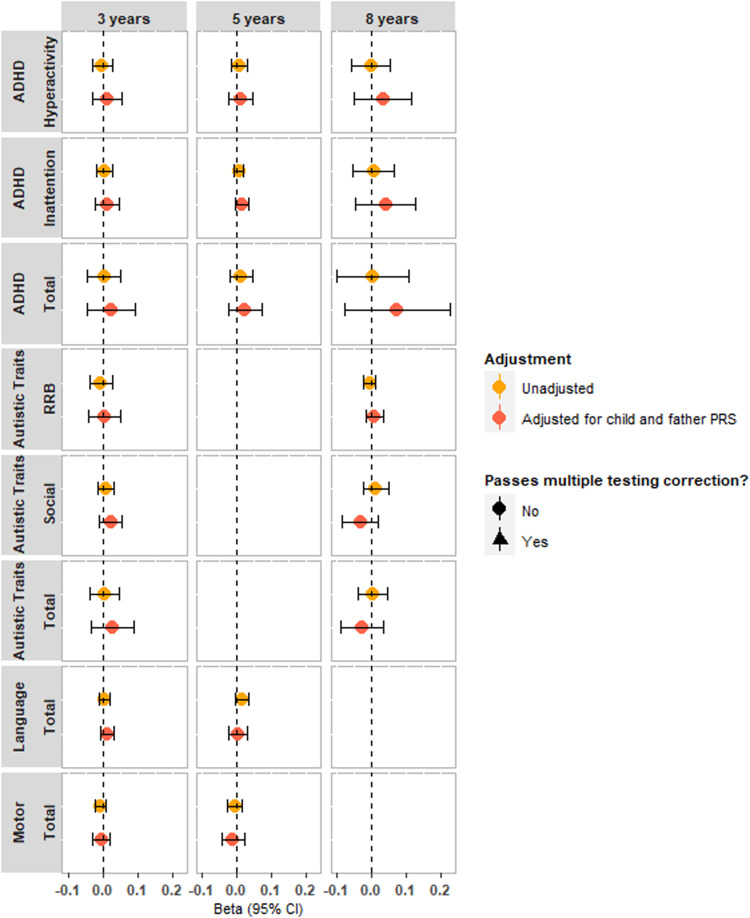
Trio-Mendelian randomization analysis of maternal polygenic score for DHA levels on neurodevelopmental outcomes in the MoBa sample, with and without adjustment for child and paternal polygenic scores. All units can be interpreted as per s.d. increase in polygenic score on the scale of the outcome traits.

We did not find evidence to support maternal effects of vitamin-D or DHA on any offspring neurodevelopmental traits after adjusting for child and paternal PGS (Figs 3 and 4, online Supplementary Table S11). Prior to adjustment, there was evidence that maternal vitamin-D PGS was associated with offspring ADHD hyperactivity traits at age 5 years (−0.045, 95% CI −0.070 to −0.021, Fig. 3, online Supplementary Table S13) but this association was attenuated after controlling for child and father PGS (−0.016, 95% CI −0.051 to 0.019) (online Supplementary Table S11). Correlations between maternal and paternal PGS were weak (DHA: *r* = 0.006, 95% CI −0.005 to 0.016, *p* = 0.29; vitamin-D: *r* = −0.004, 95% CI −0.015 to 0.006, *p* = 0.42) suggesting results are unlikely biased by assortative mating. Results stratified by child sex did not show evidence for association (online Supplementary Figs S3 and S4, Tables S12 and S13).

### Testing effects of child's nutrient levels using trio-MR

There was little evidence for associations between child DHA PGS and any neurodevelopmental traits (online Supplementary Fig. S5, Table S14). Prior to adjustment, there was some evidence for an association between child PGS for higher vitamin-D levels and lower hyperactivity scores at age 5 years (−0.046, 95% CI −0.068 to −0.023) (online Supplementary Fig. S6, Table S14) but this was somewhat attenuated after adjustment for the parental polygenic scores (−0.035, 95% CI −0.075 to 0.005). There was strong evidence for an association between child PGS for higher vitamin-D levels and lower total ADHD traits at both age 3 (−0.049, 95% CI −0.093 to −0.005) and age 5 years (−0.054, 95% CI −0.085 to −0.023), the latter of which was only partially attenuated after adjustment for parental PGS (3 years: −0.068, 95% CI −0.148 to 0.011; 5 years: −0.058, 95% CI −0.112 to −0.003). Finally, there was strong evidence for an association between child PGS for higher vitamin-D levels and lower restrictive and repetitive behaviors at age 3 (−0.043, 95% CI −0.073 to −0.013) which again was attenuated by adjustment (−0.027, 95% CI −0.081 to 0.027).

### Paternal negative control analysis

We did not find evidence for associations between paternal PGS and child neurodevelopmental outcomes before or after adjustment for maternal and child PGS (online Supplementary Figs S7 and S8, Table S15).

### Testing for possible reverse causation using PGC diagnostic GWAS

**Figure 5.**
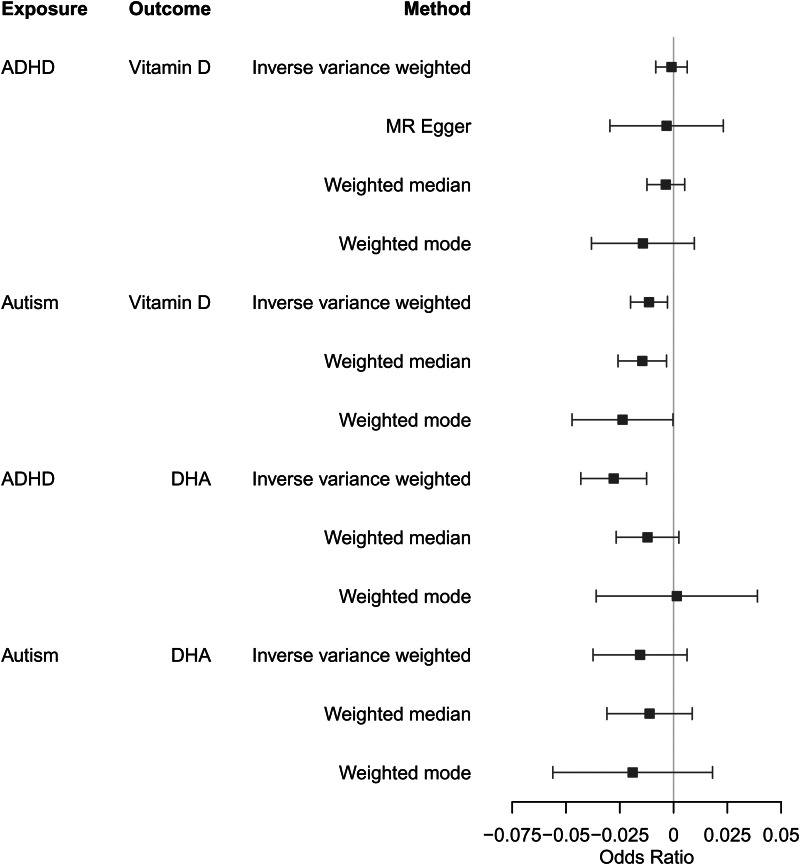
Two-sample MR results for genetic predisposition to ADHD and autism on vitamin-D levels and DHA. Units can be interpreted as the average change in standardized units of the outcome per doubling (2-fold increase) in the prevalence of the exposure.

There was consistent evidence for a causal effect of higher genetic predisposition to autism on lower vitamin-D levels across sensitivity methods (IVW: −0.011 unit increase in vitamin-D per doubling of autism odds, 95% CI −0.020 to −0.003; Fig. 5, online Supplementary Table S7). There was some evidence for an effect of higher genetic predisposition to ADHD on lower DHA levels (IVW: −0.028), (95% CI −0.043 to −0.013), which was consistent for weighted median, but not weighted mode (Fig. 5, online Supplementary Table S7). We did not conduct MR Egger due to substantial regression dilution bias with the exception of ADHD on vitamin-D (online Supplementary Table S8). *F* statistics suggested that instruments were not weak (online Supplementary Table S8). For autism on vitamin-D, all 34 SNPs explained more variance in the exposure than the outcome, suggesting reverse causation was unlikely. For ADHD on DHA, all but one of the 64 SNPs explained more variance in the exposure than the outcome, again suggesting reverse causation was unlikely. Iteratively removing genetic variants provided virtually the same results (online Supplementary Figs S1 and S2).

## Discussion

Across two different study designs (two-sample MR and trio-MR), the evidence did not support causal effects of maternal vitamin-D or DHA levels on offspring neurodevelopmental outcomes (both traits and diagnoses). Trio-MR analysis suggested that two-sample MR results were biased by shared genetic liability, as associations between maternal vitamin-D and offspring ADHD-related traits at age 5 were attenuated once child and father genetic predisposition had been accounted for. This suggests that associations were largely explained by genetic confounding. Similarly, triangulating across our different study designs and samples, it seems likely that observed associations do not reflect maternal causal effects, highlighting the benefits of using a genetically informed family-based cohort.

The current findings add to a growing body of evidence that suggests that if maternal effects of vitamin-D or DHA deficiencies exist for neurodevelopmental outcomes, that they are likely to be small in magnitude. Randomized control trials, MR, trio-MR, paternal negative control studies, and family studies all have different sources of bias, hence we can strengthen our causal inferences by comparing evidence across them (Munafò, Higgins, & Davey Smith, 2021). Randomized control trials have found limited evidence for intrauterine effects (Nevins et al., 2021; Sass et al., 2020) and previous MR studies of vitamin-D did not find evidence for an effect (Libuda et al., 2021; Madley-Dowd et al., 2022; Revez et al., 2020). This is the first study using a trio-MR analysis to explore possible maternal effects of vitamin-D and DHA on neurodevelopment, while accounting for shared genetics among family members. Consistent with previous findings, our results did not support a causal maternal effect after controlling for shared genetic predisposition. Triangulating evidence across these diverse study designs, with different sources of bias, we conclude that causal maternal effects of vitamin-D and DHA on offspring neurodevelopmental outcomes are unlikely to play a significant role. Consequently, supplementation during pregnancy is unlikely to influence predisposition to neurodevelopmental difficulties in the offspring.

Observational associations between maternal nutrient levels and offspring neurodevelopment could instead be due to the influence of child's own nutrient levels. Maternal and child nutrient levels are likely to be correlated through shared diet and shared genetic predisposition. Nutrient levels during early childhood could be more important for neurodevelopmental outcomes than levels during pregnancy. Previous studies have found associations between child nutrient levels and child neurodevelopmental outcomes (Gan, Galer, Ma, Chen, & Xiong, 2019; Li et al., 2020; Ryan et al., 2010). For example, a meta-analysis of RCT studies of child vitamin-D supplementation showed very small improvements in ADHD scores (Gan et al., 2019) and a review of vitamin-D supplementation studies in children with autism showed a small improvement in hyperactivity scores (Li et al., 2020). A review of DHA supplementation studies in children reported mixed evidence, with some improvement in cognitive outcomes, and a need for higher quality study designs (Ryan et al., 2010). In the current study, we found some evidence that the association of child's vitamin-D levels with ADHD and autism-related traits could be due to shared genetic predisposition in trio-MR analyses.

Previous observational associations between maternal nutrient levels and offspring neurodevelopment could instead be due to reverse causation. Our bi-directional MR found evidence for genetic predisposition to autism predicting lower vitamin-D levels and genetic predisposition to ADHD predicting lower DHA levels. Individuals with autism often exhibit picky eating and resistance to new foods leading to a restricted diet (Guo et al., 2019), which might lead to nutritional deficiencies during pregnancy. Genetic predisposition to neurodevelopmental conditions in the mother will also increase odds of neurodevelopmental conditions in the child through both direct and indirect genetic transmission. This transmission might appear as an association between nutrient levels and neurodevelopmental outcomes when shared genetic predisposition and reverse causation are not sufficiently accounted for in study design. Emerging evidence supports the hypothesis that neurodevelopmental genetic predisposition precedes difference in nutrient levels rather than the other way around. A recent analysis of the microbiome in individuals with autism found evidence to suggest that differences in composition were due to differences in diet, rather than causal effects of microbiome on autism predisposition (Yap et al., 2021). Such reverse effects have also been observed in studies of genetic confounding in MoBa, where mothers with higher genetic predisposition for ADHD were less likely to take vitamin supplements during pregnancy (Havdahl et al., 2022). As a result, supplementation interventions that ensure pregnant women with neurodevelopmental conditions are not experiencing nutritional deficiencies could still be beneficial for other maternal and offspring outcomes where evidence for a causal effect is more robust.

### Strengths and limitations

There were several strengths to our study design. First, we had a large sample of mother–father–child trios, with genotype data and detailed measures of child neurodevelopmental traits. To the best of our knowledge, this is the first implementation of a trio-MR design to test maternal effects of vitamin-D or DHA on offspring neurodevelopment. Previous studies have tried to control for shared genetic predisposition using only mother–child pairs however, adjusting for child PGS alone can lead to collider bias via father's genotype. We have also triangulated the findings with an MR study where we conducted several sensitivity analyses to assess possible bias from horizontal pleiotropy, and were able to bi-directionally assess reverse causation.

There were also several limitations to the current study, including the sample size of full trios. Power analyses suggested that we had 80% power to detect ORs < 1.105, so we were underpowered to detect smaller effects. However, such small maternal effects are unlikely to have a meaningful impact on neurodevelopmental outcomes. Second, we were not able to validate the genetic instruments for vitamin-D and DHA during pregnancy in the MoBa cohort. These genetic variants were identified in non-pregnant adults, and changes to metabolism during pregnancy might affect their suitability. However, we did validate the genetic instruments in a separate pregnancy cohort (ALSPAC), conducted a replication analysis using independent GWAS of diagnoses, and reran MR analyses using only canonical SNPs. These analyses also suggested that causal maternal effects were unlikely, increasing our confidence that the results are not limited to MoBa. Third, because SNP effects could not be regressed onto observed nutrient levels during pregnancy, maternal effects on offspring outcomes are not specific to pregnancy, but could also occur in the child's early life (e.g. through the food provided by the mother for the child). If effects of maternal genetic liability to nutrient levels act in the opposite direction during pregnancy than after birth, it could be the case that these effects cancel out, resulting in the lack of evidence observed here. However, such opposite effects seem unlikely, and we employed a paternal negative control design, in which we did not see evidence for effects of paternal nutrient levels on offspring outcomes via pathways occurring in early life. Fourth, the genetic variants for maternal circulating nutrients only proxy small variations and our analyses assume linear effects, meaning we might be underpowered to detect effects of extreme deficiencies. Finally, the MoBa sample is relatively homogeneous, and our analyses are restricted to individuals of European ancestry, hence, results might not be generalizable to other populations.

## Conclusion

Triangulating across complementary study designs, we did not find evidence to support causal maternal effects of vitamin-D and DHA on offspring neurodevelopmental outcomes, after accounting for shared genetic predisposition. There was evidence suggesting possible reverse causation, with genetic predisposition to autism associated with lower vitamin-D levels and genetic predisposition to ADHD associated with lower DHA levels. Previous observational associations might have been (at least in part) due to shared genetic predisposition to neurodevelopmental conditions, which also influences nutrient levels. Future studies exploring the role of intrauterine effects on offspring neurodevelopment should control for shared genetic predisposition and use designs robust to reverse causation.

## Supporting information

Wootton et al. supplementary material 1 Wootton et al. supplementary material

Wootton et al. supplementary material 2 Wootton et al. supplementary material

## Supplementary material

The supplementary material for this article can be found at https://doi.org/10.1017/S0033291724001466
