# Supplementary material for: Testing maternal effects of vitamin-D and omega-3 levels on offspring neurodevelopmental traits in the Norwegian Mother, Father and Child Cohort Study
Source: Psychol Med. 2024 Sep 9;54(12):3323–33. doi: 10.1017/S0033291724001466 (PMC11496238; doi:10.1017/S0033291724001466)
Supplement: Wootton et al. supplementary material 1 — Wootton et al. supplementary material [file S0033291724001466sup001.docx]

Supplementary Materials - Full Sample

Table of Contents

[Supplementary Tables 3](#_Toc158375845)

[Table S1. Number of genotyped individuals with neurodevelopmental trait measures in the MoBa cohort 3](#_Toc158375846)

[Table S2. Mendelian randomisation results for intrauterine Vitamin D/DHA on neurodevelopmental outcomes in the MoBa cohort 4](#_Toc158375847)

[Table S3. Tests of instrument strength and regression dilution I-squared for MR analyses in the MoBa cohort 10](#_Toc158375848)

[Table S4. Cochran’s Q test of heterogeneity for MR analyses in the MoBa cohort 11](#_Toc158375849)

[Table S5. MR Egger intercept test for MR analyses using maternal genotype in the MoBa cohort 12](#_Toc158375850)

[Table S6. Mendelian randomisation results for Vitamin D/DHA on neurodevelopmental outcomes in the MoBa cohort (using child genotypes) 14](#_Toc158375851)

[Table S7. Bi-directional MR Results for Vitamin D/DHA and diagnoses of autism and ADHD 24](#_Toc158375852)

[Table S8. Tests of instrument strength and regression dilution for the replication MR using PGC GWAS 25](#_Toc158375853)

[Table S9. Cochran’s Q test of heterogeneity for replication MR using PGC GWAS 26](#_Toc158375854)

[Table S10. MR Egger Test for replication MR using PGC GWAS 27](#_Toc158375855)

[Table S11. Maternal PGS adjusted for child/father PGS 28](#_Toc158375856)

[Table S12. Maternal DHA PGS adjusted for child/father PGS and stratified by child sex 30](#_Toc158375857)

[Table S13. Maternal Vitamin D PGS adjusted for child/father PGS and stratified by child sex 31](#_Toc158375858)

[Table S14. Child PGS adjusted for maternal and paternal PGS 32](#_Toc158375859)

[Table S15. Paternal negative control Analysis - Paternal PGS adjusted for child and maternal PGS 34](#_Toc158375860)

[Supplementary Figures 36](#_Toc158375861)

[Supplementary Figure S1. Leave-one-out Analysis for IVW Estimates of Autism Genetic Liability on Vitamin D 36](#_Toc158375862)

[Supplementary Figure S2. Leave-one-out Analysis for IVW Estimates of ADHD Genetic Liability on DHA levels 37](#_Toc158375863)

[Supplementary Figure S3. Mother PGS sex split DHA Page 38](#_Toc158375864)

[Supplementary Figure S4. Mother PGS sex split Vitamin D Page 39](#_Toc158375865)

[Supplementary Figure S5. Child PGS DHA Page 40](#_Toc158375866)

[Supplementary Figure S6. Child PGS Vitamin D Page 41](#_Toc158375867)

[Supplementary Figure S7. Paternal PGS negative control DHA Page 42](#_Toc158375868)

[Supplementary Figure S8. Paternal PGS negative control Vitamin D Page 43](#_Toc158375869)

[Supplementary Figure S9. Mendelian randomisation using canonical SNPs for DHA 44](#_Toc158375870)

[Supplementary Figure S10. Mendelian randomisation using canonical SNPs for Vitamin D 45](#_Toc158375871)

[Supplementary Figure S11. Overview of Analyses Conducted 46](#_Toc158375872)

## Supplementary Tables

### Table S1. Number of genotyped individuals with neurodevelopmental trait measures in the MoBa cohort

|  | **Domain** | **Age (Years)** | **Mother N** | **Father N** | **Child N** | **Trio N** |
| --- | --- | --- | --- | --- | --- | --- |
|  | Language | 3 | 23,713 | 17,990 | 26,646 | 16,298 |
|  | Language | 5 | 16,796 | 13,277 | 18,872 | 12,078 |
|  | Motor | 3 | 23,641 | 17,941 | 26,564 | 16,255 |
|  | Motor | 5 | 16,838 | 13,315 | 18,914 | 12,113 |
| ADHD | Hyperactivity | 3 | 23,310 | 17,697 | 26,207 | 16,032 |
| ADHD | Hyperactivity | 5 | 16,731 | 13,216 | 18,793 | 12,025 |
| ADHD | Hyperactivity | 8 | 17,724 | 13,761 | 19,971 | 12,451 |
| ADHD | Inattention | 3 | 23,318 | 17,697 | 26,192 | 16,042 |
| ADHD | Inattention | 5 | 16,754 | 13,246 | 18,819 | 12,053 |
| ADHD | Inattention | 8 | 17,728 | 13,765 | 19,975 | 12,455 |
| ADHD | Total | 3 | 23,374 | 17,753 | 26,266 | 16,090 |
| ADHD | Total | 5 | 16,841 | 13,311 | 18,918 | 12,112 |
| ADHD | Total | 8 | 17,730 | 13,766 | 19,977 | 12,456 |
| Autistic Traits | RRB | 3 | 23,652 | 17,952 | 26,582 | 16,266 |
| Autistic Traits | RRB | 8 | 17,128 | 13,308 | 19,299 | 12,041 |
| Autistic Traits | Social | 3 | 23,698 | 17,985 | 26,628 | 16,297 |
| Autistic Traits | Social | 8 | 17,636 | 13,690 | 19,872 | 12,382 |
| Autistic Traits | Total | 3 | 23,705 | 17,993 | 26,640 | 16,303 |
| Autistic Traits | Total | 8 | 17,647 | 13,700 | 19,884 | 12,391 |

###

### Table S2. Mendelian randomisation results for intrauterine Vitamin D/DHA on neurodevelopmental outcomes in the MoBa cohort

| **Nutrient** |  | **Domain** | **Age (Years)** | **Method** | **N SNP** | **Beta (95% CI)** | **P-value** |
| --- | --- | --- | --- | --- | --- | --- | --- |
| DHA | ADHD | Hyperactivity | 3 | Inverse variance weighted | 38 | -0.011 (-0.131, 0.109) | 0.85 |
| DHA | ADHD | Hyperactivity | 3 | MR Egger | 38 | 0.093 (-0.084, 0.271) | 0.31 |
| DHA | ADHD | Hyperactivity | 3 | Weighted median | 38 | 0.083 (-0.070, 0.236) | 0.29 |
| DHA | ADHD | Hyperactivity | 3 | Weighted mode | 38 | 0.030 (-0.114, 0.173) | 0.69 |
| DHA | ADHD | Hyperactivity | 5 | Inverse variance weighted | 38 | 0.021 (-0.077, 0.120) | 0.67 |
| DHA | ADHD | Hyperactivity | 5 | MR Egger | 38 | 0.014 (-0.131, 0.159) | 0.85 |
| DHA | ADHD | Hyperactivity | 5 | Weighted median | 38 | 0.025 (-0.096, 0.146) | 0.68 |
| DHA | ADHD | Hyperactivity | 5 | Weighted mode | 38 | 0.022 (-0.098, 0.143) | 0.72 |
| DHA | ADHD | Hyperactivity | 8 | Inverse variance weighted | 38 | -0.033 (-0.265, 0.200) | 0.78 |
| DHA | ADHD | Hyperactivity | 8 | MR Egger | 38 | 0.037 (-0.306, 0.380) | 0.83 |
| DHA | ADHD | Hyperactivity | 8 | Weighted median | 38 | 0.056 (-0.245, 0.358) | 0.71 |
| DHA | ADHD | Hyperactivity | 8 | Weighted mode | 38 | 0.034 (-0.252, 0.320) | 0.82 |
| DHA | ADHD | Inattention | 3 | Inverse variance weighted | 38 | 0.011 (-0.092, 0.114) | 0.83 |
| DHA | ADHD | Inattention | 3 | MR Egger | 38 | 0.024 (-0.129, 0.178) | 0.76 |
| DHA | ADHD | Inattention | 3 | Weighted median | 38 | 0.009 (-0.114, 0.132) | 0.88 |
| DHA | ADHD | Inattention | 3 | Weighted mode | 38 | 0.018 (-0.100, 0.137) | 0.76 |
| DHA | ADHD | Inattention | 5 | Inverse variance weighted | 38 | 0.026 (-0.029, 0.081) | 0.36 |
| DHA | ADHD | Inattention | 5 | MR Egger | 38 | 0.033 (-0.048, 0.115) | 0.43 |
| DHA | ADHD | Inattention | 5 | Weighted median | 38 | 0.009 (-0.062, 0.080) | 0.80 |
| DHA | ADHD | Inattention | 5 | Weighted mode | 38 | 0.010 (-0.058, 0.078) | 0.78 |
| DHA | ADHD | Inattention | 8 | Inverse variance weighted | 38 | 0.031 (-0.214, 0.275) | 0.81 |
| DHA | ADHD | Inattention | 8 | MR Egger | 38 | -0.001 (-0.361, 0.359) | 1.00 |
| DHA | ADHD | Inattention | 8 | Weighted median | 38 | 0.013 (-0.313, 0.338) | 0.94 |
| DHA | ADHD | Inattention | 8 | Weighted mode | 38 | 0.012 (-0.300, 0.324) | 0.94 |
| DHA | ADHD | Total | 3 | Inverse variance weighted | 38 | 0.0007 (-0.199, 0.200) | 0.99 |
| DHA | ADHD | Total | 3 | MR Egger | 38 | 0.131 (-0.162, 0.423) | 0.39 |
| DHA | ADHD | Total | 3 | Weighted median | 38 | 0.082 (-0.164, 0.328) | 0.51 |
| DHA | ADHD | Total | 3 | Weighted mode | 38 | 0.052 (-0.179, 0.283) | 0.66 |
| DHA | ADHD | Total | 5 | Inverse variance weighted | 38 | 0.043 (-0.091, 0.177) | 0.53 |
| DHA | ADHD | Total | 5 | MR Egger | 38 | 0.051 (-0.146, 0.249) | 0.61 |
| DHA | ADHD | Total | 5 | Weighted median | 38 | 0.037 (-0.131, 0.205) | 0.67 |
| DHA | ADHD | Total | 5 | Weighted mode | 38 | 0.029 (-0.133, 0.192) | 0.73 |
| DHA | ADHD | Total | 8 | Inverse variance weighted | 38 | -0.002 (-0.429, 0.426) | 0.99 |
| DHA | ADHD | Total | 8 | MR Egger | 38 | 0.032 (-0.598, 0.661) | 0.92 |
| DHA | ADHD | Total | 8 | Weighted median | 38 | 0.065 (-0.483, 0.613) | 0.82 |
| DHA | ADHD | Total | 8 | Weighted mode | 38 | 0.051 (-0.486, 0.588) | 0.85 |
| DHA | Autistic Traits | RRB | 3 | Inverse variance weighted | 38 | -0.023 (-0.154, 0.108) | 0.73 |
| DHA | Autistic Traits | RRB | 3 | MR Egger | 38 | 0.058 (-0.136, 0.251) | 0.56 |
| DHA | Autistic Traits | RRB | 3 | Weighted median | 38 | 0.048 (-0.123, 0.219) | 0.58 |
| DHA | Autistic Traits | RRB | 3 | Weighted mode | 38 | 0.006 (-0.162, 0.173) | 0.95 |
| DHA | Autistic Traits | RRB | 8 | Inverse variance weighted | 38 | -0.020 (-0.090, 0.050) | 0.57 |
| DHA | Autistic Traits | RRB | 8 | MR Egger | 38 | -0.012 (-0.115, 0.092) | 0.83 |
| DHA | Autistic Traits | RRB | 8 | Weighted median | 38 | -0.025 (-0.117, 0.067) | 0.60 |
| DHA | Autistic Traits | RRB | 8 | Weighted mode | 38 | -0.016 (-0.095, 0.062) | 0.69 |
| DHA | Autistic Traits | Social | 3 | Inverse variance weighted | 38 | 0.033 (-0.060, 0.125) | 0.49 |
| DHA | Autistic Traits | Social | 3 | MR Egger | 38 | 0.038 (-0.099, 0.175) | 0.59 |
| DHA | Autistic Traits | Social | 3 | Weighted median | 38 | 0.081 (-0.041, 0.203) | 0.19 |
| DHA | Autistic Traits | Social | 3 | Weighted mode | 38 | 0.063 (-0.049, 0.174) | 0.28 |
| DHA | Autistic Traits | Social | 8 | Inverse variance weighted | 38 | 0.091 (-0.057, 0.239) | 0.23 |
| DHA | Autistic Traits | Social | 8 | MR Egger | 38 | 0.185 (-0.033, 0.403) | 0.11 |
| DHA | Autistic Traits | Social | 8 | Weighted median | 38 | 0.130 (-0.067, 0.327) | 0.20 |
| DHA | Autistic Traits | Social | 8 | Weighted mode | 38 | 0.136 (-0.037, 0.309) | 0.13 |
| DHA | Autistic Traits | Total | 3 | Inverse variance weighted | 38 | 0.008 (-0.166, 0.181) | 0.93 |
| DHA | Autistic Traits | Total | 3 | MR Egger | 38 | 0.096 (-0.161, 0.352) | 0.47 |
| DHA | Autistic Traits | Total | 3 | Weighted median | 38 | 0.123 (-0.096, 0.341) | 0.27 |
| DHA | Autistic Traits | Total | 3 | Weighted mode | 38 | 0.074 (-0.141, 0.288) | 0.50 |
| DHA | Autistic Traits | Total | 8 | Inverse variance weighted | 38 | 0.059 (-0.117, 0.235) | 0.51 |
| DHA | Autistic Traits | Total | 8 | MR Egger | 38 | 0.156 (-0.104, 0.415) | 0.25 |
| DHA | Autistic Traits | Total | 8 | Weighted median | 38 | 0.121 (-0.109, 0.351) | 0.30 |
| DHA | Autistic Traits | Total | 8 | Weighted mode | 38 | 0.118 (-0.090, 0.326) | 0.27 |
| DHA | Language | Total | 3 | Inverse variance weighted | 38 | 0.009 (-0.058, 0.076) | 0.80 |
| DHA | Language | Total | 3 | MR Egger | 38 | 0.002 (-0.098, 0.102) | 0.97 |
| DHA | Language | Total | 3 | Weighted median | 38 | 0.027 (-0.048, 0.101) | 0.48 |
| DHA | Language | Total | 3 | Weighted mode | 38 | 0.023 (-0.048, 0.095) | 0.53 |
| DHA | Language | Total | 5 | Inverse variance weighted | 38 | 0.052 (-0.028, 0.132) | 0.21 |
| DHA | Language | Total | 5 | MR Egger | 38 | 0.057 (-0.062, 0.177) | 0.35 |
| DHA | Language | Total | 5 | Weighted median | 38 | 0.069 (-0.034, 0.173) | 0.19 |
| DHA | Language | Total | 5 | Weighted mode | 38 | 0.074 (-0.029, 0.177) | 0.17 |
| DHA | Motor | Total | 3 | Inverse variance weighted | 38 | -0.034 (-0.102, 0.034) | 0.32 |
| DHA | Motor | Total | 3 | MR Egger | 38 | -0.040 (-0.141, 0.060) | 0.44 |
| DHA | Motor | Total | 3 | Weighted median | 38 | -0.019 (-0.106, 0.068) | 0.66 |
| DHA | Motor | Total | 3 | Weighted mode | 38 | -0.022 (-0.108, 0.063) | 0.61 |
| DHA | Motor | Total | 5 | Inverse variance weighted | 38 | 0.029 (-0.066, 0.125) | 0.55 |
| DHA | Motor | Total | 5 | MR Egger | 38 | -0.064 (-0.201, 0.072) | 0.36 |
| DHA | Motor | Total | 5 | Weighted median | 38 | -0.010 (-0.121, 0.100) | 0.86 |
| DHA | Motor | Total | 5 | Weighted mode | 38 | 0.024 (-0.077, 0.124) | 0.65 |
| Vitamin D | ADHD | Hyperactivity | 3 | Inverse variance weighted | 56 | -0.005 (-0.016, 0.006) | 0.40 |
| Vitamin D | ADHD | Hyperactivity | 3 | MR Egger | 56 | 0.002 (-0.029, 0.033) | 0.90 |
| Vitamin D | ADHD | Hyperactivity | 3 | Weighted median | 56 | -0.005 (-0.028, 0.018) | 0.69 |
| Vitamin D | ADHD | Hyperactivity | 3 | Weighted mode | 56 | 0.003 (-0.028, 0.035) | 0.85 |
| Vitamin D | ADHD | Hyperactivity | 5 | Inverse variance weighted | 56 | -0.015 (-0.024, -0.006) | 0.001 |
| Vitamin D | ADHD | Hyperactivity | 5 | MR Egger | 56 | -0.013 (-0.038, 0.013) | 0.34 |
| Vitamin D | ADHD | Hyperactivity | 5 | Weighted median | 56 | -0.018 (-0.038, 0.002) | 0.07 |
| Vitamin D | ADHD | Hyperactivity | 5 | Weighted mode | 56 | -0.015 (-0.045, 0.015) | 0.32 |
| Vitamin D | ADHD | Hyperactivity | 8 | Inverse variance weighted | 56 | 0.012 (-0.009, 0.034) | 0.26 |
| Vitamin D | ADHD | Hyperactivity | 8 | MR Egger | 56 | -0.005 (-0.065, 0.056) | 0.88 |
| Vitamin D | ADHD | Hyperactivity | 8 | Weighted median | 56 | 0.026 (-0.021, 0.073) | 0.28 |
| Vitamin D | ADHD | Hyperactivity | 8 | Weighted mode | 56 | 0.042 (-0.019, 0.104) | 0.18 |
| Vitamin D | ADHD | Inattention | 3 | Inverse variance weighted | 56 | -0.003 (-0.013, 0.006) | 0.52 |
| Vitamin D | ADHD | Inattention | 3 | MR Egger | 56 | 0.007 (-0.020, 0.034) | 0.61 |
| Vitamin D | ADHD | Inattention | 3 | Weighted median | 56 | -0.002 (-0.022, 0.017) | 0.82 |
| Vitamin D | ADHD | Inattention | 3 | Weighted mode | 56 | -0.004 (-0.031, 0.023) | 0.76 |
| Vitamin D | ADHD | Inattention | 5 | Inverse variance weighted | 56 | -0.004 (-0.009, 0.001) | 0.16 |
| Vitamin D | ADHD | Inattention | 5 | MR Egger | 56 | -0.005 (-0.020, 0.009) | 0.47 |
| Vitamin D | ADHD | Inattention | 5 | Weighted median | 56 | -0.002 (-0.014, 0.009) | 0.66 |
| Vitamin D | ADHD | Inattention | 5 | Weighted mode | 56 | 0.0007 (-0.015, 0.017) | 0.93 |
| Vitamin D | ADHD | Inattention | 8 | Inverse variance weighted | 56 | -0.0001 (-0.026, 0.026) | 0.99 |
| Vitamin D | ADHD | Inattention | 8 | MR Egger | 56 | -0.028 (-0.101, 0.046) | 0.46 |
| Vitamin D | ADHD | Inattention | 8 | Weighted median | 56 | 0.021 (-0.026, 0.067) | 0.38 |
| Vitamin D | ADHD | Inattention | 8 | Weighted mode | 56 | 0.026 (-0.032, 0.085) | 0.38 |
| Vitamin D | ADHD | Total | 3 | Inverse variance weighted | 56 | -0.007 (-0.025, 0.011) | 0.43 |
| Vitamin D | ADHD | Total | 3 | MR Egger | 56 | 0.009 (-0.041, 0.058) | 0.73 |
| Vitamin D | ADHD | Total | 3 | Weighted median | 56 | -0.006 (-0.045, 0.033) | 0.76 |
| Vitamin D | ADHD | Total | 3 | Weighted mode | 56 | 0.015 (-0.037, 0.067) | 0.57 |
| Vitamin D | ADHD | Total | 5 | Inverse variance weighted | 56 | -0.018 (-0.030, -0.006) | 0.004 |
| Vitamin D | ADHD | Total | 5 | MR Egger | 56 | -0.017 (-0.052, 0.018) | 0.34 |
| Vitamin D | ADHD | Total | 5 | Weighted median | 56 | -0.018 (-0.045, 0.009) | 0.19 |
| Vitamin D | ADHD | Total | 5 | Weighted mode | 56 | -0.031 (-0.065, 0.003) | 0.08 |
| Vitamin D | ADHD | Total | 8 | Inverse variance weighted | 56 | 0.012 (-0.031, 0.056) | 0.58 |
| Vitamin D | ADHD | Total | 8 | MR Egger | 56 | -0.035 (-0.159, 0.088) | 0.58 |
| Vitamin D | ADHD | Total | 8 | Weighted median | 56 | 0.038 (-0.048, 0.125) | 0.39 |
| Vitamin D | ADHD | Total | 8 | Weighted mode | 56 | 0.058 (-0.037, 0.154) | 0.24 |
| Vitamin D | Autistic Traits | RRB | 3 | Inverse variance weighted | 56 | -0.005 (-0.018, 0.007) | 0.42 |
| Vitamin D | Autistic Traits | RRB | 3 | MR Egger | 56 | -0.009 (-0.045, 0.028) | 0.65 |
| Vitamin D | Autistic Traits | RRB | 3 | Weighted median | 56 | -0.004 (-0.030, 0.023) | 0.79 |
| Vitamin D | Autistic Traits | RRB | 3 | Weighted mode | 56 | 0.004 (-0.026, 0.034) | 0.78 |
| Vitamin D | Autistic Traits | RRB | 8 | Inverse variance weighted | 56 | 0.003 (-0.003, 0.009) | 0.36 |
| Vitamin D | Autistic Traits | RRB | 8 | MR Egger | 56 | -0.004 (-0.022, 0.014) | 0.69 |
| Vitamin D | Autistic Traits | RRB | 8 | Weighted median | 56 | 0.008 (-0.005, 0.022) | 0.22 |
| Vitamin D | Autistic Traits | RRB | 8 | Weighted mode | 56 | 0.010 (-0.006, 0.027) | 0.22 |
| Vitamin D | Autistic Traits | Social | 3 | Inverse variance weighted | 56 | 0.004 (-0.005, 0.012) | 0.37 |
| Vitamin D | Autistic Traits | Social | 3 | MR Egger | 56 | -0.006 (-0.030, 0.019) | 0.65 |
| Vitamin D | Autistic Traits | Social | 3 | Weighted median | 56 | 0.004 (-0.014, 0.022) | 0.65 |
| Vitamin D | Autistic Traits | Social | 3 | Weighted mode | 56 | 0.004 (-0.017, 0.025) | 0.70 |
| Vitamin D | Autistic Traits | Social | 8 | Inverse variance weighted | 56 | 0.002 (-0.011, 0.016) | 0.74 |
| Vitamin D | Autistic Traits | Social | 8 | MR Egger | 56 | 0.010 (-0.028, 0.048) | 0.61 |
| Vitamin D | Autistic Traits | Social | 8 | Weighted median | 56 | 0.002 (-0.028, 0.032) | 0.90 |
| Vitamin D | Autistic Traits | Social | 8 | Weighted mode | 56 | 0.0008 (-0.037, 0.038) | 0.97 |
| Vitamin D | Autistic Traits | Total | 3 | Inverse variance weighted | 56 | -0.002 (-0.018, 0.014) | 0.80 |
| Vitamin D | Autistic Traits | Total | 3 | MR Egger | 56 | -0.012 (-0.059, 0.035) | 0.62 |
| Vitamin D | Autistic Traits | Total | 3 | Weighted median | 56 | -0.002 (-0.037, 0.032) | 0.90 |
| Vitamin D | Autistic Traits | Total | 3 | Weighted mode | 56 | -0.002 (-0.041, 0.038) | 0.93 |
| Vitamin D | Autistic Traits | Total | 8 | Inverse variance weighted | 56 | 0.005 (-0.011, 0.021) | 0.52 |
| Vitamin D | Autistic Traits | Total | 8 | MR Egger | 56 | 0.005 (-0.040, 0.051) | 0.82 |
| Vitamin D | Autistic Traits | Total | 8 | Weighted median | 56 | 0.008 (-0.025, 0.041) | 0.65 |
| Vitamin D | Autistic Traits | Total | 8 | Weighted mode | 56 | 0.008 (-0.035, 0.050) | 0.72 |
| Vitamin D | Language | Total | 3 | Inverse variance weighted | 56 | 0.005 ( 0.00005, 0.011) | 0.05 |
| Vitamin D | Language | Total | 3 | MR Egger | 56 | 0.012 (-0.003, 0.027) | 0.12 |
| Vitamin D | Language | Total | 3 | Weighted median | 56 | 0.006 (-0.006, 0.018) | 0.30 |
| Vitamin D | Language | Total | 3 | Weighted mode | 56 | 0.007 (-0.006, 0.020) | 0.30 |
| Vitamin D | Language | Total | 5 | Inverse variance weighted | 56 | 0.003 (-0.005, 0.010) | 0.48 |
| Vitamin D | Language | Total | 5 | MR Egger | 56 | 0.006 (-0.015, 0.026) | 0.58 |
| Vitamin D | Language | Total | 5 | Weighted median | 56 | 0.0003 (-0.015, 0.016) | 0.97 |
| Vitamin D | Language | Total | 5 | Weighted mode | 56 | -0.0006 (-0.021, 0.019) | 0.95 |
| Vitamin D | Motor | Total | 3 | Inverse variance weighted | 56 | 0.005 (-0.001, 0.011) | 0.12 |
| Vitamin D | Motor | Total | 3 | MR Egger | 56 | 0.0005 (-0.017, 0.019) | 0.95 |
| Vitamin D | Motor | Total | 3 | Weighted median | 56 | 0.007 (-0.006, 0.021) | 0.28 |
| Vitamin D | Motor | Total | 3 | Weighted mode | 56 | 0.009 (-0.009, 0.026) | 0.33 |
| Vitamin D | Motor | Total | 5 | Inverse variance weighted | 56 | -0.003 (-0.011, 0.005) | 0.41 |
| Vitamin D | Motor | Total | 5 | MR Egger | 56 | -0.005 (-0.028, 0.017) | 0.64 |
| Vitamin D | Motor | Total | 5 | Weighted median | 56 | 0.003 (-0.013, 0.019) | 0.69 |
| Vitamin D | Motor | Total | 5 | Weighted mode | 56 | 0.011 (-0.010, 0.032) | 0.31 |
| Vitamin D (Revez et al. 2020) | ADHD | Hyperactivity | 3 | Inverse variance weighted | 91 | -0.149 (-0.327, 0.029) | 0.10 |
| Vitamin D (Revez et al. 2020) | ADHD | Hyperactivity | 3 | MR Egger | 91 | -0.259 (-0.529, 0.012) | 0.06 |
| Vitamin D (Revez et al. 2020) | ADHD | Hyperactivity | 3 | Weighted median | 91 | -0.181 (-0.463, 0.101) | 0.21 |
| Vitamin D (Revez et al. 2020) | ADHD | Hyperactivity | 3 | Weighted mode | 91 | -0.170 (-0.438, 0.099) | 0.22 |
| Vitamin D (Revez et al. 2020) | ADHD | Hyperactivity | 5 | Inverse variance weighted | 91 | -0.089 (-0.252, 0.073) | 0.28 |
| Vitamin D (Revez et al. 2020) | ADHD | Hyperactivity | 5 | MR Egger | 91 | -0.068 (-0.316, 0.180) | 0.59 |
| Vitamin D (Revez et al. 2020) | ADHD | Hyperactivity | 5 | Weighted median | 91 | -0.043 (-0.280, 0.194) | 0.72 |
| Vitamin D (Revez et al. 2020) | ADHD | Hyperactivity | 5 | Weighted mode | 91 | -0.050 (-0.237, 0.136) | 0.60 |
| Vitamin D (Revez et al. 2020) | ADHD | Hyperactivity | 8 | Inverse variance weighted | 91 | -0.037 (-0.382, 0.309) | 0.84 |
| Vitamin D (Revez et al. 2020) | ADHD | Hyperactivity | 8 | MR Egger | 91 | -0.035 (-0.559, 0.488) | 0.89 |
| Vitamin D (Revez et al. 2020) | ADHD | Hyperactivity | 8 | Weighted median | 91 | -0.375 (-0.906, 0.156) | 0.17 |
| Vitamin D (Revez et al. 2020) | ADHD | Hyperactivity | 8 | Weighted mode | 91 | -0.157 (-0.632, 0.318) | 0.52 |
| Vitamin D (Revez et al. 2020) | ADHD | Inattention | 3 | Inverse variance weighted | 91 | -0.183 (-0.325, -0.040) | 0.01 |
| Vitamin D (Revez et al. 2020) | ADHD | Inattention | 3 | MR Egger | 91 | -0.157 (-0.373, 0.060) | 0.16 |
| Vitamin D (Revez et al. 2020) | ADHD | Inattention | 3 | Weighted median | 91 | -0.233 (-0.470, 0.005) | 0.05 |
| Vitamin D (Revez et al. 2020) | ADHD | Inattention | 3 | Weighted mode | 91 | -0.210 (-0.407, -0.013) | 0.04 |
| Vitamin D (Revez et al. 2020) | ADHD | Inattention | 5 | Inverse variance weighted | 91 | -0.097 (-0.179, -0.015) | 0.02 |
| Vitamin D (Revez et al. 2020) | ADHD | Inattention | 5 | MR Egger | 91 | -0.114 (-0.239, 0.011) | 0.08 |
| Vitamin D (Revez et al. 2020) | ADHD | Inattention | 5 | Weighted median | 91 | -0.137 (-0.269, -0.006) | 0.04 |
| Vitamin D (Revez et al. 2020) | ADHD | Inattention | 5 | Weighted mode | 91 | -0.093 (-0.200, 0.013) | 0.09 |
| Vitamin D (Revez et al. 2020) | ADHD | Inattention | 8 | Inverse variance weighted | 91 | -0.207 (-0.599, 0.184) | 0.30 |
| Vitamin D (Revez et al. 2020) | ADHD | Inattention | 8 | MR Egger | 91 | -0.073 (-0.670, 0.523) | 0.81 |
| Vitamin D (Revez et al. 2020) | ADHD | Inattention | 8 | Weighted median | 91 | -0.664 (-1.238, -0.089) | 0.02 |
| Vitamin D (Revez et al. 2020) | ADHD | Inattention | 8 | Weighted mode | 91 | -0.457 (-0.963, 0.050) | 0.08 |
| Vitamin D (Revez et al. 2020) | ADHD | Total | 3 | Inverse variance weighted | 91 | -0.312 (-0.597, -0.028) | 0.03 |
| Vitamin D (Revez et al. 2020) | ADHD | Total | 3 | MR Egger | 91 | -0.377 (-0.809, 0.055) | 0.09 |
| Vitamin D (Revez et al. 2020) | ADHD | Total | 3 | Weighted median | 91 | -0.394 (-0.840, 0.052) | 0.08 |
| Vitamin D (Revez et al. 2020) | ADHD | Total | 3 | Weighted mode | 91 | -0.353 (-0.771, 0.065) | 0.10 |
| Vitamin D (Revez et al. 2020) | ADHD | Total | 5 | Inverse variance weighted | 91 | -0.181 (-0.392, 0.031) | 0.09 |
| Vitamin D (Revez et al. 2020) | ADHD | Total | 5 | MR Egger | 91 | -0.185 (-0.509, 0.138) | 0.26 |
| Vitamin D (Revez et al. 2020) | ADHD | Total | 5 | Weighted median | 91 | -0.189 (-0.519, 0.141) | 0.26 |
| Vitamin D (Revez et al. 2020) | ADHD | Total | 5 | Weighted mode | 91 | -0.145 (-0.406, 0.116) | 0.28 |
| Vitamin D (Revez et al. 2020) | ADHD | Total | 8 | Inverse variance weighted | 91 | -0.249 (-0.887, 0.388) | 0.44 |
| Vitamin D (Revez et al. 2020) | ADHD | Total | 8 | MR Egger | 91 | -0.132 (-1.104, 0.840) | 0.79 |
| Vitamin D (Revez et al. 2020) | ADHD | Total | 8 | Weighted median | 91 | -1.117 (-2.123, -0.111) | 0.03 |
| Vitamin D (Revez et al. 2020) | ADHD | Total | 8 | Weighted mode | 91 | -0.432 (-1.283, 0.420) | 0.32 |
| Vitamin D (Revez et al. 2020) | Autistic Traits | RRB | 3 | Inverse variance weighted | 91 | -0.153 (-0.347, 0.041) | 0.12 |
| Vitamin D (Revez et al. 2020) | Autistic Traits | RRB | 3 | MR Egger | 91 | -0.279 (-0.574, 0.016) | 0.07 |
| Vitamin D (Revez et al. 2020) | Autistic Traits | RRB | 3 | Weighted median | 91 | -0.149 (-0.461, 0.162) | 0.35 |
| Vitamin D (Revez et al. 2020) | Autistic Traits | RRB | 3 | Weighted mode | 91 | -0.171 (-0.444, 0.102) | 0.22 |
| Vitamin D (Revez et al. 2020) | Autistic Traits | RRB | 8 | Inverse variance weighted | 91 | -0.051 (-0.161, 0.059) | 0.36 |
| Vitamin D (Revez et al. 2020) | Autistic Traits | RRB | 8 | MR Egger | 91 | -0.015 (-0.182, 0.153) | 0.86 |
| Vitamin D (Revez et al. 2020) | Autistic Traits | RRB | 8 | Weighted median | 91 | -0.086 (-0.261, 0.088) | 0.33 |
| Vitamin D (Revez et al. 2020) | Autistic Traits | RRB | 8 | Weighted mode | 91 | -0.047 (-0.199, 0.105) | 0.55 |
| Vitamin D (Revez et al. 2020) | Autistic Traits | Social | 3 | Inverse variance weighted | 91 | 0.032 (-0.105, 0.170) | 0.65 |
| Vitamin D (Revez et al. 2020) | Autistic Traits | Social | 3 | MR Egger | 91 | 0.131 (-0.078, 0.340) | 0.22 |
| Vitamin D (Revez et al. 2020) | Autistic Traits | Social | 3 | Weighted median | 91 | 0.013 (-0.202, 0.229) | 0.90 |
| Vitamin D (Revez et al. 2020) | Autistic Traits | Social | 3 | Weighted mode | 91 | 0.059 (-0.124, 0.242) | 0.53 |
| Vitamin D (Revez et al. 2020) | Autistic Traits | Social | 8 | Inverse variance weighted | 91 | 0.137 (-0.087, 0.360) | 0.23 |
| Vitamin D (Revez et al. 2020) | Autistic Traits | Social | 8 | MR Egger | 91 | 0.151 (-0.190, 0.491) | 0.39 |
| Vitamin D (Revez et al. 2020) | Autistic Traits | Social | 8 | Weighted median | 91 | 0.223 (-0.122, 0.567) | 0.21 |
| Vitamin D (Revez et al. 2020) | Autistic Traits | Social | 8 | Weighted mode | 91 | 0.167 (-0.140, 0.473) | 0.29 |
| Vitamin D (Revez et al. 2020) | Autistic Traits | Total | 3 | Inverse variance weighted | 91 | -0.166 (-0.423, 0.092) | 0.21 |
| Vitamin D (Revez et al. 2020) | Autistic Traits | Total | 3 | MR Egger | 91 | -0.178 (-0.569, 0.213) | 0.37 |
| Vitamin D (Revez et al. 2020) | Autistic Traits | Total | 3 | Weighted median | 91 | -0.216 (-0.616, 0.184) | 0.29 |
| Vitamin D (Revez et al. 2020) | Autistic Traits | Total | 3 | Weighted mode | 91 | -0.168 (-0.507, 0.170) | 0.33 |
| Vitamin D (Revez et al. 2020) | Autistic Traits | Total | 8 | Inverse variance weighted | 91 | 0.107 (-0.154, 0.367) | 0.42 |
| Vitamin D (Revez et al. 2020) | Autistic Traits | Total | 8 | MR Egger | 91 | 0.193 (-0.203, 0.589) | 0.34 |
| Vitamin D (Revez et al. 2020) | Autistic Traits | Total | 8 | Weighted median | 91 | 0.179 (-0.234, 0.591) | 0.40 |
| Vitamin D (Revez et al. 2020) | Autistic Traits | Total | 8 | Weighted mode | 91 | 0.153 (-0.184, 0.489) | 0.38 |
| Vitamin D (Revez et al. 2020) | Language | Total | 3 | Inverse variance weighted | 91 | -0.008 (-0.097, 0.081) | 0.85 |
| Vitamin D (Revez et al. 2020) | Language | Total | 3 | MR Egger | 91 | -0.008 (-0.144, 0.127) | 0.90 |
| Vitamin D (Revez et al. 2020) | Language | Total | 3 | Weighted median | 91 | -0.058 (-0.190, 0.075) | 0.40 |
| Vitamin D (Revez et al. 2020) | Language | Total | 3 | Weighted mode | 91 | -0.011 (-0.113, 0.090) | 0.83 |
| Vitamin D (Revez et al. 2020) | Language | Total | 5 | Inverse variance weighted | 91 | -0.019 (-0.148, 0.110) | 0.77 |
| Vitamin D (Revez et al. 2020) | Language | Total | 5 | MR Egger | 91 | 0.052 (-0.145, 0.248) | 0.61 |
| Vitamin D (Revez et al. 2020) | Language | Total | 5 | Weighted median | 91 | -0.021 (-0.210, 0.168) | 0.83 |
| Vitamin D (Revez et al. 2020) | Language | Total | 5 | Weighted mode | 91 | 0.002 (-0.161, 0.166) | 0.98 |
| Vitamin D (Revez et al. 2020) | Motor | Total | 3 | Inverse variance weighted | 91 | 0.025 (-0.076, 0.125) | 0.63 |
| Vitamin D (Revez et al. 2020) | Motor | Total | 3 | MR Egger | 91 | -0.011 (-0.164, 0.142) | 0.89 |
| Vitamin D (Revez et al. 2020) | Motor | Total | 3 | Weighted median | 91 | -0.044 (-0.209, 0.122) | 0.61 |
| Vitamin D (Revez et al. 2020) | Motor | Total | 3 | Weighted mode | 91 | -0.035 (-0.176, 0.107) | 0.63 |
| Vitamin D (Revez et al. 2020) | Motor | Total | 5 | Inverse variance weighted | 91 | -0.083 (-0.223, 0.058) | 0.25 |
| Vitamin D (Revez et al. 2020) | Motor | Total | 5 | MR Egger | 91 | -0.125 (-0.339, 0.089) | 0.25 |
| Vitamin D (Revez et al. 2020) | Motor | Total | 5 | Weighted median | 91 | -0.147 (-0.335, 0.041) | 0.13 |
| Vitamin D (Revez et al. 2020) | Motor | Total | 5 | Weighted mode | 91 | -0.114 (-0.293, 0.065) | 0.21 |

###

### Table S3. Tests of instrument strength and regression dilution I-squared for MR analyses in the MoBa cohort

|  | | | **DHA** | | | **Vitamin D (Manousaki et al.)** | | | **Vitamin D (Revez et al.)** | | |
| --- | --- | --- | --- | --- | --- | --- | --- | --- | --- | --- | --- |
|  | **Domain** | **Age (Years)** | **mF** | **Isq Unweighted** | **Isq Weighted** | **mF** | **Isq Unweighted** | **Isq Weighted** | **mF** | **Isq Unweighted** | **Isq Weighted** |
|  | Language | 3 | 196.97 | 0.99 | 0.99 | 1017.98 | 0.99 | 0.99 | 148.28 | 0.98 | 0.98 |
|  | Language | 5 | 196.97 | 0.99 | 0.99 | 1017.98 | 0.99 | 0.99 | 148.28 | 0.98 | 0.98 |
|  | Motor | 3 | 196.97 | 0.99 | 0.99 | 1017.98 | 0.99 | 0.99 | 148.28 | 0.98 | 0.98 |
|  | Motor | 5 | 196.97 | 0.99 | 0.99 | 1017.98 | 0.99 | 0.99 | 148.28 | 0.98 | 0.98 |
| ADHD | hyper | 3 | 196.97 | 0.99 | 0.99 | 1017.98 | 0.99 | 0.99 | 148.28 | 0.98 | 0.98 |
| ADHD | Hyperactivity | 5yss | 196.97 | 0.99 | 0.99 | 1017.98 | 0.99 | 0.99 | 148.28 | 0.98 | 0.98 |
| ADHD | Hyperactivity | 8 | 196.97 | 0.99 | 0.99 | 1017.98 | 0.99 | 0.99 | 148.28 | 0.98 | 0.98 |
| ADHD | Inattention | 3 | 196.97 | 0.99 | 0.99 | 1017.98 | 0.99 | 0.99 | 148.28 | 0.98 | 0.98 |
| ADHD | Inattention | 5yss | 196.97 | 0.99 | 0.99 | 1017.98 | 0.99 | 0.99 | 148.28 | 0.98 | 0.98 |
| ADHD | Inattention | 8 | 196.97 | 0.99 | 0.99 | 1017.98 | 0.99 | 0.99 | 148.28 | 0.98 | 0.98 |
| ADHD | Total | 3 | 196.97 | 0.99 | 0.99 | 1017.98 | 0.99 | 0.99 | 148.28 | 0.98 | 0.98 |
| ADHD | Total | 5 | 196.97 | 0.99 | 0.99 | 1017.98 | 0.99 | 0.99 | 148.28 | 0.98 | 0.98 |
| ADHD | Total | 8 | 196.97 | 0.99 | 0.99 | 1017.98 | 0.99 | 0.99 | 148.28 | 0.98 | 0.98 |
| Autistic Traits | RRB | 3 | 196.97 | 0.99 | 0.99 | 1017.98 | 0.99 | 0.99 | 148.28 | 0.98 | 0.98 |
| Autistic Traits | RRB | 8 | 196.97 | 0.99 | 0.99 | 1017.98 | 0.99 | 0.99 | 148.28 | 0.98 | 0.98 |
| Autistic Traits | Social | 3 | 196.97 | 0.99 | 0.99 | 1017.98 | 0.99 | 0.99 | 148.28 | 0.98 | 0.98 |
| Autistic Traits | Social | 8 | 196.97 | 0.99 | 0.99 | 1017.98 | 0.99 | 0.99 | 148.28 | 0.98 | 0.98 |
| Autistic Traits | Total | 3 | 196.97 | 0.99 | 0.99 | 1017.98 | 0.99 | 0.99 | 148.28 | 0.98 | 0.98 |
| Autistic Traits | Total | 8 | 196.97 | 0.99 | 0.99 | 1017.98 | 0.99 | 0.99 | 148.28 | 0.98 | 0.98 |

### Table S4. Cochran’s Q test of heterogeneity for MR analyses in the MoBa cohort

|  | | | **DHA** | | | **Vitamin D (Manousaki et al.)** | | | **Vitamin D (Revez et al.)** | | |
| --- | --- | --- | --- | --- | --- | --- | --- | --- | --- | --- | --- |
|  | **Domain** | **Age (Years)** | **Q** | **df** | **P-value** | **Q** | **df** | **P-value** | **Q** | **df** | **P-value** |
|  | Language | 3 | 47.99 | 37 | 0.11 | 53.20 | 55 | 0.54 | 94.18 | 90 | 0.36 |
|  | Language | 5 | 38.80 | 37 | 0.39 | 53.12 | 55 | 0.55 | 111.49 | 90 | 0.06 |
|  | Motor | 3 | 28.56 | 37 | 0.84 | 57.07 | 55 | 0.40 | 86.31 | 90 | 0.59 |
|  | Motor | 5 | 45.13 | 37 | 0.17 | 54.17 | 55 | 0.51 | 108.13 | 90 | 0.09 |
| ADHD | hyper | 3 | 35.31 | 37 | 0.55 | 47.80 | 55 | 0.74 | 70.39 | 90 | 0.94 |
| ADHD | Hyperactivity | 5yss | 27.06 | 37 | 0.88 | 55.33 | 55 | 0.46 | 111.60 | 90 | 0.06 |
| ADHD | Hyperactivity | 8 | 28.04 | 37 | 0.86 | 54.57 | 55 | 0.49 | 74.84 | 90 | 0.87 |
| ADHD | Inattention | 3 | 42.09 | 37 | 0.26 | 63.25 | 55 | 0.21 | 82.86 | 90 | 0.69 |
| ADHD | Inattention | 5yss | 19.45 | 37 | 0.99 | 53.91 | 55 | 0.52 | 90.50 | 90 | 0.47 |
| ADHD | Inattention | 8 | 36.02 | 37 | 0.51 | 73.65 | 55 | 0.05 | 105.42 | 90 | 0.13 |
| ADHD | Total | 3 | 39.81 | 37 | 0.35 | 54.47 | 55 | 0.49 | 71.53 | 90 | 0.92 |
| ADHD | Total | 5 | 21.76 | 37 | 0.98 | 48.67 | 55 | 0.71 | 102.22 | 90 | 0.18 |
| ADHD | Total | 8 | 33.26 | 37 | 0.65 | 68.34 | 55 | 0.11 | 91.16 | 90 | 0.45 |
| Autistic Traits | RRB | 3 | 36.00 | 37 | 0.52 | 61.61 | 55 | 0.25 | 77.58 | 90 | 0.82 |
| Autistic Traits | RRB | 8 | 28.89 | 37 | 0.83 | 45.78 | 55 | 0.81 | 100.69 | 90 | 0.21 |
| Autistic Traits | Social | 3 | 32.27 | 37 | 0.69 | 43.77 | 55 | 0.86 | 63.63 | 90 | 0.98 |
| Autistic Traits | Social | 8 | 34.19 | 37 | 0.60 | 54.33 | 55 | 0.50 | 92.82 | 90 | 0.40 |
| Autistic Traits | Total | 3 | 31.89 | 37 | 0.71 | 58.64 | 55 | 0.34 | 76.34 | 90 | 0.85 |
| Autistic Traits | Total | 8 | 33.36 | 37 | 0.64 | 41.92 | 55 | 0.90 | 88.37 | 90 | 0.53 |

###

### Table S5. MR Egger intercept test for MR analyses using maternal genotype in the MoBa cohort

|  |  | **Domain** | **Age (Years)** | **Intercept (95% CI)** | **P-value** |
| --- | --- | --- | --- | --- | --- |
| DHA |  | Language | 3 | 0.000700 (-0.007, 0.008) | 0.86 |
| DHA |  | Language | 5 | -0.000600 (-0.009, 0.008) | 0.90 |
| DHA |  | Motor | 3 | 0.000600 (-0.007, 0.008) | 0.87 |
| DHA |  | Motor | 5 | 0.009 (-0.0006, 0.019) | 0.08 |
| DHA | ADHD | hyper | 3 | -0.010 (-0.023, 0.003) | 0.12 |
| DHA | ADHD | Hyperactivity | 5yss | 0.000700 (-0.010, 0.011) | 0.89 |
| DHA | ADHD | Hyperactivity | 8 | -0.007 (-0.032, 0.018) | 0.59 |
| DHA | ADHD | Inattention | 3 | -0.001 (-0.012, 0.010) | 0.82 |
| DHA | ADHD | Inattention | 5yss | -0.000700 (-0.007, 0.005) | 0.81 |
| DHA | ADHD | Inattention | 8 | 0.003 (-0.023, 0.029) | 0.82 |
| DHA | ADHD | Total | 3 | -0.013 (-0.034, 0.008) | 0.24 |
| DHA | ADHD | Total | 5 | -0.000800 (-0.015, 0.013) | 0.91 |
| DHA | ADHD | Total | 8 | -0.003 (-0.049, 0.042) | 0.89 |
| DHA | Autistic Traits | RRB | 3 | -0.008 (-0.022, 0.006) | 0.28 |
| DHA | Autistic Traits | RRB | 8 | -0.000800 (-0.008, 0.007) | 0.83 |
| DHA | Autistic Traits | Social | 3 | -0.000500 (-0.010, 0.009) | 0.92 |
| DHA | Autistic Traits | Social | 8 | -0.009 (-0.025, 0.007) | 0.26 |
| DHA | Autistic Traits | Total | 3 | -0.009 (-0.027, 0.010) | 0.37 |
| DHA | Autistic Traits | Total | 8 | -0.010 (-0.028, 0.009) | 0.33 |
| Vitamin D (Manousaki et al.) |  | Language | 3 | -0.004 (-0.013, 0.005) | 0.35 |
| Vitamin D (Manousaki et al.) |  | Language | 5 | -0.002 (-0.014, 0.010) | 0.75 |
| Vitamin D (Manousaki et al.) |  | Motor | 3 | 0.003 (-0.008, 0.014) | 0.60 |
| Vitamin D (Manousaki et al.) |  | Motor | 5 | 0.001 (-0.012, 0.015) | 0.85 |
| Vitamin D (Manousaki et al.) | ADHD | hyper | 3 | -0.004 (-0.023, 0.014) | 0.66 |
| Vitamin D (Manousaki et al.) | ADHD | Hyperactivity | 5yss | -0.001 (-0.017, 0.014) | 0.87 |
| Vitamin D (Manousaki et al.) | ADHD | Hyperactivity | 8 | 0.011 (-0.025, 0.046) | 0.56 |
| Vitamin D (Manousaki et al.) | ADHD | Inattention | 3 | -0.006 (-0.022, 0.009) | 0.43 |
| Vitamin D (Manousaki et al.) | ADHD | Inattention | 5yss | 0.001 (-0.007, 0.010) | 0.81 |
| Vitamin D (Manousaki et al.) | ADHD | Inattention | 8 | 0.017 (-0.026, 0.061) | 0.44 |
| Vitamin D (Manousaki et al.) | ADHD | Total | 3 | -0.010 (-0.039, 0.020) | 0.51 |
| Vitamin D (Manousaki et al.) | ADHD | Total | 5 | -0.000800 (-0.021, 0.020) | 0.94 |
| Vitamin D (Manousaki et al.) | ADHD | Total | 8 | 0.030 (-0.043, 0.104) | 0.42 |
| Vitamin D (Manousaki et al.) | Autistic Traits | RRB | 3 | 0.002 (-0.019, 0.024) | 0.85 |
| Vitamin D (Manousaki et al.) | Autistic Traits | RRB | 8 | 0.004 (-0.007, 0.015) | 0.44 |
| Vitamin D (Manousaki et al.) | Autistic Traits | Social | 3 | 0.006 (-0.008, 0.020) | 0.42 |
| Vitamin D (Manousaki et al.) | Autistic Traits | Social | 8 | -0.005 (-0.028, 0.018) | 0.68 |
| Vitamin D (Manousaki et al.) | Autistic Traits | Total | 3 | 0.006 (-0.022, 0.034) | 0.67 |
| Vitamin D (Manousaki et al.) | Autistic Traits | Total | 8 | -0.000070 (-0.027, 0.027) | 1.00 |
| Vitamin D (Revez et al.) |  | Language | 3 | 0.000006 (-0.004, 0.004) | 1.00 |
| Vitamin D (Revez et al.) |  | Language | 5 | -0.003 (-0.008, 0.003) | 0.35 |
| Vitamin D (Revez et al.) |  | Motor | 3 | 0.001 (-0.003, 0.006) | 0.54 |
| Vitamin D (Revez et al.) |  | Motor | 5 | 0.002 (-0.005, 0.008) | 0.61 |
| Vitamin D (Revez et al.) | ADHD | hyper | 3 | 0.004 (-0.004, 0.012) | 0.29 |
| Vitamin D (Revez et al.) | ADHD | Hyperactivity | 5yss | -0.000800 (-0.008, 0.006) | 0.82 |
| Vitamin D (Revez et al.) | ADHD | Hyperactivity | 8 | -0.000040 (-0.015, 0.015) | 1.00 |
| Vitamin D (Revez et al.) | ADHD | Inattention | 3 | -0.001000 (-0.007, 0.005) | 0.76 |
| Vitamin D (Revez et al.) | ADHD | Inattention | 5yss | 0.000700 (-0.003, 0.004) | 0.72 |
| Vitamin D (Revez et al.) | ADHD | Inattention | 8 | -0.005 (-0.022, 0.012) | 0.56 |
| Vitamin D (Revez et al.) | ADHD | Total | 3 | 0.002 (-0.010, 0.015) | 0.70 |
| Vitamin D (Revez et al.) | ADHD | Total | 5 | 0.000200 (-0.009, 0.009) | 0.97 |
| Vitamin D (Revez et al.) | ADHD | Total | 8 | -0.005 (-0.032, 0.023) | 0.75 |
| Vitamin D (Revez et al.) | Autistic Traits | RRB | 3 | 0.005 (-0.004, 0.013) | 0.27 |
| Vitamin D (Revez et al.) | Autistic Traits | RRB | 8 | -0.001 (-0.006, 0.003) | 0.57 |
| Vitamin D (Revez et al.) | Autistic Traits | Social | 3 | -0.004 (-0.010, 0.002) | 0.22 |
| Vitamin D (Revez et al.) | Autistic Traits | Social | 8 | -0.000500 (-0.010, 0.009) | 0.91 |
| Vitamin D (Revez et al.) | Autistic Traits | Total | 3 | 0.000500 (-0.011, 0.012) | 0.93 |
| Vitamin D (Revez et al.) | Autistic Traits | Total | 8 | -0.003 (-0.015, 0.008) | 0.57 |

###

### Table S6. Mendelian randomisation results for Vitamin D/DHA on neurodevelopmental outcomes in the MoBa cohort (using child genotypes)

| **Nutrient** |  | **Domain** | **Age (Years)** | **Method** | **N SNP** | **Beta (95% CI)** | **P-value** |
| --- | --- | --- | --- | --- | --- | --- | --- |
| DHA |  | Language | 3 | Inverse variance weighted | 38 | -0.016 (-0.071, 0.039) | 0.57 |
| DHA |  | Language | 3 | MR Egger | 38 | -0.025 (-0.107, 0.056) | 0.54 |
| DHA |  | Language | 3 | Weighted median | 38 | -0.005 (-0.076, 0.067) | 0.90 |
| DHA |  | Language | 3 | Weighted mode | 38 | 0.003 (-0.062, 0.068) | 0.93 |
| DHA |  | Language | 5 | Inverse variance weighted | 38 | 0.021 (-0.055, 0.097) | 0.60 |
| DHA |  | Language | 5 | MR Egger | 38 | 0.060 (-0.052, 0.172) | 0.30 |
| DHA |  | Language | 5 | Weighted median | 38 | 0.043 (-0.057, 0.144) | 0.40 |
| DHA |  | Language | 5 | Weighted mode | 38 | 0.030 (-0.058, 0.118) | 0.51 |
| DHA |  | Motor | 3 | Inverse variance weighted | 38 | -0.054 (-0.121, 0.013) | 0.12 |
| DHA |  | Motor | 3 | MR Egger | 38 | -0.050 (-0.150, 0.051) | 0.34 |
| DHA |  | Motor | 3 | Weighted median | 38 | -0.050 (-0.127, 0.027) | 0.20 |
| DHA |  | Motor | 3 | Weighted mode | 38 | -0.043 (-0.120, 0.035) | 0.29 |
| DHA |  | Motor | 5 | Inverse variance weighted | 38 | 0.005 (-0.083, 0.094) | 0.90 |
| DHA |  | Motor | 5 | MR Egger | 38 | 0.076 (-0.053, 0.205) | 0.25 |
| DHA |  | Motor | 5 | Weighted median | 38 | 0.009 (-0.094, 0.112) | 0.86 |
| DHA |  | Motor | 5 | Weighted mode | 38 | 0.002 (-0.092, 0.096) | 0.96 |
| DHA | ADHD | Hyperactivity | 3 | Inverse variance weighted | 38 | -0.013 (-0.126, 0.100) | 0.82 |
| DHA | ADHD | Hyperactivity | 3 | MR Egger | 38 | 0.010 (-0.156, 0.176) | 0.90 |
| DHA | ADHD | Hyperactivity | 3 | Weighted median | 38 | 0.049 (-0.094, 0.193) | 0.50 |
| DHA | ADHD | Hyperactivity | 3 | Weighted mode | 38 | 0.024 (-0.119, 0.167) | 0.75 |
| DHA | ADHD | Hyperactivity | 5 | Inverse variance weighted | 38 | -0.093 (-0.186, -0.0003) | 0.05 |
| DHA | ADHD | Hyperactivity | 5 | MR Egger | 38 | -0.076 (-0.213, 0.060) | 0.28 |
| DHA | ADHD | Hyperactivity | 5 | Weighted median | 38 | -0.080 (-0.201, 0.041) | 0.19 |
| DHA | ADHD | Hyperactivity | 5 | Weighted mode | 38 | -0.088 (-0.201, 0.025) | 0.14 |
| DHA | ADHD | Hyperactivity | 8 | Inverse variance weighted | 38 | -0.310 (-0.553, -0.066) | 0.01 |
| DHA | ADHD | Hyperactivity | 8 | MR Egger | 38 | -0.043 (-0.387, 0.301) | 0.81 |
| DHA | ADHD | Hyperactivity | 8 | Weighted median | 38 | -0.054 (-0.324, 0.217) | 0.70 |
| DHA | ADHD | Hyperactivity | 8 | Weighted mode | 38 | -0.067 (-0.333, 0.198) | 0.62 |
| DHA | ADHD | Inattention | 3 | Inverse variance weighted | 38 | -0.027 (-0.121, 0.067) | 0.57 |
| DHA | ADHD | Inattention | 3 | MR Egger | 38 | -0.061 (-0.201, 0.079) | 0.40 |
| DHA | ADHD | Inattention | 3 | Weighted median | 38 | -0.044 (-0.160, 0.072) | 0.46 |
| DHA | ADHD | Inattention | 3 | Weighted mode | 38 | -0.054 (-0.158, 0.050) | 0.32 |
| DHA | ADHD | Inattention | 5 | Inverse variance weighted | 38 | -0.014 (-0.074, 0.045) | 0.64 |
| DHA | ADHD | Inattention | 5 | MR Egger | 38 | -0.013 (-0.102, 0.075) | 0.77 |
| DHA | ADHD | Inattention | 5 | Weighted median | 38 | -0.009 (-0.077, 0.059) | 0.79 |
| DHA | ADHD | Inattention | 5 | Weighted mode | 38 | -0.017 (-0.084, 0.050) | 0.62 |
| DHA | ADHD | Inattention | 8 | Inverse variance weighted | 38 | -0.168 (-0.444, 0.108) | 0.23 |
| DHA | ADHD | Inattention | 8 | MR Egger | 38 | -0.138 (-0.549, 0.274) | 0.52 |
| DHA | ADHD | Inattention | 8 | Weighted median | 38 | -0.066 (-0.353, 0.221) | 0.65 |
| DHA | ADHD | Inattention | 8 | Weighted mode | 38 | -0.091 (-0.381, 0.198) | 0.54 |
| DHA | ADHD | Total | 3 | Inverse variance weighted | 38 | -0.025 (-0.211, 0.161) | 0.79 |
| DHA | ADHD | Total | 3 | MR Egger | 38 | -0.025 (-0.304, 0.253) | 0.86 |
| DHA | ADHD | Total | 3 | Weighted median | 38 | 0.009 (-0.228, 0.246) | 0.94 |
| DHA | ADHD | Total | 3 | Weighted mode | 38 | -0.028 (-0.256, 0.199) | 0.81 |
| DHA | ADHD | Total | 5 | Inverse variance weighted | 38 | -0.106 (-0.239, 0.027) | 0.12 |
| DHA | ADHD | Total | 5 | MR Egger | 38 | -0.076 (-0.274, 0.122) | 0.46 |
| DHA | ADHD | Total | 5 | Weighted median | 38 | -0.074 (-0.239, 0.092) | 0.38 |
| DHA | ADHD | Total | 5 | Weighted mode | 38 | -0.085 (-0.251, 0.080) | 0.32 |
| DHA | ADHD | Total | 8 | Inverse variance weighted | 38 | -0.471 (-0.940, -0.002) | 0.05 |
| DHA | ADHD | Total | 8 | MR Egger | 38 | -0.176 (-0.864, 0.512) | 0.62 |
| DHA | ADHD | Total | 8 | Weighted median | 38 | -0.124 (-0.632, 0.385) | 0.63 |
| DHA | ADHD | Total | 8 | Weighted mode | 38 | -0.128 (-0.630, 0.375) | 0.62 |
| DHA | Autistic Traits | RRB | 3 | Inverse variance weighted | 38 | 0.025 (-0.100, 0.151) | 0.69 |
| DHA | Autistic Traits | RRB | 3 | MR Egger | 38 | 0.063 (-0.123, 0.250) | 0.51 |
| DHA | Autistic Traits | RRB | 3 | Weighted median | 38 | 0.058 (-0.098, 0.213) | 0.47 |
| DHA | Autistic Traits | RRB | 3 | Weighted mode | 38 | 0.054 (-0.103, 0.210) | 0.51 |
| DHA | Autistic Traits | RRB | 8 | Inverse variance weighted | 38 | -0.078 (-0.153, -0.003) | 0.04 |
| DHA | Autistic Traits | RRB | 8 | MR Egger | 38 | -0.023 (-0.132, 0.087) | 0.69 |
| DHA | Autistic Traits | RRB | 8 | Weighted median | 38 | -0.036 (-0.121, 0.048) | 0.40 |
| DHA | Autistic Traits | RRB | 8 | Weighted mode | 38 | -0.054 (-0.138, 0.031) | 0.22 |
| DHA | Autistic Traits | Social | 3 | Inverse variance weighted | 38 | 0.060 (-0.027, 0.148) | 0.18 |
| DHA | Autistic Traits | Social | 3 | MR Egger | 38 | 0.038 (-0.091, 0.167) | 0.56 |
| DHA | Autistic Traits | Social | 3 | Weighted median | 38 | 0.038 (-0.076, 0.151) | 0.51 |
| DHA | Autistic Traits | Social | 3 | Weighted mode | 38 | 0.047 (-0.060, 0.154) | 0.40 |
| DHA | Autistic Traits | Social | 8 | Inverse variance weighted | 38 | 0.091 (-0.047, 0.230) | 0.20 |
| DHA | Autistic Traits | Social | 8 | MR Egger | 38 | 0.092 (-0.112, 0.297) | 0.38 |
| DHA | Autistic Traits | Social | 8 | Weighted median | 38 | 0.097 (-0.072, 0.267) | 0.26 |
| DHA | Autistic Traits | Social | 8 | Weighted mode | 38 | 0.095 (-0.072, 0.263) | 0.27 |
| DHA | Autistic Traits | Total | 3 | Inverse variance weighted | 38 | 0.086 (-0.082, 0.254) | 0.32 |
| DHA | Autistic Traits | Total | 3 | MR Egger | 38 | 0.094 (-0.157, 0.345) | 0.47 |
| DHA | Autistic Traits | Total | 3 | Weighted median | 38 | 0.091 (-0.115, 0.297) | 0.39 |
| DHA | Autistic Traits | Total | 3 | Weighted mode | 38 | 0.089 (-0.119, 0.296) | 0.41 |
| DHA | Autistic Traits | Total | 8 | Inverse variance weighted | 38 | 0.013 (-0.152, 0.178) | 0.88 |
| DHA | Autistic Traits | Total | 8 | MR Egger | 38 | 0.063 (-0.180, 0.306) | 0.61 |
| DHA | Autistic Traits | Total | 8 | Weighted median | 38 | 0.037 (-0.169, 0.243) | 0.72 |
| DHA | Autistic Traits | Total | 8 | Weighted mode | 38 | 0.029 (-0.159, 0.218) | 0.76 |
| Revez Vitamin D |  | Language | 3 | Inverse variance weighted | 91 | 0.025 (-0.069, 0.120) | 0.60 |
| Revez Vitamin D |  | Language | 3 | MR Egger | 91 | 0.028 (-0.117, 0.172) | 0.71 |
| Revez Vitamin D |  | Language | 3 | Weighted median | 91 | 0.080 (-0.051, 0.211) | 0.23 |
| Revez Vitamin D |  | Language | 3 | Weighted mode | 91 | 0.037 (-0.079, 0.152) | 0.54 |
| Revez Vitamin D |  | Language | 5 | Inverse variance weighted | 91 | 0.035 (-0.099, 0.169) | 0.61 |
| Revez Vitamin D |  | Language | 5 | MR Egger | 91 | 0.094 (-0.109, 0.298) | 0.37 |
| Revez Vitamin D |  | Language | 5 | Weighted median | 91 | 0.028 (-0.142, 0.198) | 0.74 |
| Revez Vitamin D |  | Language | 5 | Weighted mode | 91 | 0.063 (-0.082, 0.207) | 0.40 |
| Revez Vitamin D |  | Motor | 3 | Inverse variance weighted | 91 | -0.077 (-0.174, 0.020) | 0.12 |
| Revez Vitamin D |  | Motor | 3 | MR Egger | 91 | -0.149 (-0.296, -0.002) | 0.05 |
| Revez Vitamin D |  | Motor | 3 | Weighted median | 91 | -0.203 (-0.346, -0.060) | 0.005 |
| Revez Vitamin D |  | Motor | 3 | Weighted mode | 91 | -0.150 (-0.284, -0.016) | 0.03 |
| Revez Vitamin D |  | Motor | 5 | Inverse variance weighted | 91 | 0.039 (-0.088, 0.165) | 0.55 |
| Revez Vitamin D |  | Motor | 5 | MR Egger | 91 | 0.076 (-0.117, 0.268) | 0.44 |
| Revez Vitamin D |  | Motor | 5 | Weighted median | 91 | 0.084 (-0.100, 0.268) | 0.37 |
| Revez Vitamin D |  | Motor | 5 | Weighted mode | 91 | 0.062 (-0.095, 0.219) | 0.44 |
| Revez Vitamin D | ADHD | Hyperactivity | 3 | Inverse variance weighted | 91 | -0.023 (-0.197, 0.151) | 0.80 |
| Revez Vitamin D | ADHD | Hyperactivity | 3 | MR Egger | 91 | -0.290 (-0.545, -0.035) | 0.03 |
| Revez Vitamin D | ADHD | Hyperactivity | 3 | Weighted median | 91 | -0.130 (-0.407, 0.147) | 0.36 |
| Revez Vitamin D | ADHD | Hyperactivity | 3 | Weighted mode | 91 | -0.121 (-0.347, 0.104) | 0.29 |
| Revez Vitamin D | ADHD | Hyperactivity | 5 | Inverse variance weighted | 91 | 0.024 (-0.122, 0.170) | 0.75 |
| Revez Vitamin D | ADHD | Hyperactivity | 5 | MR Egger | 91 | 0.052 (-0.170, 0.274) | 0.65 |
| Revez Vitamin D | ADHD | Hyperactivity | 5 | Weighted median | 91 | 0.024 (-0.196, 0.244) | 0.83 |
| Revez Vitamin D | ADHD | Hyperactivity | 5 | Weighted mode | 91 | 0.026 (-0.166, 0.219) | 0.79 |
| Revez Vitamin D | ADHD | Hyperactivity | 8 | Inverse variance weighted | 91 | 0.353 ( 0.028, 0.677) | 0.03 |
| Revez Vitamin D | ADHD | Hyperactivity | 8 | MR Egger | 91 | 0.173 (-0.319, 0.665) | 0.49 |
| Revez Vitamin D | ADHD | Hyperactivity | 8 | Weighted median | 91 | -0.123 (-0.644, 0.398) | 0.64 |
| Revez Vitamin D | ADHD | Hyperactivity | 8 | Weighted mode | 91 | -0.029 (-0.471, 0.414) | 0.90 |
| Revez Vitamin D | ADHD | Inattention | 3 | Inverse variance weighted | 91 | 0.009 (-0.132, 0.149) | 0.90 |
| Revez Vitamin D | ADHD | Inattention | 3 | MR Egger | 91 | -0.117 (-0.329, 0.094) | 0.28 |
| Revez Vitamin D | ADHD | Inattention | 3 | Weighted median | 91 | -0.115 (-0.330, 0.100) | 0.29 |
| Revez Vitamin D | ADHD | Inattention | 3 | Weighted mode | 91 | -0.085 (-0.269, 0.100) | 0.37 |
| Revez Vitamin D | ADHD | Inattention | 5 | Inverse variance weighted | 91 | 0.046 (-0.034, 0.127) | 0.26 |
| Revez Vitamin D | ADHD | Inattention | 5 | MR Egger | 91 | 0.083 (-0.039, 0.205) | 0.19 |
| Revez Vitamin D | ADHD | Inattention | 5 | Weighted median | 91 | 0.044 (-0.079, 0.168) | 0.48 |
| Revez Vitamin D | ADHD | Inattention | 5 | Weighted mode | 91 | 0.053 (-0.052, 0.158) | 0.33 |
| Revez Vitamin D | ADHD | Inattention | 8 | Inverse variance weighted | 91 | 0.327 (-0.080, 0.733) | 0.12 |
| Revez Vitamin D | ADHD | Inattention | 8 | MR Egger | 91 | 0.167 (-0.451, 0.786) | 0.60 |
| Revez Vitamin D | ADHD | Inattention | 8 | Weighted median | 91 | 0.190 (-0.356, 0.736) | 0.50 |
| Revez Vitamin D | ADHD | Inattention | 8 | Weighted mode | 91 | 0.033 (-0.468, 0.533) | 0.90 |
| Revez Vitamin D | ADHD | Total | 3 | Inverse variance weighted | 91 | -0.00010 (-0.278, 0.278) | 1.00 |
| Revez Vitamin D | ADHD | Total | 3 | MR Egger | 91 | -0.377 (-0.788, 0.035) | 0.08 |
| Revez Vitamin D | ADHD | Total | 3 | Weighted median | 91 | -0.279 (-0.707, 0.148) | 0.20 |
| Revez Vitamin D | ADHD | Total | 3 | Weighted mode | 91 | -0.149 (-0.482, 0.185) | 0.38 |
| Revez Vitamin D | ADHD | Total | 5 | Inverse variance weighted | 91 | 0.075 (-0.123, 0.272) | 0.46 |
| Revez Vitamin D | ADHD | Total | 5 | MR Egger | 91 | 0.129 (-0.171, 0.430) | 0.40 |
| Revez Vitamin D | ADHD | Total | 5 | Weighted median | 91 | 0.090 (-0.208, 0.388) | 0.55 |
| Revez Vitamin D | ADHD | Total | 5 | Weighted mode | 91 | 0.076 (-0.175, 0.326) | 0.55 |
| Revez Vitamin D | ADHD | Total | 8 | Inverse variance weighted | 91 | 0.674 ( 0.007, 1.341) | 0.05 |
| Revez Vitamin D | ADHD | Total | 8 | MR Egger | 91 | 0.340 (-0.672, 1.353) | 0.51 |
| Revez Vitamin D | ADHD | Total | 8 | Weighted median | 91 | 0.055 (-0.976, 1.087) | 0.92 |
| Revez Vitamin D | ADHD | Total | 8 | Weighted mode | 91 | 0.097 (-0.737, 0.932) | 0.82 |
| Revez Vitamin D | Autistic Traits | RRB | 3 | Inverse variance weighted | 91 | -0.049 (-0.235, 0.137) | 0.60 |
| Revez Vitamin D | Autistic Traits | RRB | 3 | MR Egger | 91 | -0.156 (-0.439, 0.126) | 0.28 |
| Revez Vitamin D | Autistic Traits | RRB | 3 | Weighted median | 91 | -0.112 (-0.403, 0.178) | 0.45 |
| Revez Vitamin D | Autistic Traits | RRB | 3 | Weighted mode | 91 | -0.065 (-0.315, 0.185) | 0.61 |
| Revez Vitamin D | Autistic Traits | RRB | 8 | Inverse variance weighted | 91 | 0.085 (-0.026, 0.195) | 0.13 |
| Revez Vitamin D | Autistic Traits | RRB | 8 | MR Egger | 91 | 0.00060 (-0.166, 0.167) | 0.99 |
| Revez Vitamin D | Autistic Traits | RRB | 8 | Weighted median | 91 | 0.043 (-0.117, 0.203) | 0.60 |
| Revez Vitamin D | Autistic Traits | RRB | 8 | Weighted mode | 91 | 0.002 (-0.127, 0.131) | 0.98 |
| Revez Vitamin D | Autistic Traits | Social | 3 | Inverse variance weighted | 91 | 0.057 (-0.075, 0.190) | 0.40 |
| Revez Vitamin D | Autistic Traits | Social | 3 | MR Egger | 91 | 0.086 (-0.116, 0.288) | 0.40 |
| Revez Vitamin D | Autistic Traits | Social | 3 | Weighted median | 91 | 0.109 (-0.099, 0.317) | 0.31 |
| Revez Vitamin D | Autistic Traits | Social | 3 | Weighted mode | 91 | 0.081 (-0.091, 0.253) | 0.36 |
| Revez Vitamin D | Autistic Traits | Social | 8 | Inverse variance weighted | 91 | 0.143 (-0.063, 0.349) | 0.17 |
| Revez Vitamin D | Autistic Traits | Social | 8 | MR Egger | 91 | -0.004 (-0.316, 0.309) | 0.98 |
| Revez Vitamin D | Autistic Traits | Social | 8 | Weighted median | 91 | -0.028 (-0.343, 0.287) | 0.86 |
| Revez Vitamin D | Autistic Traits | Social | 8 | Weighted mode | 91 | 0.024 (-0.244, 0.293) | 0.86 |
| Revez Vitamin D | Autistic Traits | Total | 3 | Inverse variance weighted | 91 | 0.005 (-0.237, 0.248) | 0.97 |
| Revez Vitamin D | Autistic Traits | Total | 3 | MR Egger | 91 | -0.073 (-0.442, 0.297) | 0.70 |
| Revez Vitamin D | Autistic Traits | Total | 3 | Weighted median | 91 | -0.021 (-0.419, 0.377) | 0.92 |
| Revez Vitamin D | Autistic Traits | Total | 3 | Weighted mode | 91 | -0.008 (-0.353, 0.336) | 0.96 |
| Revez Vitamin D | Autistic Traits | Total | 8 | Inverse variance weighted | 91 | 0.240 (-0.027, 0.507) | 0.08 |
| Revez Vitamin D | Autistic Traits | Total | 8 | MR Egger | 91 | 0.010 (-0.392, 0.412) | 0.96 |
| Revez Vitamin D | Autistic Traits | Total | 8 | Weighted median | 91 | -0.003 (-0.389, 0.383) | 0.99 |
| Revez Vitamin D | Autistic Traits | Total | 8 | Weighted mode | 91 | 0.066 (-0.257, 0.389) | 0.69 |
| Vitamin D |  | Language | 3 | Inverse variance weighted | 56 | -0.002 (-0.007, 0.004) | 0.60 |
| Vitamin D |  | Language | 3 | MR Egger | 56 | 0.003 (-0.013, 0.019) | 0.73 |
| Vitamin D |  | Language | 3 | Weighted median | 56 | -0.00007 (-0.011, 0.011) | 0.99 |
| Vitamin D |  | Language | 3 | Weighted mode | 56 | 0.001 (-0.010, 0.013) | 0.80 |
| Vitamin D |  | Language | 5 | Inverse variance weighted | 56 | 0.002 (-0.006, 0.010) | 0.59 |
| Vitamin D |  | Language | 5 | MR Egger | 56 | 0.013 (-0.009, 0.035) | 0.26 |
| Vitamin D |  | Language | 5 | Weighted median | 56 | 0.00100 (-0.013, 0.015) | 0.89 |
| Vitamin D |  | Language | 5 | Weighted mode | 56 | -0.006 (-0.024, 0.013) | 0.55 |
| Vitamin D |  | Motor | 3 | Inverse variance weighted | 56 | 0.001 (-0.005, 0.008) | 0.69 |
| Vitamin D |  | Motor | 3 | MR Egger | 56 | 0.005 (-0.014, 0.024) | 0.62 |
| Vitamin D |  | Motor | 3 | Weighted median | 56 | 0.002 (-0.012, 0.016) | 0.75 |
| Vitamin D |  | Motor | 3 | Weighted mode | 56 | 0.002 (-0.015, 0.019) | 0.82 |
| Vitamin D |  | Motor | 5 | Inverse variance weighted | 56 | 0.002 (-0.005, 0.010) | 0.55 |
| Vitamin D |  | Motor | 5 | MR Egger | 56 | 0.007 (-0.014, 0.028) | 0.51 |
| Vitamin D |  | Motor | 5 | Weighted median | 56 | 0.003 (-0.013, 0.018) | 0.74 |
| Vitamin D |  | Motor | 5 | Weighted mode | 56 | 0.005 (-0.017, 0.027) | 0.65 |
| Vitamin D | ADHD | Hyperactivity | 3 | Inverse variance weighted | 56 | -0.012 (-0.023, -0.002) | 0.02 |
| Vitamin D | ADHD | Hyperactivity | 3 | MR Egger | 56 | 0.012 (-0.017, 0.041) | 0.43 |
| Vitamin D | ADHD | Hyperactivity | 3 | Weighted median | 56 | -0.005 (-0.025, 0.016) | 0.65 |
| Vitamin D | ADHD | Hyperactivity | 3 | Weighted mode | 56 | 0.006 (-0.017, 0.029) | 0.60 |
| Vitamin D | ADHD | Hyperactivity | 5 | Inverse variance weighted | 56 | -0.014 (-0.022, -0.005) | 0.002 |
| Vitamin D | ADHD | Hyperactivity | 5 | MR Egger | 56 | -0.014 (-0.039, 0.011) | 0.29 |
| Vitamin D | ADHD | Hyperactivity | 5 | Weighted median | 56 | -0.012 (-0.032, 0.007) | 0.21 |
| Vitamin D | ADHD | Hyperactivity | 5 | Weighted mode | 56 | -0.012 (-0.036, 0.011) | 0.30 |
| Vitamin D | ADHD | Hyperactivity | 8 | Inverse variance weighted | 56 | -0.003 (-0.025, 0.018) | 0.76 |
| Vitamin D | ADHD | Hyperactivity | 8 | MR Egger | 56 | 0.003 (-0.059, 0.064) | 0.93 |
| Vitamin D | ADHD | Hyperactivity | 8 | Weighted median | 56 | 0.001 (-0.043, 0.045) | 0.96 |
| Vitamin D | ADHD | Hyperactivity | 8 | Weighted mode | 56 | -0.005 (-0.067, 0.058) | 0.89 |
| Vitamin D | ADHD | Inattention | 3 | Inverse variance weighted | 56 | -0.008 (-0.016, 0.0007) | 0.07 |
| Vitamin D | ADHD | Inattention | 3 | MR Egger | 56 | 0.004 (-0.020, 0.027) | 0.77 |
| Vitamin D | ADHD | Inattention | 3 | Weighted median | 56 | -0.008 (-0.025, 0.009) | 0.36 |
| Vitamin D | ADHD | Inattention | 3 | Weighted mode | 56 | -0.010 (-0.033, 0.013) | 0.41 |
| Vitamin D | ADHD | Inattention | 5 | Inverse variance weighted | 56 | -0.004 (-0.009, 0.001) | 0.15 |
| Vitamin D | ADHD | Inattention | 5 | MR Egger | 56 | -0.005 (-0.020, 0.010) | 0.51 |
| Vitamin D | ADHD | Inattention | 5 | Weighted median | 56 | -0.005 (-0.016, 0.006) | 0.39 |
| Vitamin D | ADHD | Inattention | 5 | Weighted mode | 56 | 0.004 (-0.010, 0.018) | 0.60 |
| Vitamin D | ADHD | Inattention | 8 | Inverse variance weighted | 56 | -0.008 (-0.035, 0.019) | 0.57 |
| Vitamin D | ADHD | Inattention | 8 | MR Egger | 56 | -0.043 (-0.120, 0.033) | 0.27 |
| Vitamin D | ADHD | Inattention | 8 | Weighted median | 56 | -0.003 (-0.048, 0.043) | 0.91 |
| Vitamin D | ADHD | Inattention | 8 | Weighted mode | 56 | -0.006 (-0.063, 0.051) | 0.83 |
| Vitamin D | ADHD | Total | 3 | Inverse variance weighted | 56 | -0.020 (-0.036, -0.003) | 0.02 |
| Vitamin D | ADHD | Total | 3 | MR Egger | 56 | 0.015 (-0.031, 0.062) | 0.52 |
| Vitamin D | ADHD | Total | 3 | Weighted median | 56 | -0.008 (-0.041, 0.025) | 0.64 |
| Vitamin D | ADHD | Total | 3 | Weighted mode | 56 | 0.004 (-0.036, 0.045) | 0.83 |
| Vitamin D | ADHD | Total | 5 | Inverse variance weighted | 56 | -0.017 (-0.029, -0.005) | 0.006 |
| Vitamin D | ADHD | Total | 5 | MR Egger | 56 | -0.018 (-0.053, 0.016) | 0.31 |
| Vitamin D | ADHD | Total | 5 | Weighted median | 56 | -0.016 (-0.042, 0.011) | 0.24 |
| Vitamin D | ADHD | Total | 5 | Weighted mode | 56 | -0.018 (-0.045, 0.009) | 0.20 |
| Vitamin D | ADHD | Total | 8 | Inverse variance weighted | 56 | -0.011 (-0.056, 0.034) | 0.62 |
| Vitamin D | ADHD | Total | 8 | MR Egger | 56 | -0.042 (-0.169, 0.086) | 0.53 |
| Vitamin D | ADHD | Total | 8 | Weighted median | 56 | -0.002 (-0.082, 0.077) | 0.95 |
| Vitamin D | ADHD | Total | 8 | Weighted mode | 56 | -0.010 (-0.124, 0.103) | 0.86 |
| Vitamin D | Autistic Traits | RRB | 3 | Inverse variance weighted | 56 | -0.017 (-0.028, -0.006) | 0.004 |
| Vitamin D | Autistic Traits | RRB | 3 | MR Egger | 56 | -0.002 (-0.034, 0.031) | 0.92 |
| Vitamin D | Autistic Traits | RRB | 3 | Weighted median | 56 | -0.012 (-0.037, 0.012) | 0.31 |
| Vitamin D | Autistic Traits | RRB | 3 | Weighted mode | 56 | -0.010 (-0.039, 0.019) | 0.49 |
| Vitamin D | Autistic Traits | RRB | 8 | Inverse variance weighted | 56 | -0.004 (-0.010, 0.002) | 0.20 |
| Vitamin D | Autistic Traits | RRB | 8 | MR Egger | 56 | 0.004 (-0.013, 0.021) | 0.65 |
| Vitamin D | Autistic Traits | RRB | 8 | Weighted median | 56 | -0.005 (-0.018, 0.008) | 0.42 |
| Vitamin D | Autistic Traits | RRB | 8 | Weighted mode | 56 | -0.006 (-0.021, 0.009) | 0.43 |
| Vitamin D | Autistic Traits | Social | 3 | Inverse variance weighted | 56 | 0.00090 (-0.007, 0.009) | 0.83 |
| Vitamin D | Autistic Traits | Social | 3 | MR Egger | 56 | 0.014 (-0.009, 0.038) | 0.23 |
| Vitamin D | Autistic Traits | Social | 3 | Weighted median | 56 | 0.005 (-0.011, 0.021) | 0.52 |
| Vitamin D | Autistic Traits | Social | 3 | Weighted mode | 56 | 0.013 (-0.008, 0.034) | 0.22 |
| Vitamin D | Autistic Traits | Social | 8 | Inverse variance weighted | 56 | 0.002 (-0.010, 0.015) | 0.72 |
| Vitamin D | Autistic Traits | Social | 8 | MR Egger | 56 | 0.00080 (-0.035, 0.037) | 0.97 |
| Vitamin D | Autistic Traits | Social | 8 | Weighted median | 56 | 0.002 (-0.025, 0.029) | 0.88 |
| Vitamin D | Autistic Traits | Social | 8 | Weighted mode | 56 | 0.005 (-0.029, 0.040) | 0.76 |
| Vitamin D | Autistic Traits | Total | 3 | Inverse variance weighted | 56 | -0.016 (-0.031, -0.001) | 0.03 |
| Vitamin D | Autistic Traits | Total | 3 | MR Egger | 56 | 0.015 (-0.027, 0.058) | 0.48 |
| Vitamin D | Autistic Traits | Total | 3 | Weighted median | 56 | -0.004 (-0.034, 0.025) | 0.77 |
| Vitamin D | Autistic Traits | Total | 3 | Weighted mode | 56 | 0.002 (-0.031, 0.035) | 0.91 |
| Vitamin D | Autistic Traits | Total | 8 | Inverse variance weighted | 56 | -0.003 (-0.018, 0.012) | 0.69 |
| Vitamin D | Autistic Traits | Total | 8 | MR Egger | 56 | 0.002 (-0.041, 0.045) | 0.93 |
| Vitamin D | Autistic Traits | Total | 8 | Weighted median | 56 | -0.007 (-0.038, 0.025) | 0.68 |
| Vitamin D | Autistic Traits | Total | 8 | Weighted mode | 56 | -0.005 (-0.049, 0.040) | 0.84 |

###

### Table S7. Bi-directional MR Results for Vitamin D/DHA and diagnoses of autism and ADHD

| **Exposure** | **Outcome** | **Method** | **N SNP** | **Beta/OR (95% CI)** | **P-value** |
| --- | --- | --- | --- | --- | --- |
| Vitamin D | ADHD | Inverse variance weighted | 68 | 1.132 (0.964, 1.329) | 0.13 |
| Vitamin D | ADHD | MR Egger | 68 | 1.107 (0.853, 1.435) | 0.45 |
| Vitamin D | ADHD | Weighted median | 68 | 1.045 (0.839, 1.302) | 0.69 |
| Vitamin D | ADHD | Weighted mode | 68 | 1.068 (0.878, 1.301) | 0.51 |
| Vitamin D | Autism | Inverse variance weighted | 56 | 1.060 (0.869, 1.293) | 0.56 |
| Vitamin D | Autism | MR Egger | 56 | 1.376 (1.014, 1.867) | 0.05 |
| Vitamin D | Autism | Weighted median | 56 | 1.127 (0.888, 1.429) | 0.33 |
| Vitamin D | Autism | Weighted mode | 56 | 1.171 (0.964, 1.424) | 0.12 |
| DHA | ADHD | Inverse variance weighted | 43 | 0.951 (0.868, 1.041) | 0.28 |
| DHA | ADHD | MR Egger | 43 | 1.069 (0.940, 1.216) | 0.32 |
| DHA | ADHD | Weighted median | 43 | 1.040 (0.944, 1.147) | 0.43 |
| DHA | ADHD | Weighted mode | 43 | 1.031 (0.939, 1.133) | 0.52 |
| DHA | Autism | Inverse variance weighted | 38 | 1.057 (0.946, 1.180) | 0.33 |
| DHA | Autism | MR Egger | 38 | 1.015 (0.862, 1.195) | 0.86 |
| DHA | Autism | Weighted median | 38 | 1.040 (0.936, 1.155) | 0.46 |
| DHA | Autism | Weighted mode | 38 | 1.062 (0.961, 1.172) | 0.24 |
| ADHD | Vitamin D | Inverse variance weighted | 63 | -0.001 (-0.008, 0.006) | 0.80 |
| ADHD | Vitamin D | MR Egger | 63 | -0.003 (-0.030, 0.023) | 0.81 |
| ADHD | Vitamin D | Weighted median | 63 | -0.004 (-0.012, 0.005) | 0.42 |
| ADHD | Vitamin D | Weighted mode | 63 | -0.014 (-0.038, 0.010) | 0.25 |
| Autism | Vitamin D | Inverse variance weighted | 34 | -0.011 (-0.020, -0.003) | 0.0090 |
| Autism | Vitamin D | Weighted median | 34 | -0.015 (-0.026, -0.003) | 0.01 |
| Autism | Vitamin D | Weighted mode | 34 | -0.024 (-0.047, -0.0003) | 0.06 |
| ADHD | DHA | Inverse variance weighted | 64 | -0.028 (-0.043, -0.013) | 0.0004 |
| ADHD | DHA | Weighted median | 64 | -0.012 (-0.027, 0.002) | 0.10 |
| ADHD | DHA | Weighted mode | 64 | 0.002 (-0.036, 0.039) | 0.94 |
| Autism | DHA | Inverse variance weighted | 34 | -0.016 (-0.037, 0.006) | 0.16 |
| Autism | DHA | Weighted median | 34 | -0.011 (-0.031, 0.009) | 0.27 |
| Autism | DHA | Weighted mode | 34 | -0.019 (-0.056, 0.018) | 0.32 |

Note. For the nutrient levels on diagnosis analysis, units can be interpreted as per SD unit increase in the nutrient level on odds of the outcome. For the analysis of diagnoses on nutrient levels, units can be interpreted as the average change in standardised units of nutrient levels, per doubling (2-fold increase) in the prevalence of the exposure.

### Table S8. Tests of instrument strength and regression dilution for the replication MR using PGC GWAS

| **Exposure** | **Outcome** | **Mean F Statistic** | **I2 Unweighted** | **I2 Weighted** |
| --- | --- | --- | --- | --- |
| Vitamin D | ADHD | 135.224 | 0.981 | 0.978 |
| Vitamin D | Autism | 135.224 | 0.981 | 0.978 |
| DHA | ADHD | 189.490 | 0.989 | 0.989 |
| DHA | Autism | 189.490 | 0.989 | 0.989 |
| ADHD | Vitamin D | 25.467 | 0.491 | 0.953 |
| Autism | Vitamin D | 24.179 | 0.651 | 0.000 |
| ADHD | DHA | 25.467 | 0.491 | 0.000 |
| Autism | DHA | 24.179 | 0.651 | 0.000 |

### Table S9. Cochran’s Q test of heterogeneity for replication MR using PGC GWAS

| **Exposure** | **Outcome** | **Q** | **df** | **P-value** |
| --- | --- | --- | --- | --- |
| Vitamin D | ADHD | 110.87 | 67 | <0.001 |
| Vitamin D | Autism | 110.80 | 55 | <0.001 |
| DHA | ADHD | 62.70 | 42 | 0.02 |
| DHA | Autism | 74.51 | 37 | <0.001 |
| ADHD | VitaminD | 106.28 | 62 | <0.001 |
| Autism | VitaminD | 40.95 | 33 | 0.16 |
| ADHD | DHA | 156.17 | 63 | <0.001 |
| Autism | DHA | 86.66 | 33 | <0.001 |

### Table S10. MR Egger Test for replication MR using PGC GWAS

| **Exposure** | **Outcome** | **Intercept (95% CI)** | **P-value** |
| --- | --- | --- | --- |
| Vitamin D | ADHD | 0.0009 (-0.007, 0.009) | 0.83 |
| Vitamin D | Autism | -0.011 (-0.020, -0.0010) | 0.04 |
| DHA | ADHD | -0.011 (-0.020, -0.002) | 0.02 |
| DHA | Autism | 0.004 (-0.008, 0.015) | 0.52 |
| ADHD | VitaminD | 0.0003 (-0.003, 0.004) | 0.86 |
| Autism | VitaminD | -0.0009 (-0.004, 0.002) | 0.59 |
| ADHD | DHA | -0.006 (-0.013, 0.0001) | 0.06 |
| Autism | DHA | 0.001 (-0.007, 0.009) | 0.78 |

###

### Table S11. Maternal PGS adjusted for child/father PGS

|  | | | | **DHA** | | | **Vitamin D (Manousaki et al.)** | | | **Vitamin D (Revez et al.)** | | |
| --- | --- | --- | --- | --- | --- | --- | --- | --- | --- | --- | --- | --- |
|  | **Domain** | **Age (Years)** | **Adjustment** | **N** | **Beta (95% CI)** | **P-value** | **N** | **Beta (95% CI)** | **P-value** | **N** | **Beta (95% CI)** | **P-value** |
|  | Language | 3 | Adjusted for child and father PRS | 16,298 | 0.012 (-0.008, 0.033) | 0.25 | 16,298 | 0.021 ( 0.00004, 0.042) | 0.05 | 16,298 | -0.00005 (-0.021, 0.021) | 1.00 |
|  | Language | 3 | Unadjusted | 23,713 | 0.005 (-0.010, 0.019) | 0.51 | 23,713 | 0.011 (-0.003, 0.026) | 0.12 | 23,713 | -0.003 (-0.018, 0.011) | 0.67 |
|  | Language | 5 | Adjusted for child and father PRS | 12,078 | 0.003 (-0.025, 0.031) | 0.84 | 12,078 | 0.015 (-0.012, 0.043) | 0.27 | 12,078 | 0.001 (-0.026, 0.029) | 0.93 |
|  | Language | 5 | Unadjusted | 16,796 | 0.015 (-0.004, 0.034) | 0.12 | 16,796 | 0.009 (-0.010, 0.028) | 0.35 | 16,796 | -0.005 (-0.024, 0.014) | 0.63 |
|  | Motor | 3 | Adjusted for child and father PRS | 16,255 | -0.003 (-0.028, 0.021) | 0.78 | 16,255 | 0.022 (-0.003, 0.047) | 0.08 | 16,255 | 0.005 (-0.020, 0.029) | 0.71 |
|  | Motor | 3 | Unadjusted | 23,641 | -0.007 (-0.024, 0.010) | 0.41 | 23,641 | 0.009 (-0.007, 0.026) | 0.26 | 23,641 | 0.001 (-0.016, 0.018) | 0.89 |
|  | Motor | 5 | Adjusted for child and father PRS | 12,113 | -0.011 (-0.042, 0.020) | 0.49 | 12,113 | -0.018 (-0.048, 0.013) | 0.26 | 12,113 | 0.004 (-0.027, 0.035) | 0.78 |
|  | Motor | 5 | Unadjusted | 16,838 | -0.007 (-0.028, 0.015) | 0.53 | 16,838 | 0.007 (-0.014, 0.028) | 0.51 | 16,838 | 0.016 (-0.005, 0.037) | 0.14 |
| ADHD | Hyperactivity | 3 | Adjusted for child and father PRS | 16,032 | 0.014 (-0.030, 0.057) | 0.54 | 16,032 | 0.026 (-0.018, 0.069) | 0.24 | 16,032 | -0.040 (-0.083, 0.003) | 0.07 |
| ADHD | Hyperactivity | 3 | Unadjusted | 23,310 | -0.002 (-0.031, 0.028) | 0.92 | 23,310 | -0.010 (-0.039, 0.020) | 0.51 | 23,310 | -0.022 (-0.051, 0.008) | 0.15 |
| ADHD | Hyperactivity | 5 | Adjusted for child and father PRS | 12,025 | 0.009 (-0.025, 0.044) | 0.59 | 12,025 | -0.016 (-0.051, 0.019) | 0.37 | 12,025 | -0.009 (-0.043, 0.026) | 0.63 |
| ADHD | Hyperactivity | 5 | Unadjusted | 16,731 | 0.006 (-0.018, 0.030) | 0.61 | 16,731 | -0.045 (-0.070, -0.021) | 0.0002 | 16,731 | -0.014 (-0.039, 0.010) | 0.24 |
| ADHD | Hyperactivity | 8 | Adjusted for child and father PRS | 12,451 | 0.034 (-0.049, 0.116) | 0.42 | 12,451 | 0.106 ( 0.024, 0.188) | 0.01 | 12,451 | -0.019 (-0.101, 0.063) | 0.66 |
| ADHD | Hyperactivity | 8 | Unadjusted | 17,724 | -0.002 (-0.058, 0.055) | 0.96 | 17,724 | 0.018 (-0.039, 0.075) | 0.54 | 17,724 | -0.017 (-0.074, 0.040) | 0.57 |
| ADHD | Inattention | 3 | Adjusted for child and father PRS | 16,042 | 0.014 (-0.021, 0.049) | 0.43 | 16,042 | 0.001 (-0.033, 0.036) | 0.94 | 16,042 | -0.050 (-0.084, -0.015) | 0.005 |
| ADHD | Inattention | 3 | Unadjusted | 23,318 | 0.004 (-0.020, 0.028) | 0.73 | 23,318 | -0.007 (-0.031, 0.016) | 0.55 | 23,318 | -0.028 (-0.052, -0.005) | 0.02 |
| ADHD | Inattention | 5 | Adjusted for child and father PRS | 12,053 | 0.015 (-0.004, 0.035) | 0.13 | 12,053 | 0.008 (-0.011, 0.028) | 0.40 | 12,053 | -0.020 (-0.039, -0.0004) | 0.05 |
| ADHD | Inattention | 5 | Unadjusted | 16,754 | 0.006 (-0.008, 0.020) | 0.38 | 16,754 | -0.010 (-0.023, 0.004) | 0.16 | 16,754 | -0.016 (-0.030, -0.003) | 0.02 |
| ADHD | Inattention | 8 | Adjusted for child and father PRS | 12,455 | 0.042 (-0.045, 0.128) | 0.35 | 12,455 | 0.033 (-0.053, 0.120) | 0.45 | 12,455 | -0.073 (-0.159, 0.014) | 0.10 |
| ADHD | Inattention | 8 | Unadjusted | 17,728 | 0.006 (-0.053, 0.066) | 0.84 | 17,728 | -0.013 (-0.072, 0.047) | 0.68 | 17,728 | -0.040 (-0.100, 0.020) | 0.20 |
| ADHD | Total | 3 | Adjusted for child and father PRS | 16,090 | 0.025 (-0.045, 0.094) | 0.49 | 16,090 | 0.029 (-0.040, 0.098) | 0.41 | 16,090 | -0.089 (-0.158, -0.020) | 0.01 |
| ADHD | Total | 3 | Unadjusted | 23,374 | 0.004 (-0.044, 0.051) | 0.88 | 23,374 | -0.014 (-0.062, 0.033) | 0.54 | 23,374 | -0.047 (-0.095, -0.0003) | 0.05 |
| ADHD | Total | 5 | Adjusted for child and father PRS | 12,112 | 0.023 (-0.024, 0.070) | 0.34 | 12,112 | -0.007 (-0.054, 0.041) | 0.78 | 12,112 | -0.028 (-0.075, 0.020) | 0.25 |
| ADHD | Total | 5 | Unadjusted | 16,841 | 0.011 (-0.022, 0.044) | 0.50 | 16,841 | -0.054 (-0.087, -0.021) | 0.0010 | 16,841 | -0.030 (-0.063, 0.003) | 0.07 |
| ADHD | Total | 8 | Adjusted for child and father PRS | 12,456 | 0.075 (-0.077, 0.226) | 0.33 | 12,456 | 0.141 (-0.010, 0.292) | 0.06 | 12,456 | -0.096 (-0.247, 0.055) | 0.21 |
| ADHD | Total | 8 | Unadjusted | 17,730 | 0.005 (-0.100, 0.109) | 0.93 | 17,730 | 0.006 (-0.098, 0.111) | 0.90 | 17,730 | -0.059 (-0.164, 0.045) | 0.27 |
| Autistic Traits | RRB | 3 | Adjusted for child and father PRS | 16,266 | 0.005 (-0.042, 0.052) | 0.85 | 16,266 | 0.040 (-0.007, 0.088) | 0.09 | 16,266 | 0.008 (-0.039, 0.055) | 0.74 |
| Autistic Traits | RRB | 3 | Unadjusted | 23,652 | -0.005 (-0.038, 0.027) | 0.74 | 23,652 | -0.008 (-0.040, 0.024) | 0.63 | 23,652 | -0.019 (-0.051, 0.013) | 0.25 |
| Autistic Traits | RRB | 8 | Adjusted for child and father PRS | 12,041 | 0.008 (-0.017, 0.034) | 0.51 | 12,041 | 0.018 (-0.007, 0.043) | 0.16 | 12,041 | -0.014 (-0.039, 0.011) | 0.28 |
| Autistic Traits | RRB | 8 | Unadjusted | 17,128 | -0.005 (-0.022, 0.012) | 0.54 | 17,128 | 0.008 (-0.009, 0.025) | 0.37 | 17,128 | -0.007 (-0.024, 0.010) | 0.41 |
| Autistic Traits | Social | 3 | Adjusted for child and father PRS | 16,297 | 0.022 (-0.011, 0.055) | 0.19 | 16,297 | 0.041 ( 0.008, 0.074) | 0.01 | 16,297 | 0.022 (-0.011, 0.055) | 0.20 |
| Autistic Traits | Social | 3 | Unadjusted | 23,698 | 0.010 (-0.013, 0.033) | 0.39 | 23,698 | 0.014 (-0.009, 0.036) | 0.23 | 23,698 | 0.005 (-0.018, 0.028) | 0.68 |
| Autistic Traits | Social | 8 | Adjusted for child and father PRS | 12,382 | -0.032 (-0.085, 0.020) | 0.23 | 12,382 | -0.009 (-0.062, 0.043) | 0.73 | 12,382 | 0.027 (-0.026, 0.079) | 0.32 |
| Autistic Traits | Social | 8 | Unadjusted | 17,636 | 0.012 (-0.024, 0.049) | 0.50 | 17,636 | 0.002 (-0.034, 0.038) | 0.92 | 17,636 | 0.017 (-0.019, 0.053) | 0.36 |
| Autistic Traits | Total | 3 | Adjusted for child and father PRS | 16,303 | 0.028 (-0.034, 0.090) | 0.38 | 16,303 | 0.079 ( 0.017, 0.141) | 0.01 | 16,303 | 0.023 (-0.039, 0.085) | 0.46 |
| Autistic Traits | Total | 3 | Unadjusted | 23,705 | 0.005 (-0.037, 0.048) | 0.81 | 23,705 | 0.003 (-0.040, 0.045) | 0.90 | 23,705 | -0.023 (-0.065, 0.020) | 0.30 |
| Autistic Traits | Total | 8 | Adjusted for child and father PRS | 12,391 | -0.028 (-0.090, 0.035) | 0.39 | 12,391 | 0.016 (-0.046, 0.078) | 0.61 | 12,391 | 0.025 (-0.037, 0.088) | 0.42 |
| Autistic Traits | Total | 8 | Unadjusted | 17,647 | 0.004 (-0.039, 0.047) | 0.87 | 17,647 | 0.010 (-0.033, 0.053) | 0.64 | 17,647 | 0.015 (-0.028, 0.058) | 0.50 |

### Table S12. Maternal DHA PGS adjusted for child/father PGS and stratified by child sex

|  | | | | **Female** | | | **Male** | | |
| --- | --- | --- | --- | --- | --- | --- | --- | --- | --- |
|  | **Domain** | **Age (Years)** | **Adjustment** | **N** | **Beta (95% CI)** | **P-value** | **N** | **Beta (95% CI)** | **P-value** |
|  | Language | 3 | Adjusted for child and father PRS | 7,976 | 0.007 (-0.017, 0.032) | 0.55 | 8,322 | 0.016 (-0.017, 0.049) | 0.34 |
|  | Language | 3 | Unadjusted | 11,582 | 0.007 (-0.010, 0.024) | 0.43 | 12,131 | 0.002 (-0.021, 0.025) | 0.86 |
|  | Language | 5 | Adjusted for child and father PRS | 5,917 | -0.002 (-0.038, 0.034) | 0.91 | 6,161 | 0.007 (-0.035, 0.049) | 0.74 |
|  | Language | 5 | Unadjusted | 8,240 | 0.007 (-0.018, 0.032) | 0.58 | 8,556 | 0.022 (-0.007, 0.051) | 0.13 |
|  | Motor | 3 | Adjusted for child and father PRS | 7,956 | -0.016 (-0.047, 0.014) | 0.30 | 8,299 | 0.008 (-0.030, 0.046) | 0.69 |
|  | Motor | 3 | Unadjusted | 11,551 | -0.014 (-0.035, 0.007) | 0.18 | 12,090 | -0.0009 (-0.027, 0.025) | 0.95 |
|  | Motor | 5 | Adjusted for child and father PRS | 5,933 | -0.028 (-0.063, 0.006) | 0.11 | 6,180 | 0.007 (-0.043, 0.058) | 0.78 |
|  | Motor | 5 | Unadjusted | 8,259 | -0.017 (-0.040, 0.007) | 0.16 | 8,579 | 0.005 (-0.031, 0.040) | 0.80 |
| ADHD | Hyperactivity | 3 | Adjusted for child and father PRS | 7,837 | 0.001 (-0.061, 0.063) | 0.97 | 8,195 | 0.023 (-0.037, 0.083) | 0.46 |
| ADHD | Hyperactivity | 3 | Unadjusted | 11,378 | -0.002 (-0.044, 0.040) | 0.92 | 11,932 | -0.001 (-0.043, 0.040) | 0.95 |
| ADHD | Hyperactivity | 5 | Adjusted for child and father PRS | 5,887 | -0.004 (-0.052, 0.044) | 0.88 | 6,138 | 0.021 (-0.030, 0.071) | 0.42 |
| ADHD | Hyperactivity | 5 | Unadjusted | 8,201 | -0.002 (-0.036, 0.031) | 0.89 | 8,530 | 0.014 (-0.021, 0.049) | 0.43 |
| ADHD | Hyperactivity | 8 | Adjusted for child and father PRS | 6,056 | 0.022 (-0.084, 0.128) | 0.69 | 6,395 | 0.040 (-0.085, 0.164) | 0.53 |
| ADHD | Hyperactivity | 8 | Unadjusted | 8,671 | -0.006 (-0.079, 0.066) | 0.87 | 9,053 | -0.002 (-0.089, 0.085) | 0.97 |
| ADHD | Inattention | 3 | Adjusted for child and father PRS | 7,843 | -0.008 (-0.057, 0.041) | 0.75 | 8,199 | 0.032 (-0.017, 0.081) | 0.20 |
| ADHD | Inattention | 3 | Unadjusted | 11,386 | -0.008 (-0.041, 0.025) | 0.64 | 11,932 | 0.015 (-0.019, 0.048) | 0.39 |
| ADHD | Inattention | 5 | Adjusted for child and father PRS | 5,903 | 0.018 (-0.009, 0.045) | 0.20 | 6,150 | 0.012 (-0.016, 0.040) | 0.41 |
| ADHD | Inattention | 5 | Unadjusted | 8,218 | -0.0009 (-0.019, 0.018) | 0.92 | 8,536 | 0.012 (-0.007, 0.032) | 0.22 |
| ADHD | Inattention | 8 | Adjusted for child and father PRS | 6,056 | 0.055 (-0.056, 0.166) | 0.33 | 6,399 | 0.026 (-0.105, 0.157) | 0.70 |
| ADHD | Inattention | 8 | Unadjusted | 8,671 | -0.008 (-0.085, 0.069) | 0.83 | 9,057 | 0.017 (-0.074, 0.107) | 0.72 |
| ADHD | Total | 3 | Adjusted for child and father PRS | 7,865 | -0.005 (-0.103, 0.094) | 0.93 | 8,225 | 0.047 (-0.049, 0.144) | 0.34 |
| ADHD | Total | 3 | Unadjusted | 11,411 | -0.007 (-0.074, 0.060) | 0.83 | 11,963 | 0.012 (-0.054, 0.078) | 0.72 |
| ADHD | Total | 5 | Adjusted for child and father PRS | 5,929 | 0.011 (-0.054, 0.076) | 0.74 | 6,183 | 0.032 (-0.037, 0.101) | 0.36 |
| ADHD | Total | 5 | Unadjusted | 8,255 | -0.004 (-0.049, 0.041) | 0.85 | 8,586 | 0.025 (-0.023, 0.074) | 0.30 |
| ADHD | Total | 8 | Adjusted for child and father PRS | 6,058 | 0.075 (-0.119, 0.268) | 0.45 | 6,398 | 0.066 (-0.164, 0.296) | 0.57 |
| ADHD | Total | 8 | Unadjusted | 8,673 | -0.015 (-0.148, 0.118) | 0.83 | 9,057 | 0.015 (-0.145, 0.175) | 0.86 |
| Autistic Traits | RRB | 3 | Adjusted for child and father PRS | 7,955 | -0.015 (-0.080, 0.050) | 0.65 | 8,311 | 0.021 (-0.046, 0.089) | 0.54 |
| Autistic Traits | RRB | 3 | Unadjusted | 11,552 | -0.020 (-0.064, 0.025) | 0.38 | 12,100 | 0.008 (-0.038, 0.055) | 0.72 |
| Autistic Traits | RRB | 8 | Adjusted for child and father PRS | 5,858 | -0.003 (-0.035, 0.029) | 0.86 | 6,183 | 0.019 (-0.020, 0.057) | 0.34 |
| Autistic Traits | RRB | 8 | Unadjusted | 8,375 | -0.003 (-0.025, 0.018) | 0.76 | 8,753 | -0.007 (-0.034, 0.019) | 0.58 |
| Autistic Traits | Social | 3 | Adjusted for child and father PRS | 7,975 | -0.012 (-0.054, 0.031) | 0.59 | 8,322 | 0.054 ( 0.004, 0.105) | 0.03 |
| Autistic Traits | Social | 3 | Unadjusted | 11,577 | -0.014 (-0.043, 0.016) | 0.36 | 12,121 | 0.032 (-0.002, 0.067) | 0.07 |
| Autistic Traits | Social | 8 | Adjusted for child and father PRS | 6,018 | -0.036 (-0.107, 0.034) | 0.31 | 6,364 | -0.030 (-0.106, 0.047) | 0.45 |
| Autistic Traits | Social | 8 | Unadjusted | 8,622 | 0.002 (-0.047, 0.051) | 0.94 | 9,014 | 0.020 (-0.033, 0.074) | 0.46 |
| Autistic Traits | Total | 3 | Adjusted for child and father PRS | 7,975 | -0.024 (-0.108, 0.061) | 0.58 | 8,328 | 0.075 (-0.016, 0.165) | 0.11 |
| Autistic Traits | Total | 3 | Unadjusted | 11,580 | -0.030 (-0.088, 0.027) | 0.30 | 12,125 | 0.039 (-0.024, 0.101) | 0.23 |
| Autistic Traits | Total | 8 | Adjusted for child and father PRS | 6,023 | -0.046 (-0.128, 0.035) | 0.27 | 6,368 | -0.012 (-0.106, 0.081) | 0.80 |
| Autistic Traits | Total | 8 | Unadjusted | 8,629 | -0.007 (-0.063, 0.049) | 0.80 | 9,018 | 0.011 (-0.053, 0.076) | 0.73 |

### Table S13. Maternal Vitamin D PGS adjusted for child/father PGS and stratified by child sex

|  | | | | **Female** | | | **Male** | | |
| --- | --- | --- | --- | --- | --- | --- | --- | --- | --- |
|  | **Domain** | **Age (Years)** | **Adjustment** | **N** | **Beta (95% CI)** | **P-value** | **N** | **Beta (95% CI)** | **P-value** |
|  | Language | 3 | Adjusted for child and father PRS | 7,976 | 0.003 (-0.021, 0.028) | 0.78 | 8,322 | 0.040 ( 0.006, 0.073) | 0.02 |
|  | Language | 3 | Unadjusted | 11,582 | 0.007 (-0.010, 0.024) | 0.42 | 12,131 | 0.016 (-0.006, 0.038) | 0.16 |
|  | Language | 5 | Adjusted for child and father PRS | 5,917 | 0.007 (-0.028, 0.043) | 0.70 | 6,161 | 0.024 (-0.018, 0.066) | 0.27 |
|  | Language | 5 | Unadjusted | 8,240 | 0.016 (-0.010, 0.041) | 0.23 | 8,556 | 0.003 (-0.026, 0.031) | 0.84 |
|  | Motor | 3 | Adjusted for child and father PRS | 7,956 | 0.017 (-0.014, 0.047) | 0.28 | 8,299 | 0.027 (-0.011, 0.065) | 0.17 |
|  | Motor | 3 | Unadjusted | 11,551 | 0.013 (-0.007, 0.034) | 0.20 | 12,090 | 0.006 (-0.020, 0.031) | 0.65 |
|  | Motor | 5 | Adjusted for child and father PRS | 5,933 | -0.032 (-0.066, 0.002) | 0.06 | 6,180 | -0.006 (-0.056, 0.045) | 0.83 |
|  | Motor | 5 | Unadjusted | 8,259 | -0.012 (-0.036, 0.011) | 0.31 | 8,579 | 0.024 (-0.010, 0.059) | 0.17 |
| ADHD | Hyperactivity | 3 | Adjusted for child and father PRS | 7,837 | 0.031 (-0.030, 0.092) | 0.33 | 8,195 | 0.023 (-0.037, 0.084) | 0.45 |
| ADHD | Hyperactivity | 3 | Unadjusted | 11,378 | 0.009 (-0.033, 0.051) | 0.66 | 11,932 | -0.027 (-0.068, 0.013) | 0.19 |
| ADHD | Hyperactivity | 5 | Adjusted for child and father PRS | 5,887 | -0.007 (-0.054, 0.041) | 0.78 | 6,138 | -0.024 (-0.074, 0.026) | 0.35 |
| ADHD | Hyperactivity | 5 | Unadjusted | 8,201 | -0.034 (-0.067, -0.0008) | 0.04 | 8,530 | -0.055 (-0.090, -0.021) | 0.002 |
| ADHD | Hyperactivity | 8 | Adjusted for child and father PRS | 6,056 | 0.131 ( 0.028, 0.234) | 0.01 | 6,395 | 0.086 (-0.040, 0.211) | 0.18 |
| ADHD | Hyperactivity | 8 | Unadjusted | 8,671 | 0.036 (-0.037, 0.109) | 0.34 | 9,053 | 0.003 (-0.084, 0.089) | 0.95 |
| ADHD | Inattention | 3 | Adjusted for child and father PRS | 7,843 | -0.008 (-0.056, 0.040) | 0.74 | 8,199 | 0.012 (-0.037, 0.061) | 0.62 |
| ADHD | Inattention | 3 | Unadjusted | 11,386 | -0.015 (-0.048, 0.018) | 0.37 | 11,932 | 0.001 (-0.032, 0.034) | 0.94 |
| ADHD | Inattention | 5 | Adjusted for child and father PRS | 5,903 | 0.0009 (-0.025, 0.027) | 0.95 | 6,150 | 0.015 (-0.013, 0.044) | 0.29 |
| ADHD | Inattention | 5 | Unadjusted | 8,218 | -0.014 (-0.032, 0.005) | 0.14 | 8,536 | -0.005 (-0.024, 0.015) | 0.64 |
| ADHD | Inattention | 8 | Adjusted for child and father PRS | 6,056 | 0.093 (-0.015, 0.201) | 0.09 | 6,399 | -0.024 (-0.156, 0.108) | 0.72 |
| ADHD | Inattention | 8 | Unadjusted | 8,671 | 0.024 (-0.053, 0.102) | 0.54 | 9,057 | -0.043 (-0.134, 0.047) | 0.34 |
| ADHD | Total | 3 | Adjusted for child and father PRS | 7,865 | 0.026 (-0.071, 0.123) | 0.60 | 8,225 | 0.036 (-0.061, 0.133) | 0.47 |
| ADHD | Total | 3 | Unadjusted | 11,411 | -0.003 (-0.070, 0.064) | 0.93 | 11,963 | -0.023 (-0.089, 0.042) | 0.48 |
| ADHD | Total | 5 | Adjusted for child and father PRS | 5,929 | -0.009 (-0.073, 0.055) | 0.78 | 6,183 | -0.004 (-0.073, 0.065) | 0.91 |
| ADHD | Total | 5 | Unadjusted | 8,255 | -0.049 (-0.094, -0.005) | 0.03 | 8,586 | -0.057 (-0.104, -0.010) | 0.02 |
| ADHD | Total | 8 | Adjusted for child and father PRS | 6,058 | 0.224 ( 0.036, 0.412) | 0.02 | 6,398 | 0.066 (-0.167, 0.298) | 0.58 |
| ADHD | Total | 8 | Unadjusted | 8,673 | 0.061 (-0.072, 0.194) | 0.37 | 9,057 | -0.040 (-0.199, 0.120) | 0.63 |
| Autistic Traits | RRB | 3 | Adjusted for child and father PRS | 7,955 | 0.021 (-0.044, 0.085) | 0.53 | 8,311 | 0.064 (-0.004, 0.132) | 0.07 |
| Autistic Traits | RRB | 3 | Unadjusted | 11,552 | 0.005 (-0.039, 0.049) | 0.82 | 12,100 | -0.018 (-0.064, 0.028) | 0.44 |
| Autistic Traits | RRB | 8 | Adjusted for child and father PRS | 5,858 | 0.0006 (-0.031, 0.032) | 0.97 | 6,183 | 0.035 (-0.004, 0.074) | 0.08 |
| Autistic Traits | RRB | 8 | Unadjusted | 8,375 | 0.010 (-0.012, 0.032) | 0.38 | 8,753 | 0.006 (-0.021, 0.032) | 0.67 |
| Autistic Traits | Social | 3 | Adjusted for child and father PRS | 7,975 | 0.049 ( 0.007, 0.090) | 0.02 | 8,322 | 0.035 (-0.016, 0.085) | 0.17 |
| Autistic Traits | Social | 3 | Unadjusted | 11,577 | 0.024 (-0.006, 0.053) | 0.11 | 12,121 | 0.005 (-0.029, 0.039) | 0.76 |
| Autistic Traits | Social | 8 | Adjusted for child and father PRS | 6,018 | -0.010 (-0.079, 0.059) | 0.77 | 6,364 | -0.006 (-0.083, 0.072) | 0.88 |
| Autistic Traits | Social | 8 | Unadjusted | 8,622 | -0.004 (-0.053, 0.045) | 0.88 | 9,014 | 0.008 (-0.045, 0.061) | 0.77 |
| Autistic Traits | Total | 3 | Adjusted for child and father PRS | 7,975 | 0.070 (-0.013, 0.152) | 0.10 | 8,328 | 0.093 ( 0.001, 0.184) | 0.05 |
| Autistic Traits | Total | 3 | Unadjusted | 11,580 | 0.028 (-0.029, 0.085) | 0.34 | 12,125 | -0.018 (-0.080, 0.044) | 0.57 |
| Autistic Traits | Total | 8 | Adjusted for child and father PRS | 6,023 | -0.004 (-0.083, 0.075) | 0.92 | 6,368 | 0.038 (-0.056, 0.132) | 0.43 |
| Autistic Traits | Total | 8 | Unadjusted | 8,629 | 0.007 (-0.050, 0.063) | 0.81 | 9,018 | 0.015 (-0.050, 0.080) | 0.65 |

### Table S14. Child PGS adjusted for maternal and paternal PGS

|  | | | | **DHA** | | | **Vitamin D (Manousaki et al.)** | | | **Vitamin D (Revez et al.)** | | |
| --- | --- | --- | --- | --- | --- | --- | --- | --- | --- | --- | --- | --- |
|  | **Domain** | **Age (Years)** | **Adjustment** | **N** | **Beta (95% CI)** | **P-value** | **N** | **Beta (95% CI)** | **P-value** | **N** | **Beta (95% CI)** | **P-value** |
|  | Language | 3 | Adjusted for mother and father PRS | 16,298 | -0.010 (-0.033, 0.014) | 0.44 | 16,298 | -0.012 (-0.036, 0.012) | 0.34 | 16,298 | -0.002 (-0.025, 0.022) | 0.90 |
|  | Language | 3 | Unadjusted | 26,646 | -0.002 (-0.016, 0.011) | 0.74 | 26,646 | -0.002 (-0.016, 0.011) | 0.76 | 26,646 | 0.001 (-0.012, 0.015) | 0.88 |
|  | Language | 5 | Adjusted for mother and father PRS | 12,078 | 0.007 (-0.025, 0.039) | 0.67 | 12,078 | 0.015 (-0.018, 0.047) | 0.37 | 12,078 | 0.007 (-0.025, 0.039) | 0.66 |
|  | Language | 5 | Unadjusted | 18,872 | 0.007 (-0.011, 0.025) | 0.46 | 18,872 | 0.010 (-0.008, 0.028) | 0.29 | 18,872 | 0.001 (-0.017, 0.019) | 0.91 |
|  | Motor | 3 | Adjusted for mother and father PRS | 16,255 | -0.008 (-0.036, 0.020) | 0.58 | 16,255 | -0.009 (-0.038, 0.019) | 0.53 | 16,255 | -0.004 (-0.033, 0.024) | 0.76 |
|  | Motor | 3 | Unadjusted | 26,564 | -0.009 (-0.025, 0.006) | 0.25 | 26,564 | 0.001 (-0.014, 0.017) | 0.87 | 26,564 | -0.015 (-0.031, 0.0001) | 0.05 |
|  | Motor | 5 | Adjusted for mother and father PRS | 12,113 | 0.005 (-0.031, 0.041) | 0.79 | 12,113 | 0.019 (-0.017, 0.054) | 0.30 | 12,113 | 0.007 (-0.029, 0.043) | 0.70 |
|  | Motor | 5 | Unadjusted | 18,914 | -0.003 (-0.023, 0.017) | 0.76 | 18,914 | 0.009 (-0.011, 0.029) | 0.37 | 18,914 | 0.012 (-0.008, 0.032) | 0.25 |
| ADHD | Hyperactivity | 3 | Adjusted for mother and father PRS | 16,032 | -0.008 (-0.058, 0.041) | 0.75 | 16,032 | -0.035 (-0.084, 0.015) | 0.17 | 16,032 | 0.033 (-0.017, 0.082) | 0.20 |
| ADHD | Hyperactivity | 3 | Unadjusted | 26,207 | -0.004 (-0.031, 0.024) | 0.79 | 26,207 | -0.030 (-0.058, -0.003) | 0.03 | 26,207 | -0.001 (-0.029, 0.027) | 0.94 |
| ADHD | Hyperactivity | 5 | Adjusted for mother and father PRS | 12,025 | -0.008 (-0.048, 0.032) | 0.69 | 12,025 | -0.035 (-0.075, 0.005) | 0.08 | 12,025 | -0.004 (-0.044, 0.036) | 0.85 |
| ADHD | Hyperactivity | 5 | Unadjusted | 18,793 | -0.024 (-0.047, -0.002) | 0.04 | 18,793 | -0.046 (-0.068, -0.023) | 0.00008 | 18,793 | 0.003 (-0.019, 0.026) | 0.78 |
| ADHD | Hyperactivity | 8 | Adjusted for mother and father PRS | 12,451 | -0.066 (-0.161, 0.029) | 0.18 | 12,451 | -0.071 (-0.166, 0.024) | 0.14 | 12,451 | 0.073 (-0.022, 0.168) | 0.13 |
| ADHD | Hyperactivity | 8 | Unadjusted | 19,971 | -0.063 (-0.116, -0.010) | 0.02 | 19,971 | -0.028 (-0.082, 0.025) | 0.30 | 19,971 | 0.032 (-0.021, 0.085) | 0.24 |
| ADHD | Inattention | 3 | Adjusted for mother and father PRS | 16,042 | 0.001 (-0.038, 0.041) | 0.94 | 16,042 | -0.036 (-0.076, 0.004) | 0.08 | 16,042 | 0.027 (-0.013, 0.067) | 0.18 |
| ADHD | Inattention | 3 | Unadjusted | 26,192 | -0.002 (-0.024, 0.021) | 0.89 | 26,192 | -0.022 (-0.044, 0.0005) | 0.06 | 26,192 | 0.002 (-0.020, 0.025) | 0.83 |
| ADHD | Inattention | 5 | Adjusted for mother and father PRS | 12,053 | -0.019 (-0.041, 0.004) | 0.11 | 12,053 | -0.024 (-0.046, -0.001) | 0.04 | 12,053 | 0.013 (-0.010, 0.035) | 0.27 |
| ADHD | Inattention | 5 | Unadjusted | 18,819 | -0.003 (-0.016, 0.010) | 0.63 | 18,819 | -0.009 (-0.022, 0.004) | 0.17 | 18,819 | 0.009 (-0.004, 0.021) | 0.18 |
| ADHD | Inattention | 8 | Adjusted for mother and father PRS | 12,455 | -0.072 (-0.172, 0.028) | 0.16 | 12,455 | -0.032 (-0.132, 0.068) | 0.53 | 12,455 | 0.105 ( 0.005, 0.205) | 0.04 |
| ADHD | Inattention | 8 | Unadjusted | 19,975 | -0.038 (-0.095, 0.018) | 0.18 | 19,975 | -0.033 (-0.089, 0.024) | 0.26 | 19,975 | 0.026 (-0.030, 0.082) | 0.36 |
| ADHD | Total | 3 | Adjusted for mother and father PRS | 16,090 | -0.002 (-0.082, 0.077) | 0.95 | 16,090 | -0.068 (-0.148, 0.011) | 0.09 | 16,090 | 0.062 (-0.017, 0.141) | 0.13 |
| ADHD | Total | 3 | Unadjusted | 26,266 | -0.0008 (-0.045, 0.043) | 0.97 | 26,266 | -0.049 (-0.093, -0.005) | 0.03 | 26,266 | 0.003 (-0.041, 0.047) | 0.89 |
| ADHD | Total | 5 | Adjusted for mother and father PRS | 12,112 | -0.025 (-0.080, 0.030) | 0.37 | 12,112 | -0.058 (-0.112, -0.003) | 0.04 | 12,112 | 0.010 (-0.045, 0.065) | 0.73 |
| ADHD | Total | 5 | Unadjusted | 18,918 | -0.027 (-0.058, 0.004) | 0.08 | 18,918 | -0.054 (-0.085, -0.023) | 0.00060 | 18,918 | 0.012 (-0.019, 0.043) | 0.44 |
| ADHD | Total | 8 | Adjusted for mother and father PRS | 12,456 | -0.136 (-0.311, 0.038) | 0.13 | 12,456 | -0.105 (-0.280, 0.070) | 0.24 | 12,456 | 0.178 ( 0.003, 0.352) | 0.05 |
| ADHD | Total | 8 | Unadjusted | 19,977 | -0.100 (-0.198, -0.002) | 0.05 | 19,977 | -0.062 (-0.160, 0.036) | 0.22 | 19,977 | 0.057 (-0.041, 0.155) | 0.25 |
| Autistic Traits | RRB | 3 | Adjusted for mother and father PRS | 16,266 | -0.015 (-0.069, 0.039) | 0.59 | 16,266 | -0.027 (-0.081, 0.027) | 0.32 | 16,266 | -0.005 (-0.059, 0.049) | 0.85 |
| Autistic Traits | RRB | 3 | Unadjusted | 26,582 | 0.009 (-0.021, 0.039) | 0.55 | 26,582 | -0.043 (-0.073, -0.013) | 0.00500 | 26,582 | -0.013 (-0.043, 0.017) | 0.41 |
| Autistic Traits | RRB | 8 | Adjusted for mother and father PRS | 12,041 | -0.023 (-0.052, 0.006) | 0.12 | 12,041 | -0.005 (-0.034, 0.024) | 0.73 | 12,041 | 0.022 (-0.007, 0.051) | 0.13 |
| Autistic Traits | RRB | 8 | Unadjusted | 19,299 | -0.019 (-0.035, -0.003) | 0.02 | 19,299 | -0.009 (-0.026, 0.007) | 0.26 | 19,299 | 0.008 (-0.008, 0.024) | 0.35 |
| Autistic Traits | Social | 3 | Adjusted for mother and father PRS | 16,297 | 0.004 (-0.034, 0.042) | 0.83 | 16,297 | -0.020 (-0.058, 0.018) | 0.32 | 16,297 | -0.024 (-0.062, 0.014) | 0.21 |
| Autistic Traits | Social | 3 | Unadjusted | 26,628 | 0.014 (-0.007, 0.035) | 0.20 | 26,628 | -0.002 (-0.023, 0.020) | 0.88 | 26,628 | 0.006 (-0.016, 0.027) | 0.61 |
| Autistic Traits | Social | 8 | Adjusted for mother and father PRS | 12,382 | 0.030 (-0.031, 0.090) | 0.33 | 12,382 | 0.012 (-0.049, 0.072) | 0.70 | 12,382 | 0.023 (-0.037, 0.084) | 0.45 |
| Autistic Traits | Social | 8 | Unadjusted | 19,872 | 0.019 (-0.015, 0.053) | 0.26 | 19,872 | 0.002 (-0.032, 0.036) | 0.93 | 19,872 | 0.019 (-0.015, 0.053) | 0.27 |
| Autistic Traits | Total | 3 | Adjusted for mother and father PRS | 16,303 | -0.012 (-0.083, 0.059) | 0.74 | 16,303 | -0.043 (-0.115, 0.028) | 0.24 | 16,303 | -0.027 (-0.099, 0.044) | 0.46 |
| Autistic Traits | Total | 3 | Unadjusted | 26,640 | 0.023 (-0.017, 0.063) | 0.27 | 26,640 | -0.046 (-0.086, -0.006) | 0.03 | 26,640 | -0.008 (-0.048, 0.032) | 0.68 |
| Autistic Traits | Total | 8 | Adjusted for mother and father PRS | 12,391 | 0.004 (-0.068, 0.076) | 0.91 | 12,391 | 0.005 (-0.067, 0.076) | 0.90 | 12,391 | 0.042 (-0.029, 0.114) | 0.25 |
| Autistic Traits | Total | 8 | Unadjusted | 19,884 | 0.0004 (-0.040, 0.041) | 0.98 | 19,884 | -0.010 (-0.050, 0.030) | 0.63 | 19,884 | 0.031 (-0.010, 0.071) | 0.14 |

### Table S15. Paternal negative control Analysis - Paternal PGS adjusted for child and maternal PGS

|  | | | | **DHA** | | | **Vitamin D (Manousaki et al.)** | | | **Vitamin D (Revez et al.)** | | |
| --- | --- | --- | --- | --- | --- | --- | --- | --- | --- | --- | --- | --- |
|  | **Domain** | **Age (Years)** | **Adjustment** | **N** | **Beta (95% CI)** | **P-value** | **N** | **Beta (95% CI)** | **P-value** | **N** | **Beta (95% CI)** | **P-value** |
|  | Language | 3 | Adjusted for child and mother PRS | 16,298 | -0.000007 (-0.021, 0.021) | 1.00 | 16,298 | -0.012 (-0.032, 0.009) | 0.28 | 16,298 | -0.001 (-0.022, 0.020) | 0.91 |
|  | Language | 3 | Unadjusted | 17,990 | -0.006 (-0.022, 0.011) | 0.49 | 17,990 | -0.013 (-0.030, 0.003) | 0.10 | 17,990 | 0.0006 (-0.016, 0.017) | 0.94 |
|  | Language | 5 | Adjusted for child and mother PRS | 12,078 | 0.003 (-0.025, 0.031) | 0.82 | 12,078 | -0.005 (-0.033, 0.023) | 0.73 | 12,078 | 0.003 (-0.025, 0.031) | 0.82 |
|  | Language | 5 | Unadjusted | 13,277 | 0.005 (-0.017, 0.027) | 0.66 | 13,277 | 0.005 (-0.017, 0.026) | 0.67 | 13,277 | 0.010 (-0.012, 0.032) | 0.37 |
|  | Motor | 3 | Adjusted for child and mother PRS | 16,255 | -0.000700 (-0.025, 0.024) | 0.96 | 16,255 | 0.005 (-0.020, 0.030) | 0.69 | 16,255 | -0.019 (-0.044, 0.006) | 0.13 |
|  | Motor | 3 | Unadjusted | 17,941 | -0.009 (-0.029, 0.010) | 0.33 | 17,941 | 0.001 (-0.018, 0.020) | 0.91 | 17,941 | -0.019 (-0.038, 0.0004) | 0.05 |
|  | Motor | 5 | Adjusted for child and mother PRS | 12,113 | -0.009 (-0.040, 0.022) | 0.57 | 12,113 | -0.013 (-0.044, 0.018) | 0.41 | 12,113 | -0.008 (-0.039, 0.023) | 0.62 |
|  | Motor | 5 | Unadjusted | 13,315 | 0.000900 (-0.023, 0.025) | 0.94 | 13,315 | -0.009 (-0.033, 0.015) | 0.48 | 13,315 | -0.007 (-0.031, 0.017) | 0.56 |
| ADHD | Hyperactivity | 3 | Adjusted for child and mother PRS | 16,032 | 0.008 (-0.035, 0.051) | 0.71 | 16,032 | 0.004 (-0.039, 0.048) | 0.84 | 16,032 | -0.029 (-0.072, 0.015) | 0.20 |
| ADHD | Hyperactivity | 3 | Unadjusted | 17,697 | -0.005 (-0.039, 0.028) | 0.77 | 17,697 | -0.018 (-0.051, 0.016) | 0.30 | 17,697 | -0.018 (-0.052, 0.015) | 0.29 |
| ADHD | Hyperactivity | 5 | Adjusted for child and mother PRS | 12,025 | -0.008 (-0.043, 0.027) | 0.65 | 12,025 | 0.005 (-0.030, 0.040) | 0.77 | 12,025 | -0.009 (-0.044, 0.027) | 0.63 |
| ADHD | Hyperactivity | 5 | Unadjusted | 13,216 | -0.013 (-0.040, 0.015) | 0.37 | 13,216 | -0.006 (-0.033, 0.022) | 0.68 | 13,216 | -0.010 (-0.038, 0.017) | 0.45 |
| ADHD | Hyperactivity | 8 | Adjusted for child and mother PRS | 12,451 | -0.067 (-0.150, 0.016) | 0.11 | 12,451 | 0.0003 (-0.083, 0.083) | 0.99 | 12,451 | 0.013 (-0.070, 0.096) | 0.76 |
| ADHD | Hyperactivity | 8 | Unadjusted | 13,761 | -0.081 (-0.145, -0.016) | 0.01 | 13,761 | -0.025 (-0.089, 0.040) | 0.45 | 13,761 | 0.046 (-0.018, 0.111) | 0.15 |
| ADHD | Inattention | 3 | Adjusted for child and mother PRS | 16,042 | -0.014 (-0.048, 0.021) | 0.44 | 16,042 | 0.024 (-0.011, 0.059) | 0.17 | 16,042 | -0.015 (-0.049, 0.020) | 0.41 |
| ADHD | Inattention | 3 | Unadjusted | 17,697 | -0.013 (-0.040, 0.014) | 0.34 | 17,697 | -0.0009 (-0.028, 0.026) | 0.95 | 17,697 | -0.007 (-0.034, 0.020) | 0.62 |
| ADHD | Inattention | 5 | Adjusted for child and mother PRS | 12,053 | 0.022 ( 0.002, 0.041) | 0.03 | 12,053 | 0.012 (-0.008, 0.031) | 0.24 | 12,053 | -0.006 (-0.026, 0.013) | 0.53 |
| ADHD | Inattention | 5 | Unadjusted | 13,246 | 0.013 (-0.002, 0.028) | 0.10 | 13,246 | 0.002 (-0.013, 0.018) | 0.75 | 13,246 | 0.0004 (-0.015, 0.016) | 0.96 |
| ADHD | Inattention | 8 | Adjusted for child and mother PRS | 12,455 | -0.015 (-0.102, 0.073) | 0.74 | 12,455 | -0.0004 (-0.088, 0.087) | 0.99 | 12,455 | -0.007 (-0.095, 0.080) | 0.87 |
| ADHD | Inattention | 8 | Unadjusted | 13,765 | -0.039 (-0.107, 0.029) | 0.26 | 13,765 | -0.013 (-0.081, 0.055) | 0.71 | 13,765 | 0.028 (-0.040, 0.096) | 0.42 |
| ADHD | Total | 3 | Adjusted for child and mother PRS | 16,090 | -0.005 (-0.075, 0.064) | 0.88 | 16,090 | 0.027 (-0.042, 0.097) | 0.44 | 16,090 | -0.044 (-0.113, 0.026) | 0.22 |
| ADHD | Total | 3 | Unadjusted | 17,753 | -0.017 (-0.071, 0.037) | 0.54 | 17,753 | -0.018 (-0.072, 0.036) | 0.51 | 17,753 | -0.025 (-0.078, 0.029) | 0.36 |
| ADHD | Total | 5 | Adjusted for child and mother PRS | 12,112 | 0.014 (-0.034, 0.061) | 0.58 | 12,112 | 0.015 (-0.033, 0.063) | 0.54 | 12,112 | -0.016 (-0.064, 0.032) | 0.50 |
| ADHD | Total | 5 | Unadjusted | 13,311 | 0.000800 (-0.036, 0.038) | 0.97 | 13,311 | -0.004 (-0.041, 0.033) | 0.83 | 13,311 | -0.010 (-0.047, 0.027) | 0.58 |
| ADHD | Total | 8 | Adjusted for child and mother PRS | 12,456 | -0.081 (-0.234, 0.071) | 0.30 | 12,456 | -0.0004 (-0.153, 0.153) | 1.00 | 12,456 | 0.008 (-0.145, 0.160) | 0.92 |
| ADHD | Total | 8 | Unadjusted | 13,766 | -0.119 (-0.237, -0.0007) | 0.05 | 13,766 | -0.039 (-0.157, 0.079) | 0.52 | 13,766 | 0.076 (-0.043, 0.194) | 0.21 |
| Autistic Traits | RRB | 3 | Adjusted for child and mother PRS | 16,266 | 0.061 ( 0.013, 0.108) | 0.01 | 16,266 | -0.031 (-0.078, 0.016) | 0.20 | 16,266 | -0.015 (-0.062, 0.033) | 0.55 |
| Autistic Traits | RRB | 3 | Unadjusted | 17,952 | 0.046 ( 0.009, 0.083) | 0.01 | 17,952 | -0.054 (-0.091, -0.017) | 0.004 | 17,952 | -0.021 (-0.058, 0.015) | 0.25 |
| Autistic Traits | RRB | 8 | Adjusted for child and mother PRS | 12,041 | 0.000100 (-0.025, 0.026) | 0.99 | 12,041 | -0.007 (-0.033, 0.018) | 0.58 | 12,041 | -0.007 (-0.033, 0.018) | 0.58 |
| Autistic Traits | RRB | 8 | Unadjusted | 13,308 | -0.010 (-0.029, 0.010) | 0.33 | 13,308 | -0.009 (-0.029, 0.011) | 0.37 | 13,308 | 0.005 (-0.015, 0.025) | 0.61 |
| Autistic Traits | Social | 3 | Adjusted for child and mother PRS | 16,297 | -0.003 (-0.036, 0.030) | 0.86 | 16,297 | -0.016 (-0.049, 0.017) | 0.34 | 16,297 | 0.018 (-0.015, 0.051) | 0.30 |
| Autistic Traits | Social | 3 | Unadjusted | 17,985 | -0.003 (-0.029, 0.023) | 0.80 | 17,985 | -0.017 (-0.043, 0.009) | 0.21 | 17,985 | 0.005 (-0.020, 0.031) | 0.68 |
| Autistic Traits | Social | 8 | Adjusted for child and mother PRS | 12,382 | -0.021 (-0.074, 0.032) | 0.44 | 12,382 | -0.016 (-0.069, 0.037) | 0.56 | 12,382 | 0.0004 (-0.053, 0.053) | 0.99 |
| Autistic Traits | Social | 8 | Unadjusted | 13,690 | -0.002 (-0.043, 0.039) | 0.93 | 13,690 | -0.013 (-0.054, 0.028) | 0.53 | 13,690 | 0.013 (-0.028, 0.054) | 0.52 |
| Autistic Traits | Total | 3 | Adjusted for child and mother PRS | 16,303 | 0.061 (-0.001, 0.124) | 0.05 | 16,303 | -0.058 (-0.120, 0.005) | 0.07 | 16,303 | 0.002 (-0.061, 0.064) | 0.96 |
| Autistic Traits | Total | 3 | Unadjusted | 17,993 | 0.045 (-0.003, 0.094) | 0.07 | 17,993 | -0.080 (-0.129, -0.032) | 0.001 | 17,993 | -0.015 (-0.064, 0.033) | 0.53 |
| Autistic Traits | Total | 8 | Adjusted for child and mother PRS | 12,391 | -0.017 (-0.080, 0.045) | 0.59 | 12,391 | -0.021 (-0.084, 0.042) | 0.51 | 12,391 | -0.0003 (-0.063, 0.063) | 0.99 |
| Autistic Traits | Total | 8 | Unadjusted | 13,700 | -0.009 (-0.058, 0.039) | 0.71 | 13,700 | -0.022 (-0.071, 0.026) | 0.37 | 13,700 | 0.023 (-0.026, 0.071) | 0.35 |

##

## Supplementary Figures

### Supplementary Figure S1. Leave-one-out Analysis for IVW Estimates of Autism Genetic Liability on Vitamin D


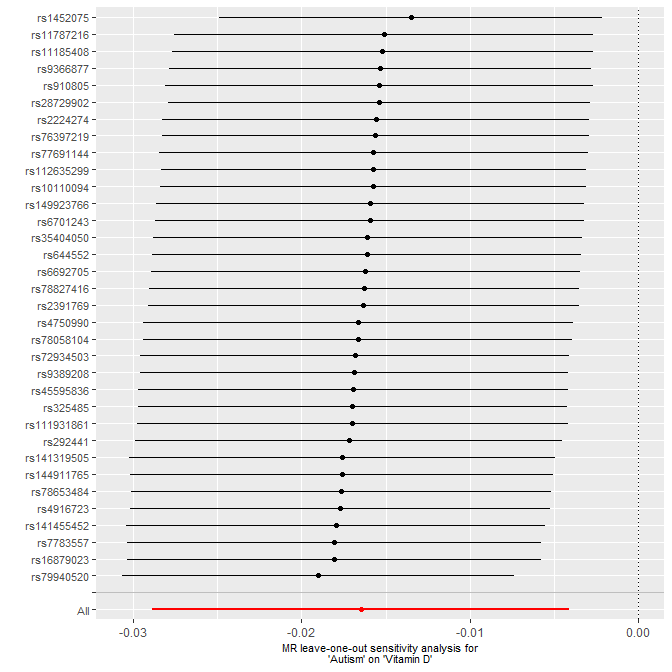


### Supplementary Figure S2. Leave-one-out Analysis for IVW Estimates of ADHD Genetic Liability on DHA levels


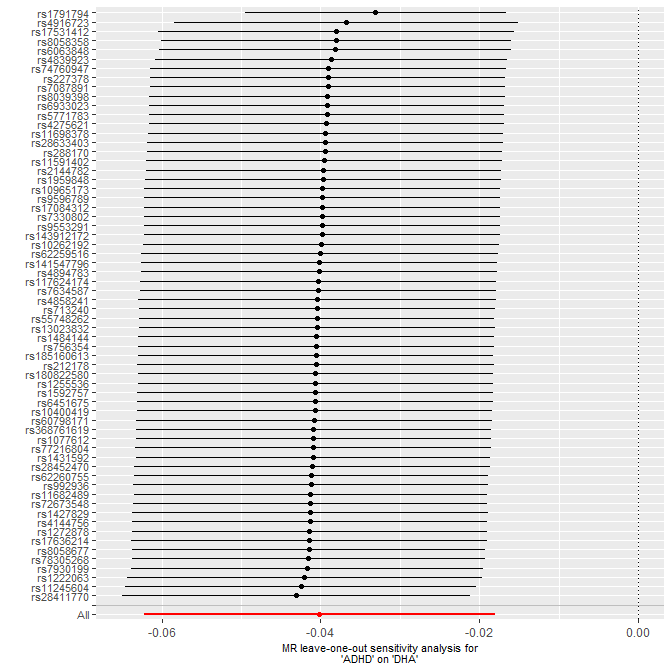


### Supplementary Figure S3. Mother PGS sex split DHA Page


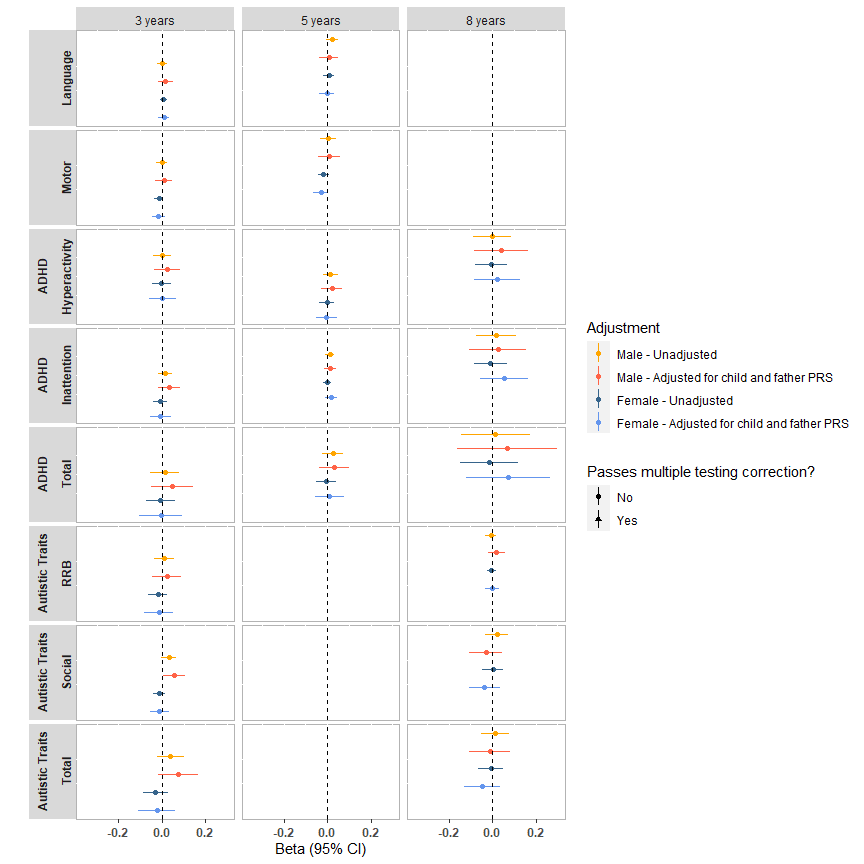


### Supplementary Figure S4. Mother PGS sex split Vitamin D Page


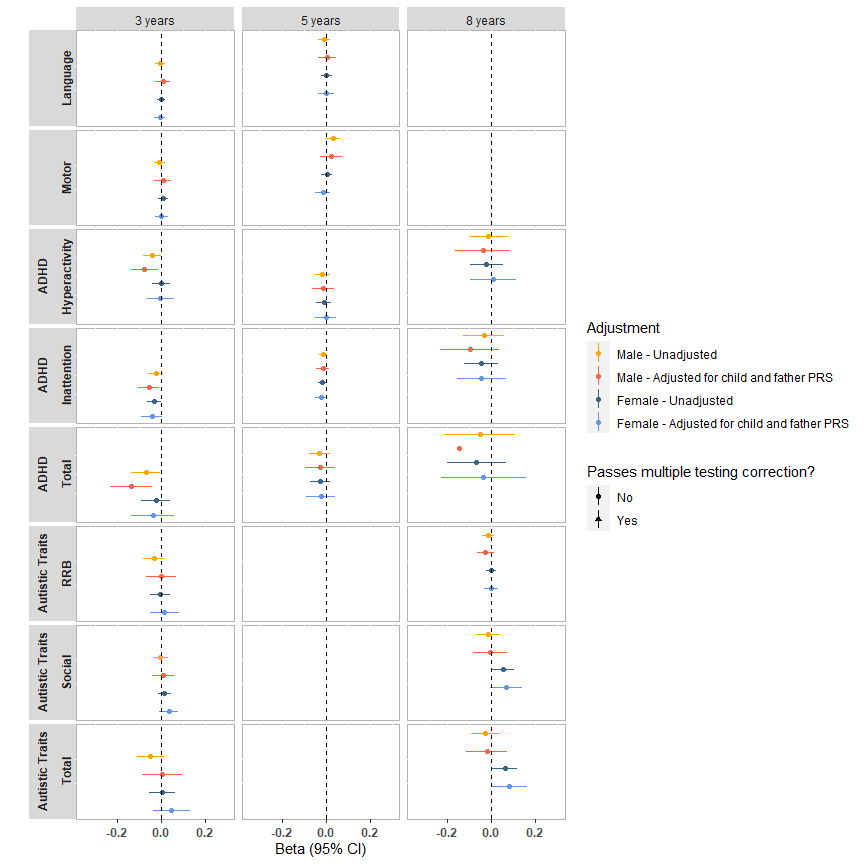


### Supplementary Figure S5. Child PGS DHA Page


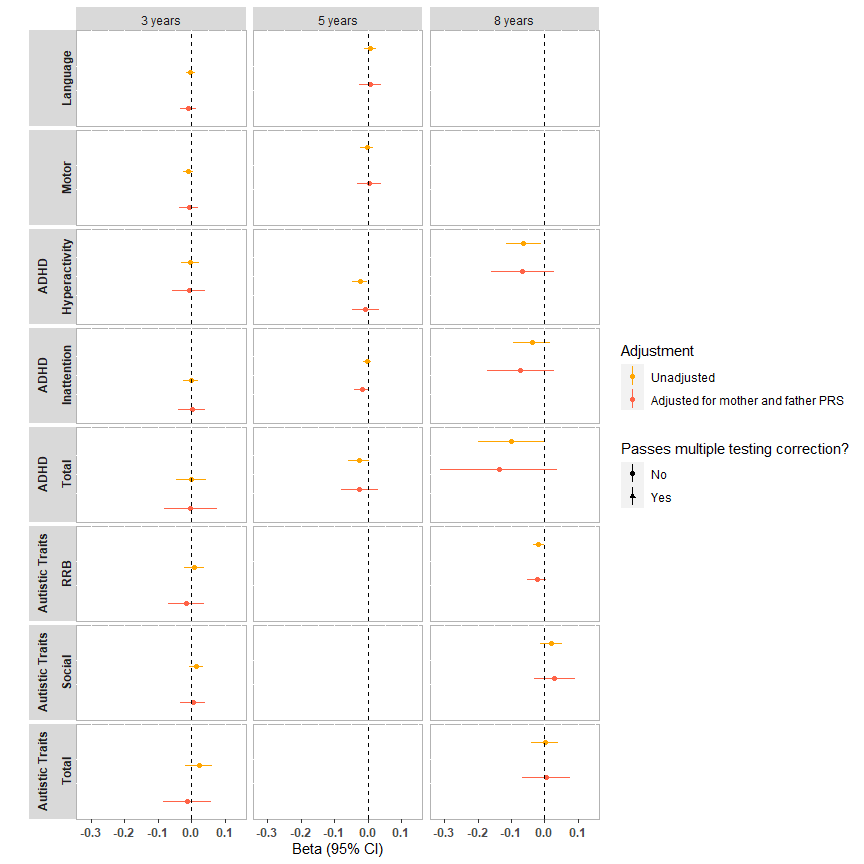


### Supplementary Figure S6. Child PGS Vitamin D Page


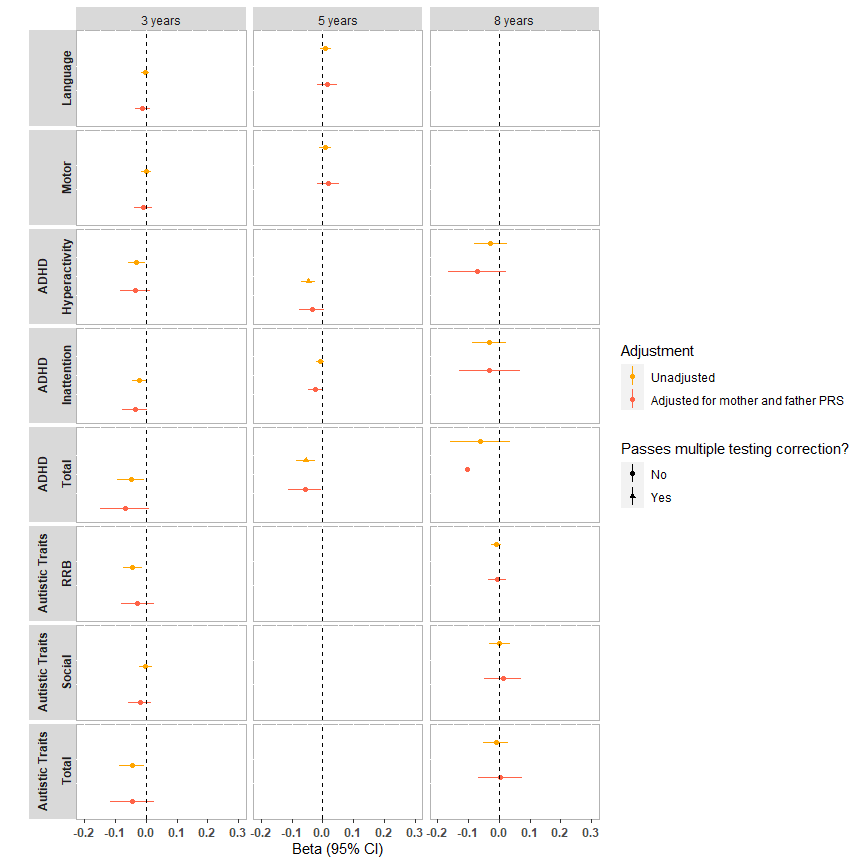


### Supplementary Figure S7. Paternal PGS negative control DHA Page


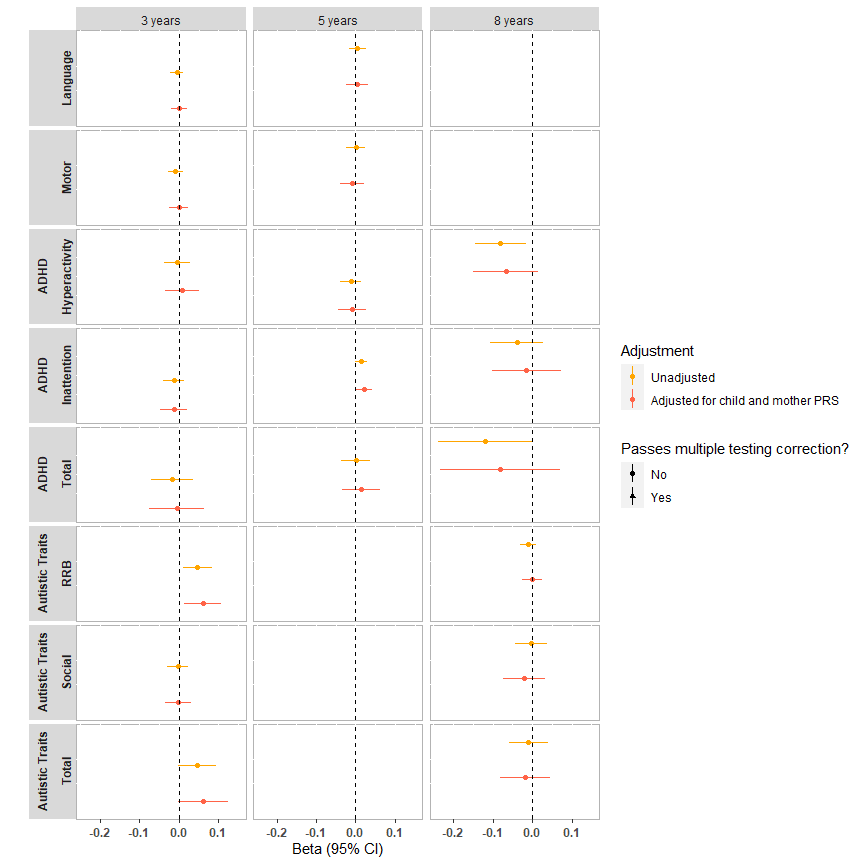


### Supplementary Figure S8. Paternal PGS negative control Vitamin D Page


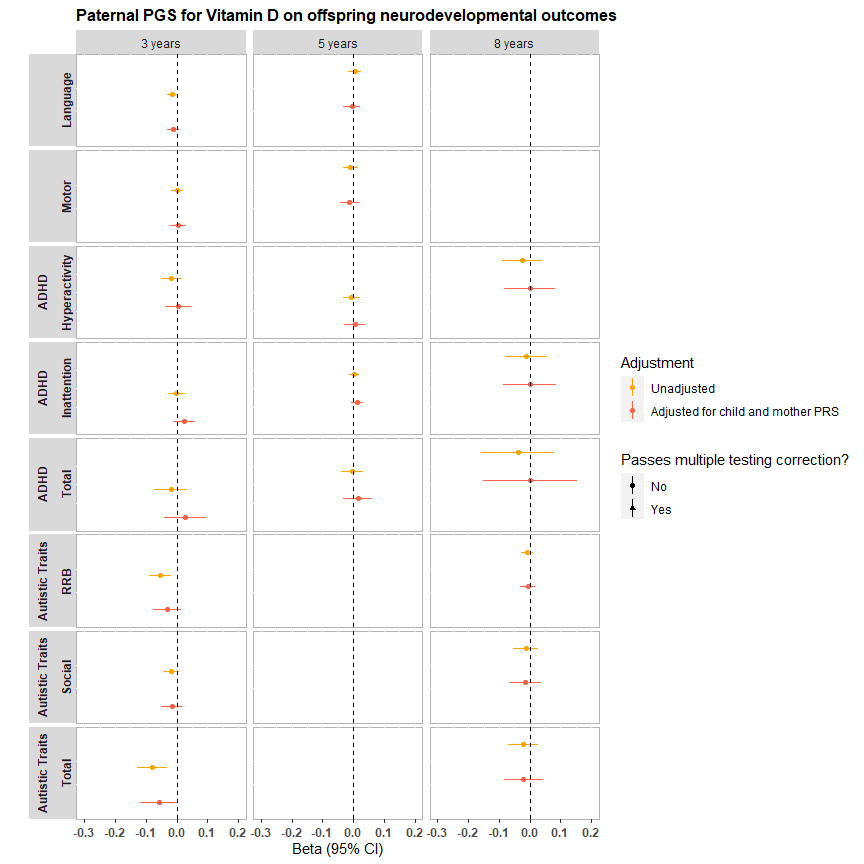


### Supplementary Figure S9. Mendelian randomisation using canonical SNPs for DHA


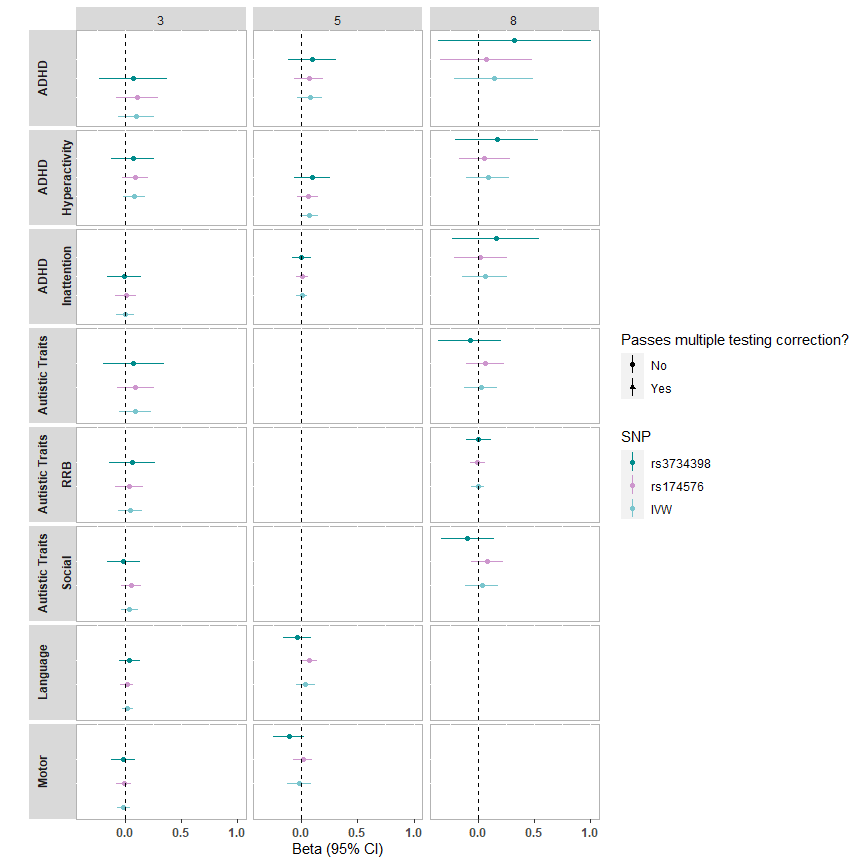


### Supplementary Figure S10. Mendelian randomisation using canonical SNPs for Vitamin D


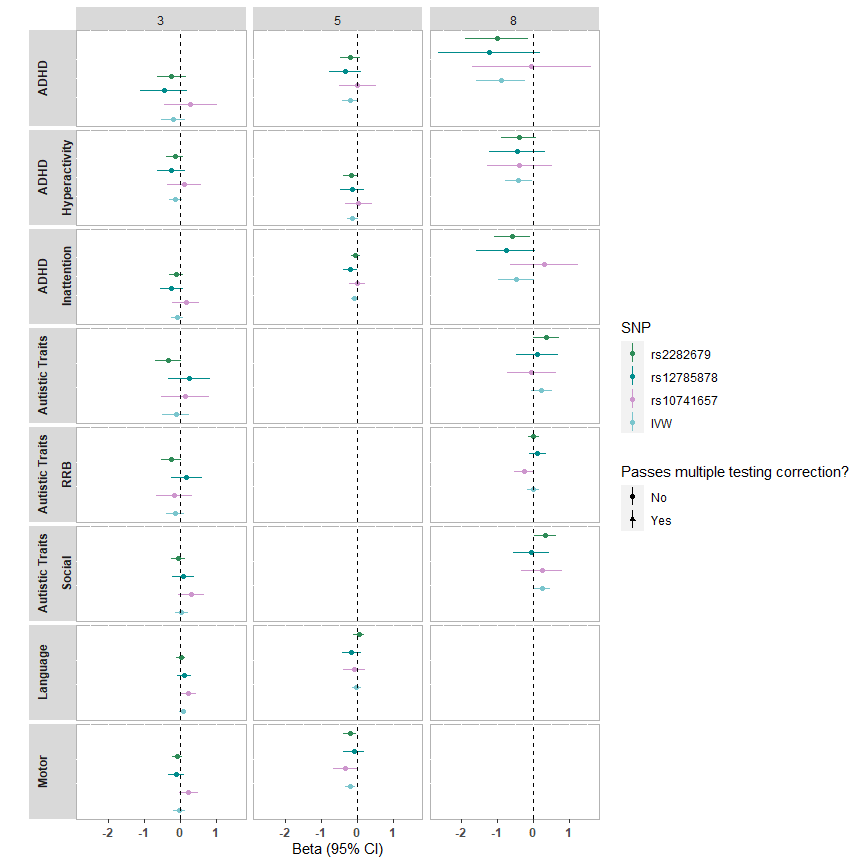


### Supplementary Figure S11. Overview of Analyses Conducted


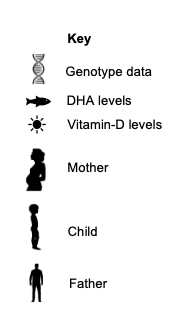


Figure S11. An overview of analyses conducted. Analysis 1 used two-sample Mendelian randomization (MR) to test for possible causal effects of nutrient levels on neurodevelopmental traits. Analysis 2 used trio (mother-father-offspring) data to explore whether associations were specifically due to maternal effects. Analysis 3 used two-sample MR to explore possible reverse causation from neurodevelopmental traits onto nutrient levels. Data sources are depicted: MoBa = results generated using individual level data from the Norwegian Mother Father and Child Cohort Study; IEU Open GWAS Project = genome-wide association study (GWAS) summary statistics conducted in independent samples were downloaded from the IEU Open GWAS Project repository (<https://gwas.mrcieu.ac.uk/>); PGC = GWAS of neurodevelopmental diagnoses were conducted by and downloaded from the Psychiatric Genomics Consortium (PGC). Boxes around genetic instruments mean that it has been controlled for, blocking the path through which it could influence the outcomes.
