# Supplementary material for: Testing maternal effects of vitamin-D and omega-3 levels on offspring neurodevelopmental traits in the Norwegian Mother, Father and Child Cohort Study
Source: Psychol Med. 2024 Sep 9;54(12):3323–33. doi: 10.1017/S0033291724001466 (PMC11496238; doi:10.1017/S0033291724001466)
Supplement: Wootton et al. supplementary material 2 — Wootton et al. supplementary material [file S0033291724001466sup002.docx]

**Supplementary Note**

| Contents |  |
| --- | --- |
| Note 1. Sensitivity analysis using Revez et al (2020) GWAS of Vitamin D | Page 2 |
| Note 2. Validation of genetic instruments for use during pregnancy in the ALSPAC cohort | Page 3 |
|  |  |
| Note 3. Outcome measures of neurodevelopmental traits in the MoBa cohort  Note 4. Sensitivity analysis using canonical SNPs for Vitamin-D and DHA  Note 5. MR Sensitivity analyses and testing the assumption of no horizontal pleiotropy  Note 6. Power Calculation for Mendelian randomisation analyses  References | Page 7  Page 8  Page 9  Page 10  Page 12 |

**Supplementary Note 1. Alternative GWAS for Vitamin D, Revez et al. (2020)**

There were two recent GWAS for Vitamin D which both used similar samples and found similar genome-wide significant SNPs. We have used Manousaki et al. (2020) as our primary GWAS, given that the effect sizes were standardised and therefore more easily interpretable, and given that this instrument has been validated for use in pregnancy in the ALSPAC cohort. However, we have also used the alternative Revez et al. (2020) GWAS as a sensitivity analysis, described here.

Methods

Revez et al. (2020) GWAS of Vitamin D levels comprised 417,580 individuals of European ancestry and identified 143 genome-wide significant independent loci. We did not restrict for independence before restricting to SNPs that passed QC in the MoBa cohort, and then we clumped for independence at r^2^<0.001, using a clumping window of kb=10000. Genetic variants could influence Vitamin D levels through changes to both consumption and metabolism. The genome-wide significant SNPs explained 7.4% of the variance in Vitamin D levels in the QIMR cohort and 3.6% of the variance in the UK Biobank cohort (Revez et al., 2020). However, there was no out of sample prediction estimates available for pregnancy cohorts.

Results

We found consistent results using both Vitamin D GWAS. There was no evidence for an intrauterine effect of maternal vitamin D on any of the neurodevelopmental outcomes in MoBa (Supplementary Table S1). There was consistently no evidence of an association, but there was difference in the effect size, given that the Manousaki et al (2020) GWAS gave the effect sizes in standardised units and the Revez et al. (2020) GWAS gave the units in nmol L^−1^. The genetic instrument using the Revez et al. (2020) was suitably strong with F statistic greater than 10 (Supplementary Table S2) and although these results also showed evidence of heterogeneity (Supplementary Table S3), there was no evidence of bias from horizontal pleiotropy, as measured by the MR Egger intercept (Supplementary Table S4), which I^2^_GX_ suggested was valid (Supplementary Table S2).

When conducting the trio polygenic score analysis with the Revez et al. (2020) GWAS, again the results were highly consistent with the Manousaki et al (2020) GWAS, showing no evidence for intrauterine effects either before or after adjustment for child and father PGS (Supplementary Table S11).

Given the high degree of consistency between the results using the Manousaki et al. (2020) GWAS compared to the Revez et al. (2020) GWAS, we chose to use Manousaki et al. (2020) as our primary analysis, given that the effect estimates are standardised and therefore easier to interpret and compare with standardised DHA effects. Furthermore, the Manousaki et al. (2020) genetic instrument had been shown to associated with levels of vitamin D during pregnancy in the ALSPAC cohort (Madley-Dowd et al., 2022). We are encouraged that the results are so consistent, which is expected given the highly overlapping GWAS samples.

**Supplementary Note 2. Validation of genetic instruments for use during pregnancy in the ALSPAC cohort**

**Methods**

Sample

The Avon Longitudinal Study of Parents and Children (ALSPAC) recruited Pregnant women resident in Avon, UK with expected dates of delivery 1st April 1991 to 31st December 1992 were invited to take part in the study(Boyd et al., 2013; Fraser et al., 2012). The initial number of pregnancies enrolled is 14,541 (for these at least one questionnaire has been returned or a “Children in Focus” clinic had been attended by 19/07/99). Of these initial pregnancies, there was a total of 14,676 foetuses, resulting in 14,062 live births and 13,988 children who were alive at 1 year of age. When the oldest children were approximately 7 years of age, an attempt was made to bolster the initial sample with eligible cases who had failed to join the study originally. As a result, when considering variables collected from the age of seven onwards (and potentially abstracted from obstetric notes) there are data available for more than the 14,541 pregnancies mentioned above. The number of new pregnancies not in the initial sample (known as Phase I enrolment) that are currently represented on the built files and reflecting enrolment status at the age of 24 is 906, resulting in an additional 913 children being enrolled (456, 262 and 195 recruited during Phases II, III and IV respectively), resulting in an additional 913 children being enrolled. The phases of enrolment are described in more detail in the cohort profile paper and its update (see footnote 4 below). The total sample size for analyses using any data collected after the age of seven is therefore 15,447 pregnancies, resulting in 15,658 foetuses. Of these 14,901 children were alive at 1 year of age. A 10% sample of the ALSPAC cohort, known as the Children in Focus (CiF) group, attended clinics at the University of Bristol at various time intervals between 4 to 61 months of age. The CiF group were chosen at random from the last 6 months of ALSPAC births (1432 families attended at least one clinic). Excluded were those mothers who had moved out of the area or were lost to follow-up, and those partaking in another study of infant development in Avon. Ethical approval for the study was obtained from the ALSPAC Ethics and Law Committee and the Local Research Ethics Committees. Consent for biological samples has been collected in accordance with the Human Tissue Act (2004). Please note that the study website contains details of all the data that is available through a fully searchable data dictionary and variable search tool (<http://www.bristol.ac.uk/alspac/researchers/our-data/>).

Genetic variants for DHA

Genotyping and QC in ALSPAC is described in detail elsewhere (Fraser et al., 2012). Of the 38 genome-wide significant SNPs associated with DHA levels that were available in the MoBa cohort, 34 were available in the ALSPAC cohort (Table N1). The two genetic variants of known biological function (rs174576, rs3734398) (described in Supplementary Note S4) were also extracted for validation. All genetic variants were extracted using PLINK (Purcell et al., 2007). Allele counts were tested for association with DHA. For the 34 genetic instruments used in the main analysis, we also created a polygenic score, weighting by the effect sizes from the IEU GWAS meta-analysis (Elsworth et al., 2020). Variants of known function were aligned to count decreasing alleles.

Genetic variants for vitamin-D

Genetic variants for vitamin-D identified in (Manousaki et al., 2020) have been validated for use during pregnancy in the ALSPAC cohort previously (Madley-Dowd et al., 2022). We extracted the four genetic variants of known function (rs2282679, rs12785878, rs10741657, rs6013897) described in further detail in Supplementary Note S4. Variants of known function were aligned to count decreasing alleles.

Measures of DHA during pregnancy

Levels of DHA (226:3) from blood samples were taken during pregnancy. Mothers were not required to fast. We combined measures taken across any stage of pregnancy.

Measures of vitamin-D during pregnancy

Levels of vitamin-D during pregnancy in ALSPAC are described elsewhere (Madley-Dowd et al., 2022). We used the same phenotype derivation from blood samples across pregnancy with seasonal adjustment (Madley-Dowd et al., 2022).

**Results**

Validation of genome-wide significant DHA variants

Genotype data and measures of DHA during pregnancy were available for 5,156 mothers. Mean DHA levels were 2.34 (SD = 1.32). The PGS for DHA explained 0.85% of the variance in DHA levels during pregnancy (Table N1 and Figure N1). Twelve mothers had DHA levels of 0. We conducted a sensitivity analysis where these individuals were excluded. Results were highly consistent, with the DHA PGS explaining 0.81% of the variance in DHA levels during pregnancy.

**Table N1.** Association between the DHA levels PGS and SNPs with DHA levels during pregnancy in the ALSPAC cohort

| **Exposure** | **Beta** | **Lower 95% CI** | **Upper 95% CI** | **P-value** | **r^2^** | **N** |
| --- | --- | --- | --- | --- | --- | --- |
| DHA PGS | 20.682 | 8.256 | 33.109 | 0.001 | 0.0085 | 3644 |
| DHA PGS (std) | 0.073 | 0.029 | 0.116 | 0.001 | 0.0085 | 3644 |
| rs638714_g | 0.027 | -0.037 | 0.092 | 0.403 | 0.0058 | 3642 |
| rs660240_c | -0.009 | -0.086 | 0.068 | 0.820 | 0.0056 | 3633 |
| rs11122450_g | -0.016 | -0.079 | 0.047 | 0.622 | 0.0056 | 3634 |
| rs34722314_t | 0.103 | 0.012 | 0.194 | 0.027 | 0.0069 | 3609 |
| rs1260326_t | 0.003 | -0.060 | 0.066 | 0.927 | 0.0055 | 3644 |
| rs309134_a | 0.009 | -0.067 | 0.084 | 0.818 | 0.0055 | 3639 |
| rs273912_t | -0.002 | -0.071 | 0.066 | 0.945 | 0.0055 | 3644 |
| rs6931604_t | 0.001 | -0.063 | 0.064 | 0.985 | 0.0053 | 3596 |
| rs112248289_a | -0.096 | -0.191 | -0.002 | 0.045 | 0.0066 | 3540 |
| rs14415_t | -0.025 | -0.093 | 0.043 | 0.469 | 0.0056 | 3637 |
| rs9987289_g | -0.020 | -0.126 | 0.087 | 0.717 | 0.0055 | 3628 |
| rs145391587_c | -0.022 | -0.124 | 0.079 | 0.669 | 0.0058 | 3633 |
| rs2954021_a | -0.014 | -0.077 | 0.048 | 0.650 | 0.0056 | 3644 |
| rs1800978_c | 0.064 | -0.030 | 0.157 | 0.183 | 0.0058 | 3626 |
| rs2666761_g | 0.040 | -0.029 | 0.109 | 0.253 | 0.0058 | 3617 |
| rs7924036_t | 0.020 | -0.042 | 0.082 | 0.522 | 0.0057 | 3644 |
| rs61886778_a | 0.073 | -0.005 | 0.150 | 0.065 | 0.0066 | 3635 |
| rs2238001_c | -0.011 | -0.112 | 0.090 | 0.834 | 0.0057 | 3536 |
| rs174529_t | 0.098 | 0.033 | 0.163 | 0.003 | 0.0082 | 3522 |
| rs2736595_g | 0.023 | -0.045 | 0.091 | 0.510 | 0.0062 | 3598 |
| rs72997616_c | 0.074 | -0.033 | 0.182 | 0.175 | 0.0061 | 3592 |
| rs613808_a | -0.064 | -0.137 | 0.008 | 0.083 | 0.0070 | 3273 |
| rs139974673_c | -0.017 | -0.208 | 0.175 | 0.865 | 0.0056 | 3623 |
| rs1560390_t | 0.006 | -0.068 | 0.081 | 0.867 | 0.0056 | 3636 |
| rs261291_c | -0.010 | -0.075 | 0.056 | 0.769 | 0.0055 | 3563 |
| rs72789541_t | 0.016 | -0.054 | 0.086 | 0.656 | 0.0062 | 3464 |
| rs183130_t | 0.062 | -0.007 | 0.131 | 0.077 | 0.0068 | 3282 |
| rs72836561_c | 0.034 | -0.175 | 0.243 | 0.748 | 0.0052 | 3536 |
| rs77960347_g | 0.173 | -0.079 | 0.425 | 0.178 | 0.0061 | 3622 |
| rs9304381_t | -0.018 | -0.101 | 0.066 | 0.679 | 0.0057 | 3617 |
| rs142158911_g | 0.061 | -0.042 | 0.163 | 0.246 | 0.0061 | 3552 |
| rs2278426_c | -0.031 | -0.204 | 0.143 | 0.728 | 0.0056 | 3644 |
| rs58542926_c | 0.061 | -0.053 | 0.176 | 0.296 | 0.0059 | 3638 |
| rs7412_c | 0.032 | -0.082 | 0.145 | 0.584 | 0.0052 | 3512 |


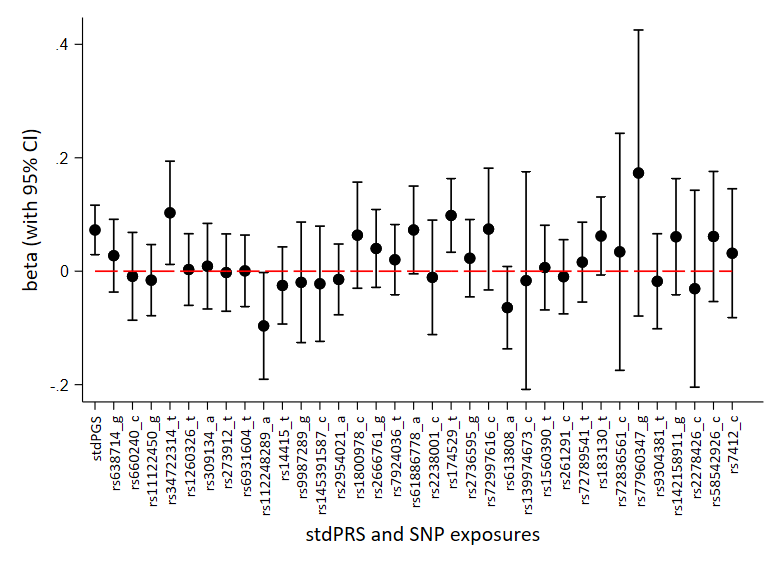


*Figure N1. The association of the standardised polygenic score (stdPGS) and each of the individual SNP effects on levels of DHA during pregnancy in the ALSPAC cohort.*

Validation of DHA variants with known function

Effect estimates for both genetic variants was in the expected decreasing direction, but effects were only significant for rs174576 (Figure N2).


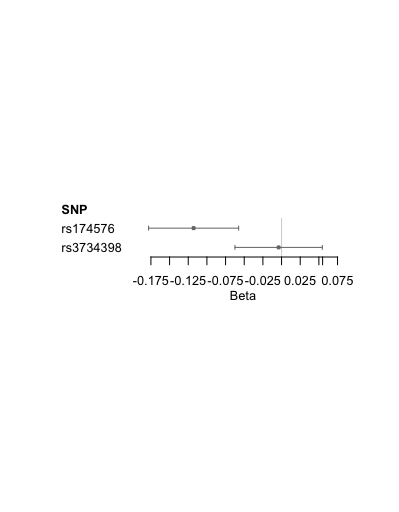


*Figure N2. The association of the variants with known function on levels of DHA during pregnancy in the ALSPAC cohort.*

Validation of Vitamin-D variants with known function

Effect estimates for all genetic variants with known function was in the expected decreasing direction, but effects were only significant for rs12785878 and rs2282679 (Figure N3).


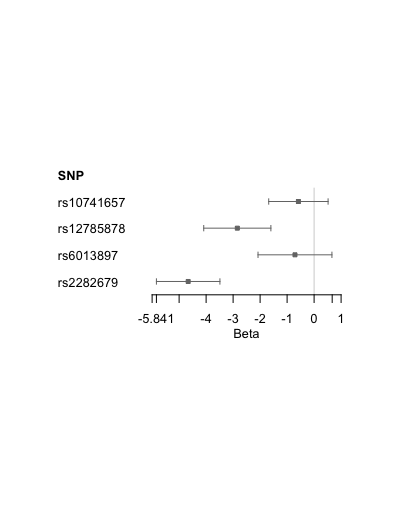


*Figure N3. The association of the variants with known function on levels of vitamin-D during pregnancy in the ALSPAC cohort.*

**Note 3. Outcome measures of neurodevelopmental traits in the MoBa cohort**

We focused on clinically relevant measures of language delay, motor delay, autistic traits (social communication and restricted, inflexible behaviours) and ADHD traits (inattention and hyperactivity), which were available self-reported by the mothers at child ages 3, 5 and 8 years.

*ADHD traits* were assessed using two scales: the Child-Behaviour Checklist (CBCL) at 3 and 5 years (Achenbach & Edelbrock, 1983), and the Disruptive Behaviour Disorder Rating Scale (RS-DBD) (Silva et al., 2005) at 8 years. A reduced version of the CBCL was used, comprising 3 inattention items and 2 hyperactivity items at age 3 and comprising 2 inattention items and 4 hyperactivity items at age 5. Responses were on a three-point scale rated 1- Rarely/never, 2- Sometimes and 3- Often/typical (Achenbach & Edelbrock, 1983). At age 3, CBCL items were supplemented with additional items from the DSM (4^th^ edition) giving a total of 5 inattention items and 6 for hyperactivity (American Psychiatric Association, 1980). The RS-DBD at age 8 consisted of 18 items related to ADHD (9 hyperactivity, 9 inattention). Each item is rated on a four-point scale from 1-never/rarely to 4-very often (Silva et al., 2005).

*Autism traits* were assessed using the Social Communication Questionnaire (SCQ) for language (7 items), social-communication deficits (22 items) and repetitive behaviours (11 items) at 3 and 8 years (Rutter et al., 2003). The SCQ is a 40-item questionnaire with yes/no responses. The first item determines whether or not the child is verbal, and the remaining 39 items are used to calculate total score. A total score above 15 indicates that the child is likely to be on the Autism Spectrum.

*Language delay* was measured using the Ages and Stages Questionnaire (ASQ) language items at ages 3 and 5 years (Squires et al., 1995). The language scale included 6 items at age 3 years and 7 items at age 5 years rated as 1-Yes, 2-Sometimes, 3-Not yet.

*Motor delay* was measured by the ASQ at age 3 (4 items rated as 1-Yes, 2-Sometimes, 3-Not yet) and by the Child Development Inventory at age 5 years (12-items rated as yes/no). These 12 items are from the fine motor skills and the gross motor skills subscales.

**Note S4. Sensitivity analysis using canonical SNPs for Vitamin-D and DHA**

Methods

*DHA.* We have used two SNPs. The first is rs174576 which is known to be inside the *FADS2* gene which codes for the Delta 6 desaturase enzyme, involved in the metabolism of DHA. The second is rs3734398 in the *ELOVL2* gene which codes for the fatty acid elongase 2 enzyme also involved in the metabolism of DHA. Both of these SNPs have been validated for use in predicting levels of DHA in individuals of European ancestry (Borges et al., 2022).

*Vitamin-D.* The SUNLIGHT consortium identified four genome-wide significant SNPs with a direct biological role in the levels of Vitamin-D (Mokry et al., 2015). These were: 1) rs2282679 in the gene *GC,* which encodes the vitamin-D binding protein (DBP). DBP is the primary protein transporter for vitamin-D, carrying 80%–90% of vitamin-D to the target organs. 2) rs12785878 near the *DHCR7* gene. The product of the *DHCR7* gene converts 7-dehydrocholesterol to cholesterol, providing a substrate for vitamin-D production. 3) rs10741657 near the *CYP2R1* gene, which regulates vitamin-D synthesis through 25-hydroxylation of vitamin-D in the liver. 4) rs6013897 in the gene *CYP24A1,* which inactivates the active form of vitamin-D.

We searched for each of these SNPs in the MoBa sample. Where the specific genetic variant was not available, we searched for suitable proxies at r^2^>0.8. All SNPs were available and passed QC with the exception of rs6013897, for which no suitable proxies could be found. Given that only individual SNPs are available for analysis, we conducted Wald Ratios for each SNP individually and conducted an inverse-variance weighted meta-analysis.

Results

For DHA, neither of the canonical SNPs were associated with any of the neurodevelopmental outcomes (see Figure S9). For vitamin-D canonical SNPs, there was some weak evidence for an association between rs2282679 alleles and inattention at age 5 years, as well as total ADHD symptoms and inattention at age 8 years (see Figure S10). These associations are weak, but they are less likely to be biased by parenting or lifestyle factors and more likely to suggest true causal effects of vitamin-D levels.

**Note 5. MR Sensitivity analyses and testing the assumption of no horizontal pleiotropy**

Assumptions 2 and 3 of Mendelian randomisation can be violated by the presence of horizontal pleiotropy – when a genetic instrument for the exposure is associated with the outcome through pathways other than via the exposure. We conducted 3 additional sensitivity methods with different assumptions about the nature of possible pleiotropic effects. First, the weighted median method assumes at least 50% of the total weight of the instrument comes from valid variants (Bowden et al., 2016). Second, MR Egger which allows all genetic instruments to violate MR assumption 3 but makes the additional Instrument Strength Independent of Direct Effect (InSIDE) assumption. This assumption states that the association between the genetic instrument and exposure should not be correlated with path from the genetic instrument to the outcome that is independent of the exposure of interest (Bowden et al., 2015). Third, we used the weighted mode method, which assumes the most common causal effect is consistent with the true causal effect (Hartwig et al., 2017).

Given the different assumptions made by each method about the nature of pleiotropy, we would consider the strongest evidence of a causal effect to occur when there is evidence for an effect in the IVW analysis, and the three sensitivity methods find the same direction of effect with relatively consistent effect sizes. Results for MR Egger and weighted mode in particular tend to be less precise given that these methods require additional power.

**Supplementary Note 6. Power Calculation**

We assessed a range of genetic instruments for micronutrients which have previously been hypothesised to be associated with neurodevelopmental outcomes in children. To be suitable for MR analysis, we required that they have:

- At least 10 genome-wide significant SNPs
- Explained at least 3% of the variance in an independent sample.
- That full summary statistics were publicly available.
- Ideally, that it was measured during pregnancy in the ALSPAC cohort so that we could assess the suitability of the instrument for predicting nutrient levels in pregnant women.

Each of the nutrients that we explored are given in Table N1. Vitamin D and DHA were the only nutrients which met all of the inclusion criteria.

**Table N2.** Overview of nutritional biomarkers with their respective genome-wide association studies (GWAS) and number of genetic variants to proxy for the respective nutrient

| **Nutrient** | **GWAS Study** | **N_SNP_** | **% R2** | **Variance prediction sample** | **Full sumstats available** | **Measured in ALSPAC during pregnancy** |
| --- | --- | --- | --- | --- | --- | --- |
| Fasting Glucose | (Scott et al., 2012) | 36 | 4.8% | Independent sample | Yes (at IEU Open GWAS) | Not in pregnancy |
| DHA (Omega 3) | IEU Open GWAS | 32 | 6.6% | Calculated from summary stats | Yes (at IEU Open GWAS) | Yes |
| Vitamin B12 | (Grarup et al., 2013) | 11 | 6.3% | Calculated from summary stats | Not obviously | Yes – diet diaries |
| Vitamin B12 | IEU Open GWAS (UKB only) | 0 | - | - | Yes (at IEU Open GWAS) | Yes – diet diaries |
| Vitamin D | (Manousaki et al., 2020) | 70 | 3.1% | Independent sample | Yes | Yes |
| Vitamin D | (Revez et al., 2020) | 111 | 3.4% | Calculated from summary stats | Yes | Yes |
| Folate | (Grarup et al., 2013) | 4 | 1.0% | Calculated from summary stats | Not obviously | Yes – diet diaries |
| Folate | IEU Open GWAS (UKB only) | 1 | - | - | Yes (at IEU Open GWAS) | Yes – diet diaries |
| Iron | (Benyamin et al., 2014) | 11 | 3.4% | Independent sample | Yes (at IEU Open GWAS) | Yes – diet diaries |
| Iron | IEU Open GWAS (UKB only) | 0 | - | - | Yes (at IEU Open GWAS) | Yes – diet diaries |
| Calcium | (O’Seaghdha et al., 2013) | 7 | 8.4%? | Calculated from summary stats | Not obviously | Yes |
| Calcium | IEU Open GWAS (UKB only) | 0 | - | - | Yes (at IEU Open GWAS) | Yes |
| Glutamine | IEU Open GWAS | 47 | 4.9% | Calculated from summary stats | Yes (at IEU Open GWAS) | Not in pregnancy |

Note. N_SNP_ refers to the number of independent genome-wide significant SNPs

Based upon these estimates, we conducted power calculations using the mRnd power calculator (Brion et al., 2013). We estimated that we would have 80% power to detect a standardized beta effect size of 0.092 for DHA and 0.133 for vitamin D (Table N3).

**Table N3.** Estimated effect size we would have 80% power to detect.

|  | **GWAS** | **N SNPs** | **Variance explained in adults** | **Age 3 N=26,000 Minimum effect 80%** | **Age 5/8 N=18,000 Minimum effect 80%** |
| --- | --- | --- | --- | --- | --- |
| Vitamin D | Manousaki et al. (2020) | 51 | 3.1% | Beta = 0.10 | Beta= 0.12 |
| DHA | IEU GWAS | 32 | 6.6% | Beta = 0.07 | Beta = 0.08 |
|  | **GWAS** | **N SNPs** | **Variance explained in pregnancy** | **Age 3 N=26,000 Minimum effect 80%** | **Age 5/8 N=18,000 Minimum effect 80%** |
| Vitamin D | Manousaki et al. (2020) | 51 | 1.35% (Madley-Dowd et al., 2022) | Beta = 0.15 | Beta = 0.18 |
| DHA | IEU GWAS | 32 | 0.85% (see Note 2) | Beta = 0.19 | Beta = 0.23 |

These estimates were calculated using the mRnd power calculator for Mendelian randomisation (Brion et al., 2013).

**References**

Achenbach, T. M., & Edelbrock, C. S. (1983). *Manual for the child behaviour checklist and revised child behaviour profile.* 393–405.

American Psychiatric Association, A. (1980). *Diagnostic and statistical manual of mental disorders* (Vol. 3). American Psychiatric Association Washington, DC.

Benyamin, B., Esko, T., Ried, J. S., Radhakrishnan, A., Vermeulen, S. H., Traglia, M., Gögele, M., Anderson, D., Broer, L., & Podmore, C. (2014). Novel loci affecting iron homeostasis and their effects in individuals at risk for hemochromatosis. *Nature Communications*, *5*(1), 1–11.

Borges, M.-C., Haycock, P., Zheng, J., Hemani, G., Howe, L. J., Schmidt, A. F., Staley, J. R., Lumbers, R. T., Henry, A., Lemaitre, R. N., Gaunt, T. R., Holmes, M. V., Davey Smith, G., Hingorani, A. D., & Lawlor, D. A. (2022). The impact of fatty acids biosynthesis on the risk of cardiovascular diseases in Europeans and East Asians: A Mendelian randomization study. *Human Molecular Genetics*, *31*(23), 4034–4054. https://doi.org/10.1093/hmg/ddac153

Bowden, J., Davey Smith, G., & Burgess, S. (2015). Mendelian randomization with invalid instruments: Effect estimation and bias detection through Egger regression. *International Journal of Epidemiology*, *44*(2), 512–525.

Bowden, J., Davey Smith, G., Haycock, P. C., & Burgess, S. (2016). Consistent estimation in Mendelian randomization with some invalid instruments using a weighted median estimator. *Genetic Epidemiology*, *40*(4), 304–314.

Boyd, A., Golding, J., Macleod, J., Lawlor, D. A., Fraser, A., Henderson, J., Molloy, L., Ness, A., Ring, S., & Davey Smith, G. (2013). Cohort profile: The ‘children of the 90s’—the index offspring of the Avon Longitudinal Study of Parents and Children. *International Journal of Epidemiology*, *42*(1), 111–127.

Brion, M.-J. A., Shakhbazov, K., & Visscher, P. M. (2013). Calculating statistical power in Mendelian randomization studies. *International Journal of Epidemiology*, *42*(5), 1497–1501. https://doi.org/10.1093/ije/dyt179

Elsworth, B., Lyon, M., Alexander, T., Liu, Y., Matthews, P., Hallett, J., Bates, P., Palmer, T., Haberland, V., Davey Smith, G., Zheng, J., Haycock, P., Gaunt, T. R., & Hemani, G. (2020). The MRC IEU OpenGWAS data infrastructure. *bioRxiv*, 2020.08.10.244293. https://doi.org/10.1101/2020.08.10.244293

Fraser, A., Macdonald-Wallis, C., Tilling, K., Boyd, A., Golding, J., Davey Smith, G., Henderson, J., Macleod, J., Molloy, L., & Ness, A. (2012). Cohort profile: The Avon Longitudinal Study of Parents and Children: ALSPAC mothers cohort. *International Journal of Epidemiology*, *42*(1), 97–110.

Grarup, N., Sulem, P., Sandholt, C. H., Thorleifsson, G., Ahluwalia, T. S., Steinthorsdottir, V., Bjarnason, H., Gudbjartsson, D. F., Magnusson, O. T., Sparsø, T., Albrechtsen, A., Kong, A., Masson, G., Tian, G., Cao, H., Nie, C., Kristiansen, K., Husemoen, L. L., Thuesen, B., … Pedersen, O. (2013). Genetic Architecture of Vitamin B12 and Folate Levels Uncovered Applying Deeply Sequenced Large Datasets. *PLOS Genetics*, *9*(6), e1003530. https://doi.org/10.1371/journal.pgen.1003530

Hartwig, F. P., Davey Smith, G., & Bowden, J. (2017). Robust inference in summary data Mendelian randomization via the zero modal pleiotropy assumption. *International Journal of Epidemiology*, *46*(6), 1985–1998. https://doi.org/10.1093/ije/dyx102

Madley-Dowd, P., Dardani, C., Wootton, R. E., Dack, K., Palmer, T., Thurston, R., Havdahl, A., Golding, J., Lawlor, D., & Rai, D. (2022). Maternal vitamin D during pregnancy and offspring autism and autism-associated traits: A prospective cohort study. *Molecular Autism*, *13*(1), 44. https://doi.org/10.1186/s13229-022-00523-4

Manousaki, D., Mitchell, R., Dudding, T., Haworth, S., Harroud, A., Forgetta, V., Shah, R. L., Luan, J., Langenberg, C., Timpson, N. J., & Richards, J. B. (2020). Genome-wide Association Study for Vitamin D Levels Reveals 69 Independent Loci. *American Journal of Human Genetics*, *106*(3), 327–337. https://doi.org/10.1016/j.ajhg.2020.01.017

Mokry, L. E., Ross, S., Ahmad, O. S., Forgetta, V., Smith, G. D., Goltzman, D., Leong, A., Greenwood, C. M. T., Thanassoulis, G., & Richards, J. B. (2015). Vitamin D and Risk of Multiple Sclerosis: A Mendelian Randomization Study. *PLoS Medicine*, *12*(8), e1001866. https://doi.org/10.1371/journal.pmed.1001866

O’Seaghdha, C. M., Wu, H., Yang, Q., Kapur, K., Guessous, I., Zuber, A. M., Köttgen, A., Stoudmann, C., Teumer, A., Kutalik, Z., Mangino, M., Dehghan, A., Zhang, W., Eiriksdottir, G., Li, G., Tanaka, T., Portas, L., Lopez, L. M., Hayward, C., … Bochud, M. (2013). Meta-analysis of genome-wide association studies identifies six new Loci for serum calcium concentrations. *PLoS Genetics*, *9*(9), e1003796. https://doi.org/10.1371/journal.pgen.1003796

Purcell, S., Neale, B., Todd-Brown, K., Thomas, L., Ferreira, M. A., Bender, D., Maller, J., Sklar, P., De Bakker, P. I., Daly, M. J., & others. (2007). PLINK: A tool set for whole-genome association and population-based linkage analyses. *The American Journal of Human Genetics*, *81*(3), 559–575.

Revez, J. A., Lin, T., Qiao, Z., Xue, A., Holtz, Y., Zhu, Z., Zeng, J., Wang, H., Sidorenko, J., Kemper, K. E., Vinkhuyzen, A. A. E., Frater, J., Eyles, D., Burne, T. H. J., Mitchell, B., Martin, N. G., Zhu, G., Visscher, P. M., Yang, J., … McGrath, J. J. (2020). Genome-wide association study identifies 143 loci associated with 25 hydroxyvitamin D concentration. *Nature Communications*, *11*(1), 1647. https://doi.org/10.1038/s41467-020-15421-7

Rutter, M., Bailey, A., & Lord, C. (2003). SCQ. *The Social Communication Questionnaire. Torrance, CA: Western Psychological Services*. http://conteudos.renartlivros.com.br/2P5900/Extracto%20del%20manual%20SCQ.pdf

Scott, R. A., Lagou, V., Welch, R. P., Wheeler, E., Montasser, M. E., Mägi, R., Strawbridge, R. J., Rehnberg, E., Gustafsson, S., & Kanoni, S. (2012). Large-scale association analyses identify new loci influencing glycemic traits and provide insight into the underlying biological pathways. *Nature Genetics*, *44*(9), 991.

Silva, R. R., Alpert, M., Pouget, E., Silva, V., Trosper, S., Reyes, K., & Dummit, S. (2005). A rating scale for disruptive behavior disorders, based on the DSM-IV item pool. *The Psychiatric Quarterly*, *76*(4), 327–339. https://doi.org/10.1007/s11126-005-4966-x

Squires, J., Potter, L., & Bricker, D. (1995). *The ASQ user’s guide for the Ages & Stages Questionnaires: A parent-completed, child-monitoring system.* Paul H Brookes Publishing. https://psycnet.apa.org/record/1997-36683-000
